# Massive expansion of P-selectin genes in two Venerida species, *Sinonovacula constricta* and *Mercenaria mercenaria*: evidence from comparative genomics of Bivalvia

**DOI:** 10.1186/s12864-022-08861-6

**Published:** 2022-09-19

**Authors:** Yuanfeng Xu, Xumeng Dong, Shuonan Ma, Cheng Luo, Jilin Xu

**Affiliations:** School of Marine Sciences, Ningbo University, Ningbo, 315020 China

**Keywords:** P-selectin, Bivalvia, *Sinonovacula constricta*, Gene expansion, Environmental stress

## Abstract

**Background:**

P-selectin is a molecule participating in the inflammatory response through mediating cellular adhesion and essential for wound repair. However, studies regarding P-selectin in Bivalvia are rare. This study identified 90 P-selectin genes among nine bivalve genomes and classified them into 4 subfamilies according to phylogenetic analysis.

**Results:**

Notable P-selectin gene expansion was observed in two Venerida species, *Sinonovacula constricta* and *Mercenaria mercenaria*. The synteny analysis revealed that P-selectin gene expansion was mostly caused by tandem duplication. In addition, the expression profiles of P-selectin genes in *S. constricta* showed that many P-selectins were specifically highly expressed in the gills, and the P-selectin expression patterns changed dramatically under low salt stress and ammonia nitrogen stress.

**Conclusions:**

The massive expansion of P-selectins may facilitate the tolerance to environmental stresses. This study sheds light on the characterizations and expression profiles of P-selectin genes in Bivalvia and provides an integrated framework for further investigation of the role of P-selectins in the environmental tolerance of bivalves.

**Supplementary Information:**

The online version contains supplementary material available at 10.1186/s12864-022-08861-6.

## Background

P-selectin serves as pattern recognition receptor to activate encapsulation and inflammation by mediating leukocyte adhesion at wounds [1]. In *Mus musculus*, individuals deficient in P-selectin fail to recruit neutrophils and are highly susceptible to bacterial infection [2]. In *Homo sapiens*, the thrombus surface expresses P-selectin to recruit leukocytes, which stabilizes the thrombus structure [3]. P-selectin was first identified in vertebrates [4] and then, together with P-selectin-like genes, was widely reported in invertebrates such as *Ciona intestinalis* [5], *Pocillopora damicornis* [6], *Schmidtea mediterranea* [7], and *Ruditapes philippinarum* [8]. P-selectin belongs to the C-type lectin superfamily of proteins [7], and C-type lectins in invertebrates——*Bombyx mori*, *Drosophila melanogaster* [9], *Manduca sexta* [10], and *Mythimna separata* [11]——have expanded on a large scale. Such massive expansions of C-type lectins were associated with innate immune responses to various pathogens and environmental stresses [9–11].

Gene expansion is often related to environmental adaptation [12]. For instance, in *Chlamys farreri,* the expanded Cu/Zn superoxide dismutase (SOD) family genes are considered to protect the body against paralytic shellfish toxins (PSTs) [13]. The expanded subfamily G and H of the ATP-binding cassette (ABC) family in *Daphnia pulex* are considered to participate in pollutant efflux and cell defense activities [14]. Many C-type lectins have been identified in invertebrates, including bivalves, while most C-type lectins were not subdivided. Therefore, the abundance of P-selectin and the presence of P-selectin gene expansion in bivalves remain unknown.

Bivalves are a primary species of mariculture worldwide, including economic species such as *Crassostrea gigas*, *Mytilus edulis*, *C. farreri* and *Sinonovacula constricta*, etc. [15, 16]. Except for scallops, most bivalves are normally static animals that attach themselves to, or bury themselves in the sea bed or other submerged surface, and breathe and gather food through their gills [16]. Due to the particular lifestyle of bivalves, abiotic factors such as seawater salinity, pollution, pH value and temperature, considerably impact their survival [17]. Nevertheless, during the long-term evolution process, some bivalves have developed mechanisms to adapt to severe environmental stresses. For example, *S. constricta* responded to high salt stress by regulating the taurine and amino acid metabolic pathways [18]; *Crassostrea hongkongensis* developed a resistance to the ammonia nitrogen toxicity by accumulating glycogen in their gills [19]. Discovering gene expansion is of great significance for exploring the biological mechanisms of bivalves in response to environmental stresses and understanding the evolution process of bivalves.

In this study, the genomes of nine Bivalvia species—representatives of four orders with large potential economic values—were scanned, and 90 P-selectin genes were identified. Large-scale P-selectin gene expansion was observed in two Venerida species, *S. constricta* and *Mercenaria mercenaria*, and most of these genes were distributed in clusters, which implied that these clustered expanded genes were formed through tandem replication. In order to study whether P-selectins participate in adapting to environmental stresses, the transcriptome data of *S. constricta* under low salt stress, high salt stress, and ammonia nitrogen stress were analyzed. The expression patterns of P-selectins in *S. constricta* showed specific changes under different environmental stresses. This study provides the basic information and lays the foundation for further investigating the adaptive environmental function of P-selectins in Bivalvia.

## Results

### Identification and structures of P-selectin genes in Bivalvia

**Fig. 1:**
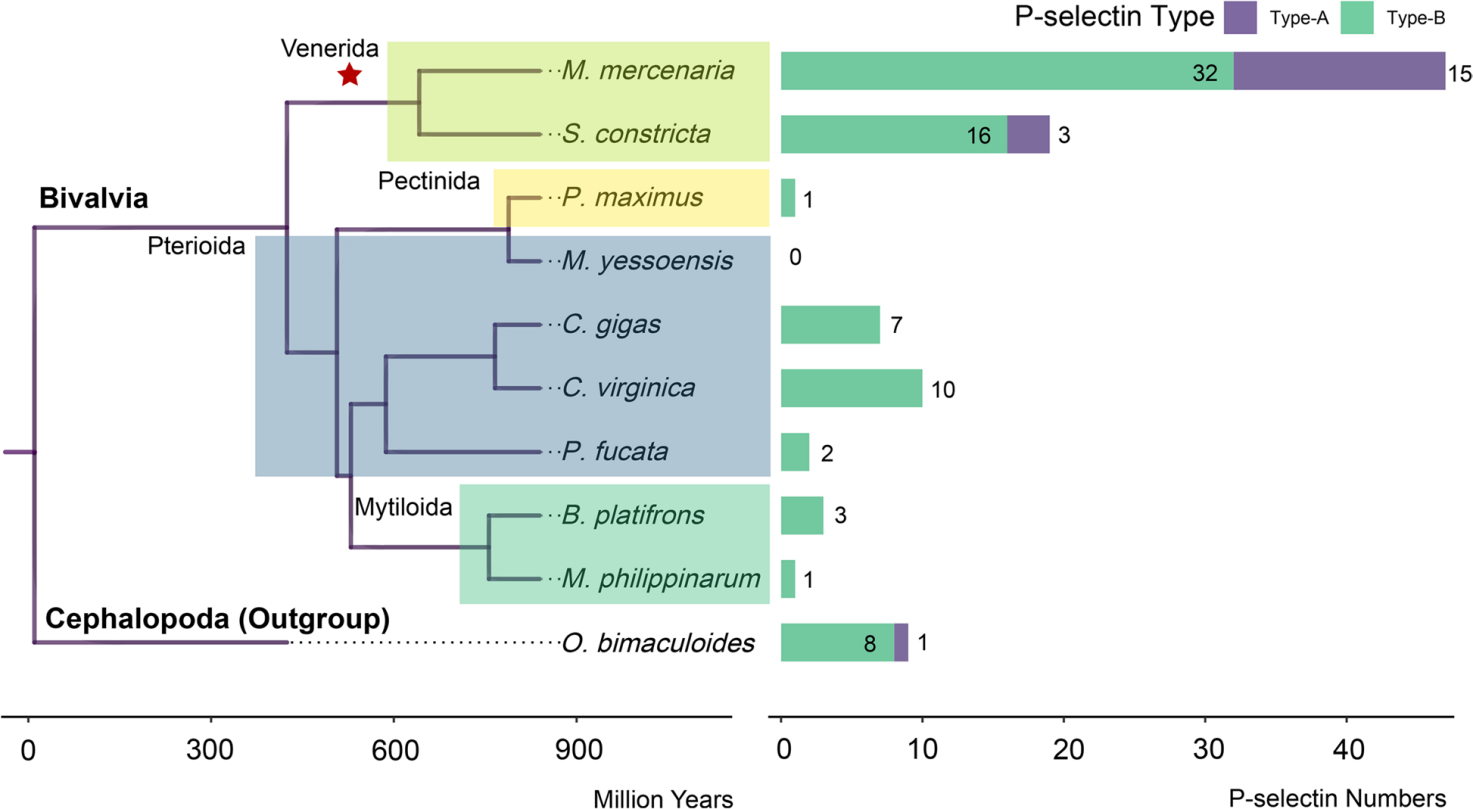
The ultrametric tree, gene family expansion and contraction analyses of *M. philippinarum*, *B. platifrons*, *S. constricta*, *M. mercenaria*, *P. maximus*, *M. yessoensis*, *P. fucata*, *C. gigas*, *C. virginica* and *O. bimaculoides*. Species of the same order were shaded with the same color

Herein, a total of 1, 3, 19, 47, 1, 2, 7 and 10 P-selectin genes were identified in *Modiolus philippinarum*, *Bathymodiolus platifrons*, *S. constricta*, *M. mercenaria*, *Pecten maximus*, *Pinctada fucata*, *C. gigas* and *Crassostrea virginica*, respectively, while no P-selectin gene was identified in *Mizuhopecten yessoensis* (Fig. 1 and Additional file 1: Sequence S1). The number of P-selectin genes accounted for 0.0027, 0.0089, 0.0664, 0.0773, 0.0025, 0.0065, 0.0111, and 0.0166% of whole-genome protein coding genes in *M. philippinarum*, *B. platifrons*, *S. constricta*, *M. mercenaria*, *P. maximus*, *P. fucata*, *C. gigas* and *C. virginica*, respectively. The CAFE output result showed that the P-selectin genes of the Venerida branch had expanded significantly (*p* < 1e-5) compared with adjacent branches (Fig. 1). This result indicated that P-selectin genes had expanded in the genomes of Venerida species, including *S. constricta* and *M. mercenaria*.

**Fig. 2:**
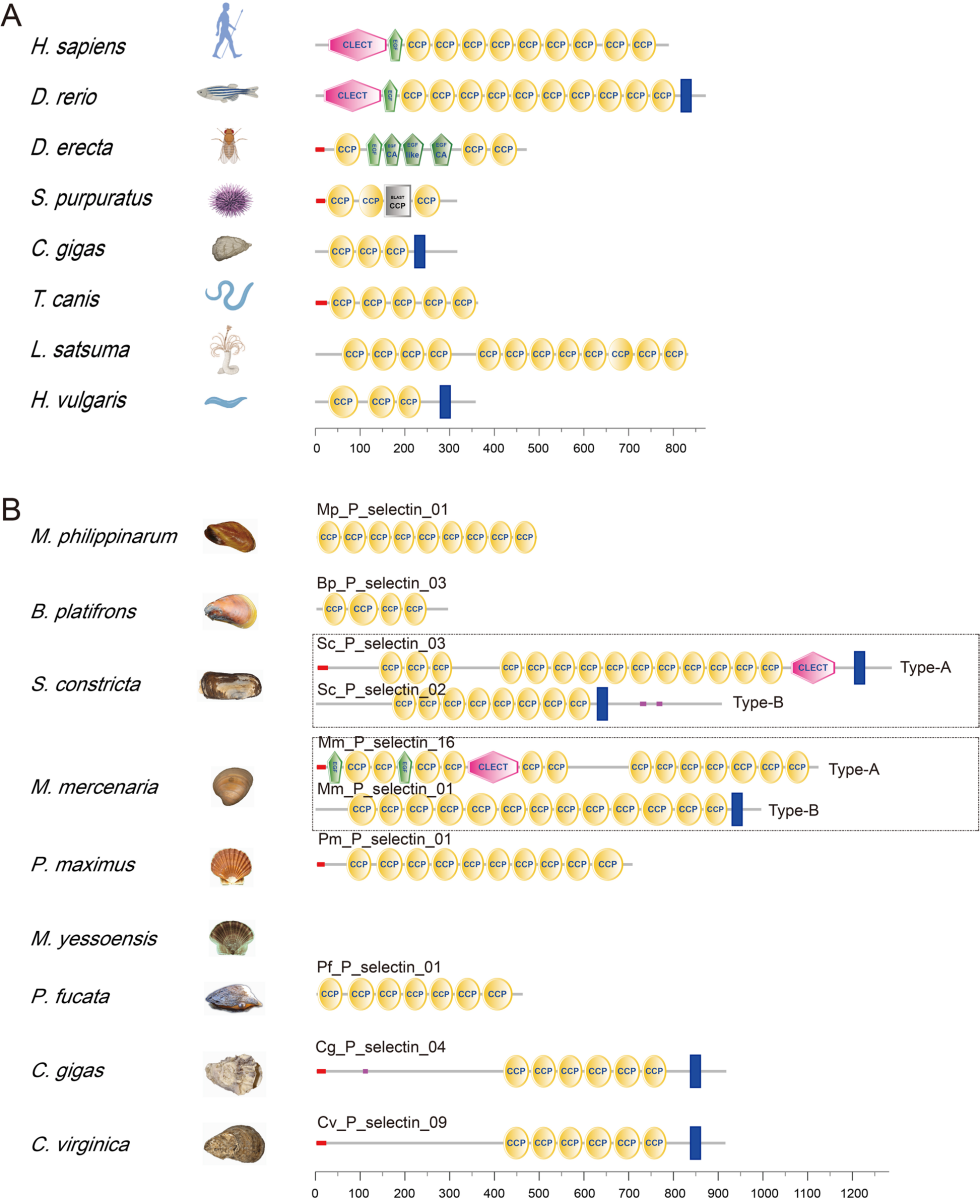
The functional domain analyses of P-selectin proteins. **A** Functional domains of P-selectin proteins published in the NCBI database. **B** Functional domains of P-selectin proteins identified from *M. philippinarum*, *B. platifrons*, *S. constricta*, *M. mercenaria*, *P. maximus*, *M. yessoensis*, *P. fucata*, *C. gigas* and *C. virginica*

SMART analyses revealed that the functional domains of known P-selectin proteins were dominated by complement cofactor protein (CCP) domains; parts of these proteins contained C-type lectin-like (CLECT) domains and epidermal growth factor (EGF) domains (Fig. 2A). P-selectins were classified into type-A and type-B based on the functional domains. In addition to CCP domains, type-A P-selectins contained CLECT domains or EGF domains, while these two domains were not found in type-B P-selectins. The majority of P-selectins in the nine bivalves belonged to type-B. However, 3 type-A P-selectins were identified in *S. constricta* and 15 type-A P-selectins were identified in *M. mercenaria* (Fig. 1 and Fig. 2B). The above results indicated that the expansion of P-selectin genes and the presence of type-A P-selectins might be related to specific functions in Venerida.

### Comparative phylogeny of P-selectin genes in Bivalvia

**Fig. 3:**
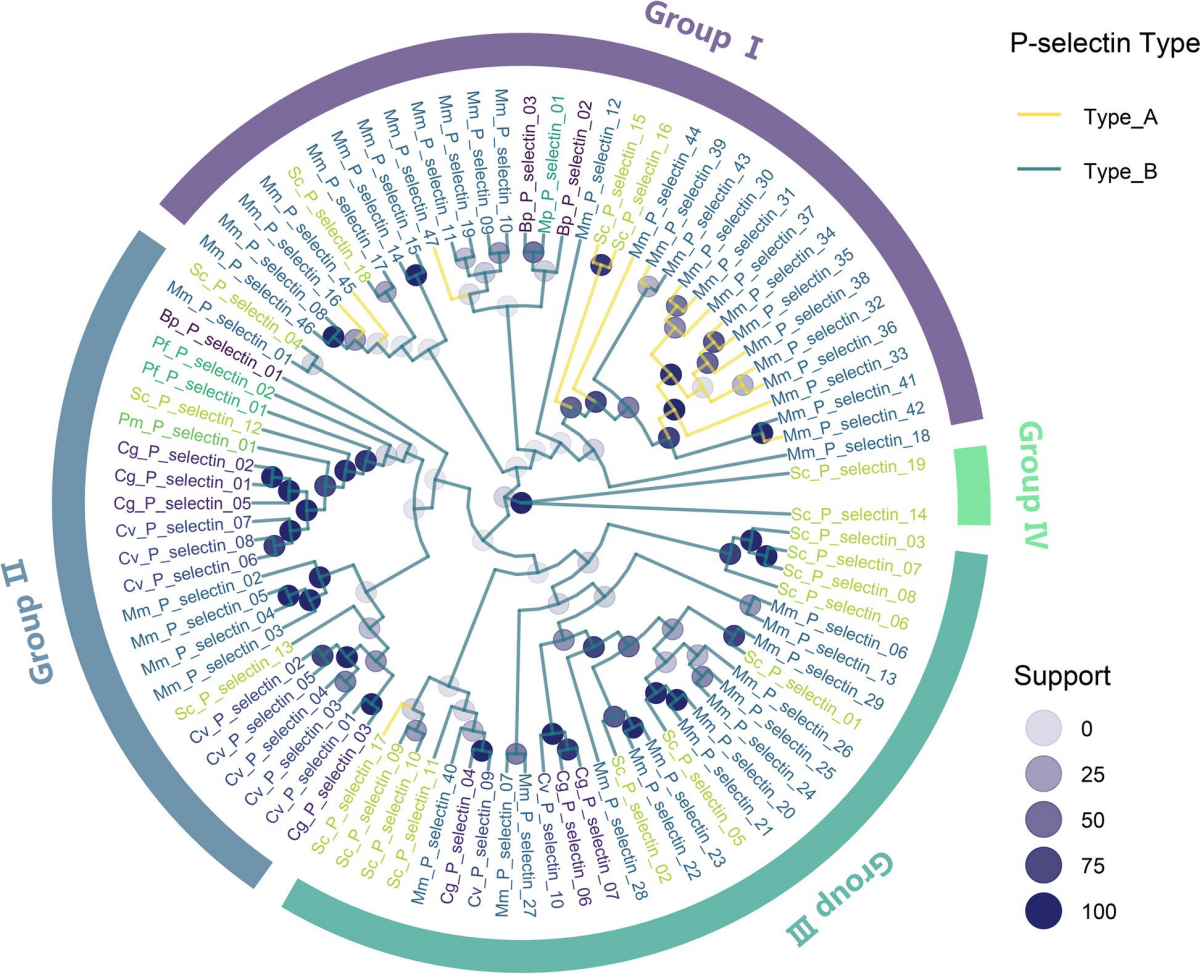
The phylogenetic tree of P-selectin genes. *M. philippinarum*, *B. platifrons*, *S. constricta*, *M. mercenaria*, *P. maximus*, *P. fucata*, *C. gigas* and *C. virginica* genes are distinguished by different colors. Subfamilies I, II, III, and IV are highlighted in purple, dark blue, dark green and light green, respectively. Branches marked with yellow represent type-A P-selectins and dark blue branches represent type-B P-selectins

Phylogenetic analysis showed that the P-selectin genes could be categorized into subfamilies I, II, III, and IV (Fig. 3). 34 P-selectin genes, including 3 from *S. constricta*, 28 from *M. mercenaria*, 2 from *B. platifrons*, and 1 from *M. philippinarum*, were clustered in subfamily I; 24 P-selectin genes, including 3 from *S. constricta*, 5 from *M. mercenaria*, 1 from *B. platifrons*, 2 from *P. fucata*, 1 from *P. maximus*, 4 from *C. gigas*, and 8 from *C. virginica* were clustered in subfamily II; 31 P-selectin genes, including 12 from *S. constricta*, 14 from *M. mercenaria*, 3 from *C. gigas*, and 2 from *C. virginica*, were clustered in subfamily III; 2 P-selectin genes from *S. constricta* were clustered in subfamily IV (Fig. 3). Interestingly, except for one type-A P-selectin (Sc_P_ selectin_17) belonging to subfamily III, all other type-A P-selectins were clustered in subfamily I (Fig. 3).

### Synteny and duplication of P-selectin genes in *S. constricta and M. mercenaria*

**Fig. 4:**
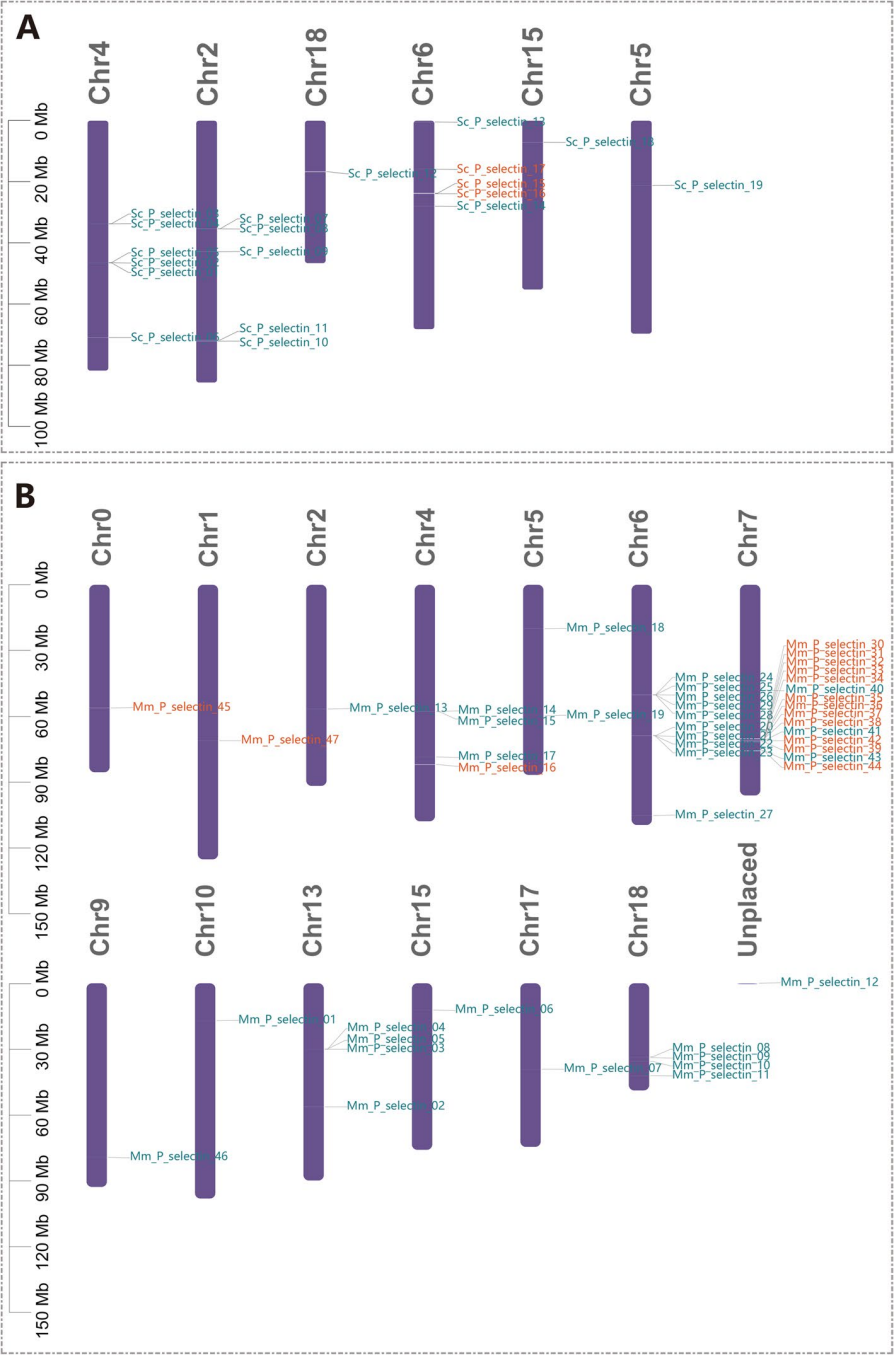
Location of P-selectin genes in genomes. **A** Distribution of P-selectin genes on *S. constricta* chromosomes. **B** Distribution of P-selectin genes on *M. mercenaria* chromosomes. Type-A and type-B P-selectin genes were distinguished by orange and dark blue, respectively

In total, 19 P-selectin genes were mapped to 6 chromosomes of the *S. constricta* genome, and 47 P-selectin genes were mapped to 13 chromosomes and 1 scaffold of *M. mercenaria* (Fig. 4). In *S. constricta*, 63.15% (12 out of 19) of P-selectin genes were demonstrated to be tandem duplications and were distributed in 5 tandem arrays of 2–3 genes. In *M. mercenaria*, 65.96% (31 out of 47) of P-selectin genes were found in tandem duplicated regions, which comprised 7 clusters of 2 to 12 genes (Fig. 4). Interestingly, all 3 *S. constricta* type-A P-selectin genes were located on the same chromosome, and 12 of 15 *M. mercenaria* type-A P-selectin genes were located in 2 adjacent clusters of the same chromosome. The above results implied that tandem duplications of P-selectin genes were common occurrences among *S. constricta and M. mercenaria* genomes.

### Tissue expression pattern analysis

**Fig. 5:**
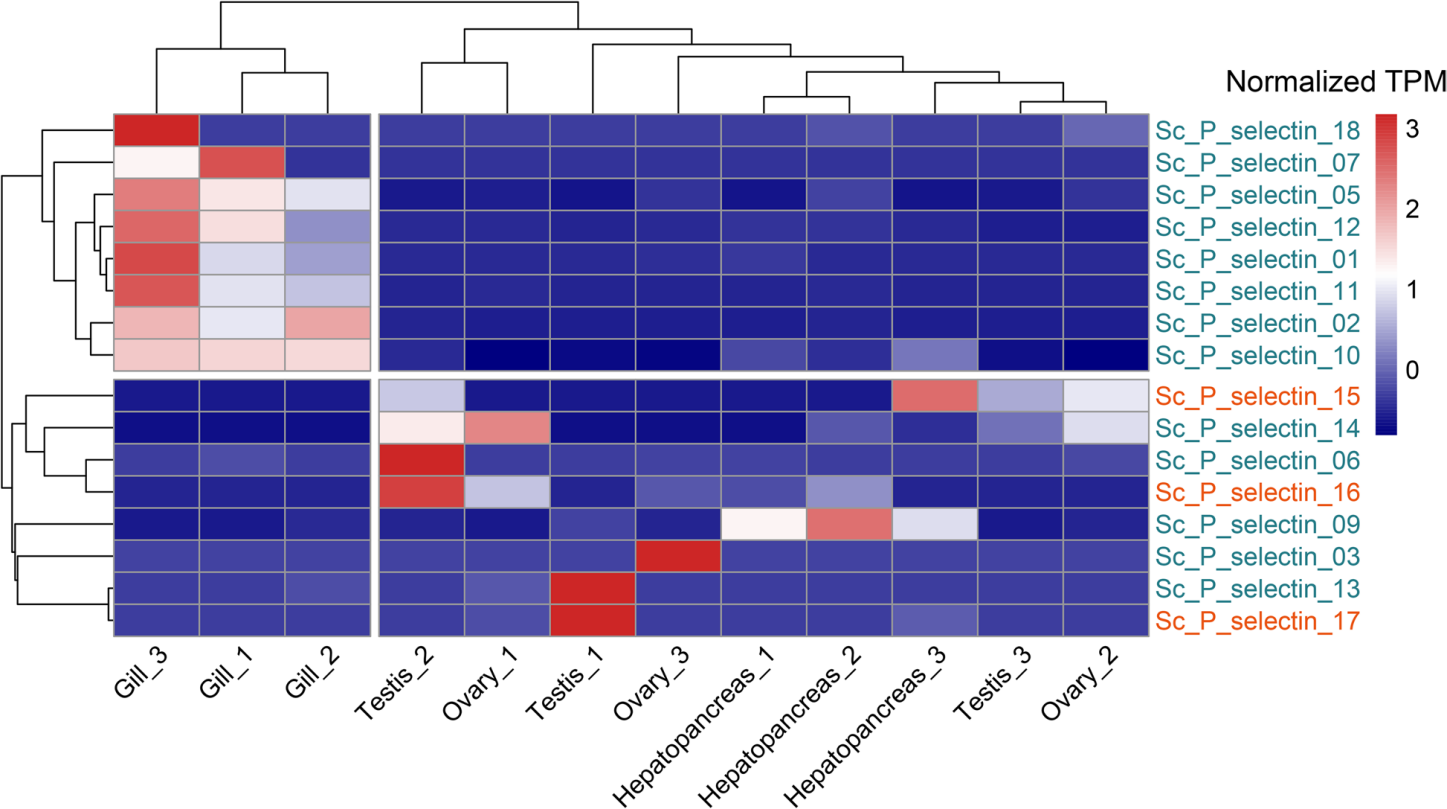
Expression patterns of *S. constricta* P-selectin genes in the gills, testes, ovaries and hepatopancreas. Type-A and type-B P-selectin genes were distinguished by orange and dark blue, respectively

The spatial expression patterns of P-selectin genes were analyzed using the RNA-seq data of different tissues of *S. constricta* collected from the public online database. According to the expression patterns of P-selectin genes, the samples from different tissues were clustered into two groups (Fig. 5). One cluster contained three samples from the gills, and another cluster included nine samples from the testes, ovaries and hepatopancreas. Some P-selectin genes, Sc_P_selectin_18, Sc_P_selectin_07, Sc_P_selectin_05, Sc_P_selectin_12, Sc_P_selectin_01, Sc_P_selectin_11, Sc_P_selectin_02 and Sc_P_selectin_10, were relatively highly expressed in *S. constricta* gills, which was the primary tissue involved in the response to environment stress in bivalves (Fig. 5). The different expression patterns suggested that P-selectin genes might play tissue-specific roles in *S. constricta* gill.

### Expression pattern of *S. constricta* P-selectin genes under acute salt stress and ammonia stress

**Fig. 6:**
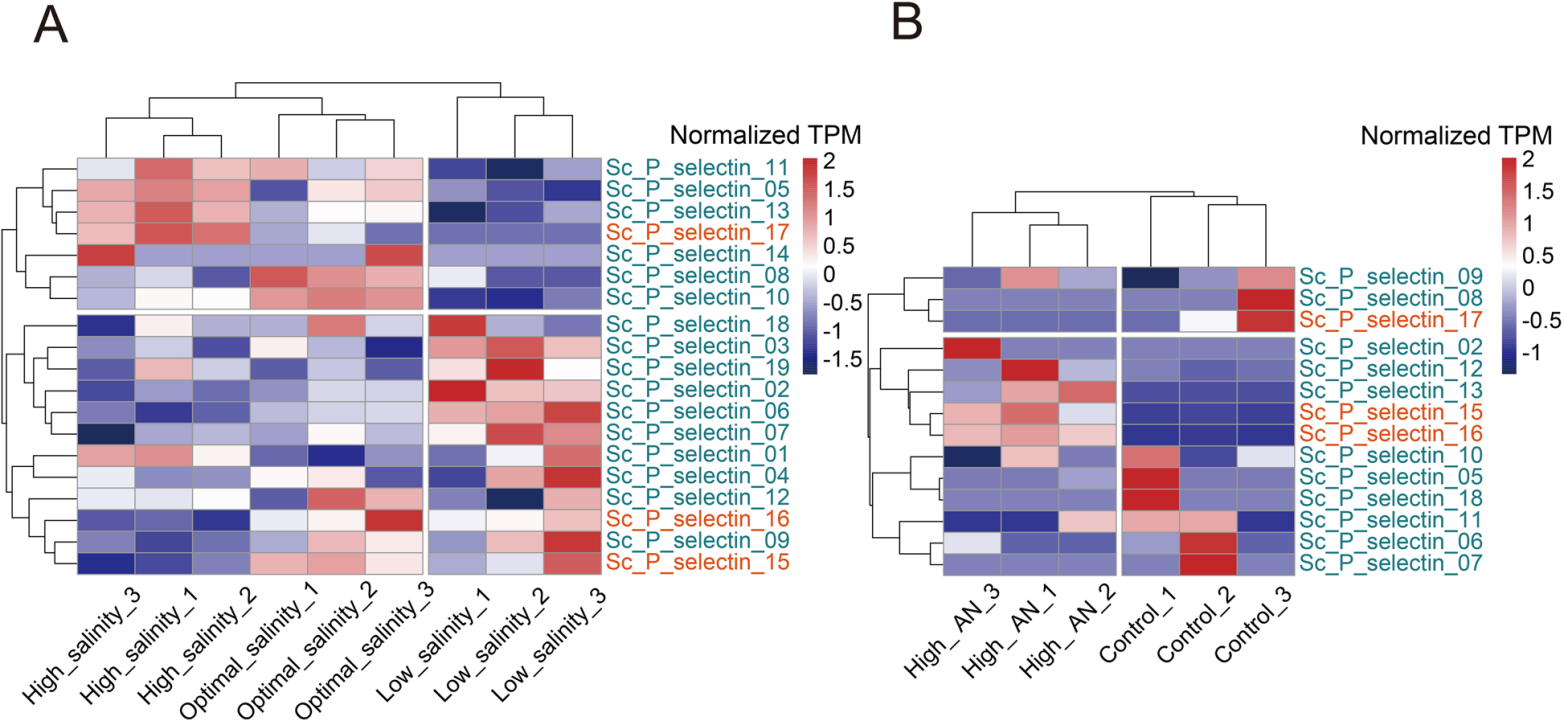
Expression patterns of *S. constricta* P-selectin genes in response to environment stresses. **A** Expression profiles of P-selectin genes in *S. constricta* juveniles under acute salt stress. B Expression profiles of P-selectin genes in *S. constricta* gill under ammonia stress. Type-A and type-B P-selectin genes were distinguished by orange and dark blue, respectively

To investigate the potential function of *S. constricta* P-selectins in adapting to environmental stresses, transcriptomes of *S. constricta* under acute salt stress and ammonia stress were analyzed. Under acute salt stress, 19 P-selectin genes were expressed in *S. constricta* juveniles, among which 3 genes belonged to type-A P-selectins and 16 genes belonged to type-B P-selectins. Under ammonium stress, 14 P-selectin genes were expressed in the gills of *S. constricta*, among which 3 and 11 genes belonged to type-A and type-B P-selectins, respectively (Fig. 6).

Under acute salt stress, samples subjected to low salinity were clustered to one branch, while samples subjected to high salinity and optimal salinity were clustered to another branch. Several P-selectins, Sc_P_selectin_03, Sc_P_selectin_19, Sc_P_selectin_02, Sc_P_selectin_06 and Sc_P_selectin_07, were up-regulated under low salt stress. In contrast, Sc_P_selectin_05, Sc_P_selectin_13 and Sc_P_selectin_17 were up-regulated under high salt stress (Fig. 6A). Under ammonia stress, samples subjected to high ammonia nitrogen and control seawater were distinguished based on the expression patterns of P-selectin genes. Sc_P_selectin_13, Sc_P_selectin_15 and Sc_P_selectin_16 were up-regulated under high ammonia nitrogen conditions (Fig. 6A).

The results indicated that P-selectins were responsible for diverse responses to environmental stresses, and P-selectins might play roles when *S. constricta* face severe environments such as acute variation of salinity and ammonia nitrogen.

## Discussion

In this study, 90 P-selectin genes were identified in nine Bivalvia species (*M. philippinarum*, *B. platifrons*, *S. constricta*, *M. mercenaria*, *P. maximus*, *M. yessoensis*, *P. fucata*, *C. gigas* and *C. virginica*). P-selectins were divided into type-A and type-B according to the functional domains of their proteins. Type-A P-selectins were mainly formed by CCP domains and also contained CLECT or EGF domains, while type-B only contained CCP domains. Compared to the P-selectins in the NCBI database, type-A P-selectins were closer to the P-selectins in vertebrates, while type-B selectins were consistent with the known invertebrate P-selectins. Thus, we speculated that the more common type-B P-selectins might represent the early form of P-selectins, whereas the type-A P-selectins in *S. constricta* and *M. mercenaria* had evolved to become closer to the P-selectins in vertebrates.

During gene expansion, the positive selection pressure promotes the formation of novel functional genes, which allow species to adapt to various biological or abiotic stresses [12]. The tumor necrosis factor (TNF) and receptor (TNFR) superfamilies in *C. gigas* are significantly expanded, which could provide resistance to high temperature and air exposure [20]. The expanded heat shock protein 70 (Hsp70s) family in *Patinopecten yessoensis* is involved in defending against PSTs produced by *Alexandrium catenella* [21]. In the present research, significant P-selectin gene expansion was observed in two Venerida species, *S. constricta* and *M. mercenaria*, and most of the expanded P-selectin genes were clustered. According to the judgment standards of tandem genes [22], these clustered P-selectin were caused by tandem duplication, which was similar to numerous Toll-like receptor (TLR) genes in *S. purpuratus* [23]. Some P-selectin genes were found to be scattered across chromosomes, which was consistent with the Iris genes expansion in *Drosophila melanogaster* through the “cut and paste” mechanism [24]. Therefore, both the “cut and paste” mechanism and tandem duplication could be involved in P-selectin gene expansion.

To investigate the biological functions of these expanded P-selectins, we analyzed the spatial expression patterns and expression patterns under abiotic stresses of *S. constricta* P-selectins using the available transcriptome data [25, 26]. The expression profiles of P-selectins in the gills differed with profiles in the hepatopancreas, testes and ovaries, and some P-selectins (Sc_P_selectin_18, Sc_P_selectin_07, Sc_P_selectin_05, Sc_P_selectin_12, Sc_P_selectin_01, Sc_P_selectin_11, Sc_P_selectin_02 and Sc_P_selectin_10) were specifically highly expressed in the gills. The gills of bivalves, with large surfaces for gas exchange and food filtering, are frequently in contact with the surrounding aquatic environment and thus highly exposed to various environmental pressures. Meanwhile, the gills of bivalves exhibit several mechanisms that control functions related to maintaining homeostasis in response to adverse environmental influences [27]. The specific expression profiles of P-selectins in *S. constricta* gills suggested that P-selectins might play major roles in the adaptive mechanism to environmental stresses. In the acute salt stress experiment, juveniles of *S. constricta* in the low salinity group were clustered to a single branch based on P-selectin expression. The expression patterns of P-selectins in the low salinity group were different from those in the high salinity and optimal salinity groups, and multiple P-selectins were specifically up-regulated under low salt stress. In the ammonia nitrogen stress experiment, the expression patterns of P-selectins in the gills of *S. constricta* in the high ammonia nitrogen group were different from those in the control group, and the expression patterns of the two groups were clearly clustered into two different branches. In *H. sapiens*, numerous studies have demonstrated the relationship between P-selectin and environmental factors: strenuous exercise under high temperatures could produce physiological pressures on the endothelial system, resulting in significantly increased P-selectin expression [28]. Exhaust diesel exposure induced P-selectin overexpression, which could not only trigger the acute atherothrombotic process, but also promote the early stages of the chronic atherosclerotic process [29]. Exercise at high altitudes could cause endothelial injury and stimulate the up-regulation of P-selectin [30]. Abiotic stresses such as acute salt stress and ammonia nitrogen stress cause damage to bivalves. The decrease of salinity caused by rainstorms and the increase of ammonia nitrogen caused by pollutants could lead to large-scale shellfish death [31, 32]. The expanded P-selectins in *S. constricta* could exert functions in adapting to the damage caused by environmental factors and provide a stronger injury repair ability by participating in the inflammatory response.

## Conclusions

In conclusion, 90 P-selectin genes were identified in Bivalvia and were found to expanded significantly in two Venerida species——*S. constricta* and *M. mercenaria*——with strong tolerance to environmental stresses. The environmental tolerance of *S. constricta* and *M. mercenaria* might be related to the massive expansion of P-selectins, which were involved in the inflammatory response and promoted the repair of injury caused by environmental factors. Herein, P-selectins in bivalves were identified at the whole-genome level, which laid a foundation for the following study of P-selectin in Bivalvia, whereas most previous studies on P-selectins so far mainly focused on vertebrates.

## Methods

### Identification of P-selectin genes

The *S. constricta* genome and annotation data were obtained from our previous study [33] (accession number: GCA_007844125.1). The genome data of *C. gigas*, *C. virginica*, *M. mercenaria*, *M. yessoensis* and *P. maximus* were downloaded from the National Center for Biotechnology Information (NCBI) database (accession number: GCF_902806645.1, GCF_002022765.2, GCF_014805675.1, GCF_002113885.1 and GCF_902652985.1). The genome data of *B. platifrons*, *M. philippinarum* and *P. fucata* were downloaded from the websites [34, 35]. In additon, *Octopus bimaculoides* (a cephalopod mollusk) was selected as the outgroup of bivalves and the genome data of *O. bimaculoides* were downloaded from the NCBI database (accession number: GCF_001194135.1). To identify P-selectin genes, the SwissProt databases were adopted to annotate proteins from the bivalve genomes using the Double Index Alignment of Next-generation Sequencing Data (DIAMOND) with an expected value threshold <1e-5 [36]. The potential P-selectin genes were further confirmed by the online tool SMART [37].

### Gene family expansion and contraction analysis

Single copy orthologue genes were identified in the genome data of *S. constricta*, *C. gigas*, *C. virginica*, *M. mercenaria*, *M. yessoensis*, *P. maximus*, *B. platifrons*, *M. philippinarum*, *P. fucata* and *O. bimaculoides* using OrthoFinder [38]. The maximum-likelihood (ML) algorithm in RaxML program with the PROTGAMMALGX model was used to analyze the phylogenetic relationships of those bivalves based on 250 single copy orthologue genes [39]. The ultrametric tree was constructed by the r8s program according to the above phylogenetic relationships and divergence time information from previous studies [40–42]. The numbers of P-selectin genes and the ultrametric tree were input into the Computational Analysis of Gene Family Evolution (CAFE) [43] with an expected value threshold <1e-5 to analyze the expansion and contraction and the output result was visualized by the ggtree R package [44].

### Functional domains analysis of P-selectin proteins

The P-selectin protein sequences of *Homo sapiens*, *Danio rerio*, *C. gigas*, *Drosophila erecta*, *Strongylocentrotus purpuratus*, *Toxocara canis*, *Lamellibrachia satsuma* and *Hydra vulgaris* were downloaded from the NCBI database (accession number: XP_005245496.1, NP_001124070.2, XP_034334851.1, XP_001971405.3, XP_003729029.2, KHN77192.1, KAI0229120.1, and XP_047132808.1). The functional domains of P-selectin proteins were predicted by the online tool SMART [38] and were visualized by the ggtree R package [44].

### Phylogenetic tree construction of P-selectin genes

Multiple sequence alignments were performed using the MUSCLE tool [45]. A Maximum Likelihood (ML) phylogenetic tree was constructed by the RaxML program using the PROTGAMMALGX model with 1000 bootstrap replicates [40], and the ggtree R package [44] was used to display the phylogenetic tree generated from the RaxML program.

### Localization and synteny analysis of P-selectin genes

Location and synteny information of P-selectin genes were obtained from the genome annotations of *S. constricta* and *M. mercenaria*. The Gene Location Visualize (Advanced) [46] was used to display P-selectin genes on chromosomes of *S. constricta* and *M. mercenaria*. Tandem genes were identified as previously reported [22].

### Expression profiling of P-selectin genes in *S. constricta*

Spatial and under-stress expression profiles of *S. constricta* P-selectin genes were analyzed using transcriptome data obtained from previous studies [25, 26]. During the acute salt stress experiment, healthy juvenile *S. constricta* (15th day after fertilization) were divided into three groups: the control group was kept in the optimal breeding seawater (15 PSU), and the two other groups were exposed to 5 PSU (low salinity) and 25 PSU (high salinity) seawater, respectively. Then, juveniles were sampled for RNA extraction at 6 h after acute salt stress. During the ammonia nitrogen stress experiment, two groups of *S. constricta* were subjected to total ammonia nitrogen (TAN) of 180 mg/L (high ammonia nitrogen) and 0.31 mg/L (control) for 72 h (15 °C, 23‰ sea water and pH 8.17). Three replicate tanks were set for each group, with 80 subjects per tank. Then, the gill tissues from each group were quickly collected and used to extract RNA. Raw data of transcriptome sequencing were downloaded from the NCBI database (accession number: PRJNA695103, PRJNA445599 and PRJNA559056). Quality control and filter of raw reads were conducted using FastQC [47] and Trimmomatic [48] software, respectively. Then, the filtered reads were aligned to the *S. constricta* genome using the Hierarchical Indexing for Spliced Alignment of Transcripts (HISATS2) [49]. The transcript expression abundance was calculated as transcripts per million mapped reads (TPM) using StringTie [50]. The TPMs were normalized using the function: scale in the R software, and the global perspective of the P-selectin gene expression level was obtained by the pheatmap R package [51].

## Supplementary Information

**Additional file 1: Sequence S1.** Sequences of 90 P-selectin proteins identified in nine Bivalvia species (*M. philippinarum*, *B. platifrons*, *S. constricta*, *M. mercenaria*, *P. maximus*, *M. yessoensis*, *P. fucata*, *C. gigas* and *C. virginica*).

## Data Availability

All data generated or analysed during this study are included in this published article and its supplementary information files.

## Abbreviations

SOD: Superoxide dismutase
PSTs: Paralytic shellfish toxins
ABC: ATP-binding cassette
CCP: Complement cofactor protein
CLECT: C-type lectin-like
EGF: Epidermal growth factor
TNF: Tumor necrosis factor
TNFR: Tumor necrosis factor receptor
Hsp70s: Heat shock protein 70
TLR: Toll-like receptor
TPM: Transcripts per million mapped reads
