# Supplementary material for: Massive expansion of P-selectin genes in two Venerida species, Sinonovacula constricta and Mercenaria mercenaria: evidence from comparative genomics of Bivalvia
Source: BMC Genomics. 2022 Sep 19;23:662. doi: 10.1186/s12864-022-08861-6 (PMC9484242; doi:10.1186/s12864-022-08861-6)
Supplement: Supplementary file 1 — Additional file 1: Sequence S1. Sequences of 90 P-selectin proteins identified in nine Bivalvia species (M. philippinarum, B. platifrons, S. constricta, M. mercenaria, P. maximus, M. yessoensis, P. fucata, C. gigas and C. virginica). [file 12864_2022_8861_MOESM1_ESM.pdf]

Sequence S1. Sequences of 90 P-selectin proteins identified in nine Bivalvia species (*M. philippinaru*, *B. platifrons*, *S. constricta*, *M. mercenaria*, *P. maximus*, *M. yessoensis*, *P. fucata*, *C. gigas* and *C. virginica*).

>Mm_P_selectin_01
MNFQIYGDDSLIHNSSGTDEYNEDMLTFQLYGLRTFSTITILLPEEHGYSDGGNYYLTLCEVKIYAVPRP
ACDERQTNVTNGQTQIYNNSGKVIDHEYTVGTIIEVVCEEDLHLDGPPRFQCLATGKYNYPVPKCVE
ACVPIPKPSNGHVLPDLLLYEVNTTLKIICDVGYETLKTDTIHCQNDGKWESPIPTCQRVYCLLPRKPDH
GYYSLNGHTEIENVSISRLPYLTEVIGLCDTGYTSNHFQKRICNSDKTWSNQETACTPVQCPHPRNISN
VVYNFKNGSQIVPETTFDYNTVLKVSCKIGYTLIGQTNRTCESDKTWSGKDPNCSVVTCDFPGKFDNG
HYITRYYNTTERNTLTPYLTGKLDFSAVIEAVCEKKFLLKSPNSTRQCTSTGLWDGEKPICNPIMCKWPL
PLENGYYQASNSPTARGMPYNTTLMARCNETYKLSDNYKDRRCNANAVWDRSSAVCEPMCVLPA
VENGQFINVLSTEFALGTTLRFTCDPGFITGSTNDTITCQQNHQWSATPVCQEACGPVPTPVNGHVV
PDLSLYAVNASITVLCDVGYEILKTDTTHCQTGGKWENPIPACQRIQCLVPRKPDHGYYSVNGTAVD
GPLVSKFPYMTEIIGLCNTGYVNTNPQRRICRNDSSWIDLETKCTPLQCSYPQNITNGIYTFQNGSQIF
PKTVFNYNTVLKLSCKTGYTVLGQAERTCKSDKTWSGTEPNCAVVTCDFPGKFDNGHYVIRDHNVT
NMNKLKPYLTGNLNFSAEIKAVCMTHYLLKSTNSIRQCTSGGIWDGDNPVCVHLMCEWPLNIDNGY
YKASNGATTSGMPYNTTLTVSCNEMYRLSGNYRKRRCDEDAVWNNVPAVCEPICILPTVANGKFSN
VSSIHLPSGASVRLTCDSGYRATSHTIICQGNHQWNVTPICQAQSGTRKNTVIYGVVSVVVVMIVAVT
AGLCYCRFIRLRNQKSTDGQKFHLTNNEATDSSDDADNIIRVDVRYKLC
>Mm_P_selectin_02
MNILYIMCNTRNNFLIITVAAALASSQTISTESPHRLQLFTCFSDICNVHGEDQFCSERERKCRNCQEIV
DDCLEPALPQNCTRWCINFHIQKERKNEKETACKDIGLIRNGSHNGSLTQPQVPGDVIAINCDPGYHI
SGSKTLECKVYGQWSHDIPTCQGIKCPMLPPVSYGRHNGSTTIANSLGDVVSVVCNHGYQRIGPDLT
MCSSNGVWSNQLPVCRKIQCPLLHEVENGKHNGSVRTPHVPGDMIVSSCDEVYLLLGTNSTVCNS
QGFWTDPLPRCQVIECTPFPKIKNGKWSGVPHSDIPRFKVSQTIHATCNEGYSLRGPDKWECSVNGN
WYSHDELYMPYCIKDDTNKWMIAACVGGSFIFLALICILLCCLCSRRTGSKLCCLCSRRIGSKQKYQAV
KKHEDKFKNEETTLMIESEDKEKYVERSHLQAKPSPPRYISLQSQRLQRETQSETNRHRAVPRSIPSSSS
TFNELNDGRDKHVEKENSPGKFESNRNGAYSQNIPHPSYVFDASRTQRYEGQNIPGKSGTNGQPED
PPNVQPESFATDTSRAERSEVRNIQGKTGSNRQREDTPNIPSGSFATDVSQRDLHEEQNIQGETGSN
RLREDTPNIPSGCSATDVSQRDRSEKQNVQGETGSDRQREDLPNIPSGSYAFDASQRERSEGPNIPG
PVTFNITLNANNSADNNNLCEYGGRTGSRYQDSKTGGSAPQTPQITQREMNLLKVEEEKSPIEESGEF
QSSQPQTSCVTEDELAAFRSISDPLGSFGSTFTNEKLEDDGNLINGLAVQQSANNEQDTARKLAMPN
GDLNTDDINADVFNEQLGGNGRQINDLAAQQSANMVQETARKLTMPNSDLNTVDTNADVFDEQ
LEGNGGLINGLAVQQSANNEQDIARKLAMPNGDLNTDDINANVFNQQLGYNDRQINGLAAQQN
ANKVQETARKLAMPNGDLNTVDINDDVFDEQLGGNGGLINGLAVQQSANNEQDTARKLAMPNG
DLNTDDINADV
>Mm_P_selectin_03
MKRCVFPFFFLLLGQAVCSQHTKNNFDNELFYCHGDYCNTYGQVEFCSEVFKKCRPCTDVLDDCFT
NQLPTNCTSTCKQHQLEQVTKLAKEKPCHILAEISHGEHNGSILDAHKPGDIIQFTCKPGYRLKGSRHL
ECKEYGIWSKDIPTCEEVVCPVLSNVANGEHNGSSYPLIPGDVVVTSCAYGHQLYGDSTTVCNSNGF
WTHPLPLCELIMCPRPHPIRNGIWPSFPDSTKRYEPGITLNAQCERDYFMYGSALWNCSNEGYWTSE
GRFWPVCFPPGESSKQDCTTYIAWTITLGVVLIVSVSINVIQRCFKQRPRGACQNQDSIEMRKDKWK
DTYVFEGETKQLIDKNDEINEVQKVVSLNESRKAKEKVVPMDTNNIPEPNTVNSHTKEPFYPPSSKEPS
HPPSSKELSQPPSSKDTSQEPPAHVTVVNVYNTNENTNQNTVINRTSDDVINQQTTPKTISADDVTH

INPETTPPNTKEDDKAPVEESGISFQPTQPESSRGIPASLSEFGGRQPGQPESSGNAHAIYSRSPTRDD
APAVNEEEIAVLCVQQTANNEQDIGQKIALPNNEINTDDINV
>Mm_P_selectin_04
MSGHKFRINWITDTVIETMERGVFPFFFLLLGQAVFSQHTKRNFDNELFYCHGDYCNTSQTRCGRSV
ALPRCRPRLNQSLGWWWVGAHWIFLYYSPCDRYFCRCNVTPNEKNNNKLYHFSKRYGQVEFCSEV
FKKCRPCTDVLDDCFTNQLPANCTSTCKQKPCHILEEISHGEHNGSILDAHKPGEIIQFTCKPGYRLKG
SRHLECKEYGIWSKGIPTCEEVVCPVLPNVANGEHNGSSYPLIPGDVVVTSCAYGHQLYGDSTTVCNS
NGFWTHPLPLCELIMCPRPHPIRNGIWPSFPTKRYEPGITLNAQCEQDYFMDGSALWNCSNEGYWT
SEGRFWPVCFPPGESSKQDCTCLAYIATIITLVAGLIVSISINLVQCFKQRARGTLQNYHKKELIEMGNV
NKWKGMDVSEVETEKLIDKIIEIKELQEDVVSLDERRKVKEKVVPMNTNNIPEPNTVNSHSKEPSYPPS
SRDTSQPPSSRDTSQPPSSRDTSRPPSSKEPSQPPSSKEPSQPPSSRDTSQEPPANVTVNVYNINENT
NQNTVTNRTSSNEVINQQTTPKTISADDVTYNNPEPTPPNTNEEDKAPVEESGITFQPAQPESSCGIP
ESLSEFVGRQPCQPESSGNAHAIYSRRPRDDAQVVNEEETAVLYVQQTVNNEQDIGQKVALPNNEI
NTDDINVLSLHY
>Mm_P_selectin_05
MSGHKFRINWITDTVIETMERGVFPFFFLLLGQAVFSQHTKRNFDNELFYCHGDYCNTYGQVEFCSEV
FKKCRPCTDVLDDCFTNQLPANCTSTCKQKPCHILEEISHGEHNGSILDAHKPGEIIQFTCKPGYRLKG
SRHLECKEYGIWSKGIPTCEEVVCPVLPNVANGEHNGSSYPLIPGDVVVTSCAYGHQLYGDSTTVCNS
NGFWTHPLPLCELIMCPRPHPIRNGIWPSFPTKRYEPGITLNAQCEQDYFMDGSALWNCSNEGYWT
SEGRFWPVCFPPGESSKQDCTCLAYIATIITLVAGLIVSISINLVQCFKQRARGTLQNYHKKELIEMGNV
NKWKGMDVSEVETEKLIDKIIEIKELQEDVVSLDERRKVKEKVVPMNTNNIPEPNTVNSHSKEPSYPPS
SRDTSQPPSSRDTSQPPSSRDTSRPPSSKEPSQPPSSKEPSQPPSSRDTSQEPPANVTVNVYNINENT
NQNTVTNRTSSNEVINQQTTPKTISADDVTYNNPEPTPPNTNEEDKAPVEESGITFQPAQPESSCGIP
ESLSEFVGRQPCQPESSGNAHAIYSRRPRDDAQVVNEEETAVLYVQQTVNNEQDIGQKVALPNNEI
NTDDINVLSLHY
>Mm_P_selectin_06
MLYWLFISWFSARTCQSDGQWSGFAPVCNAGRCGPLTSPSNGAVDVSSGTLYTNQAIYTCSTGYSL
VGSSARTCQSDGQWSGSAPVCNAVDCGPLTSPSNGAVDVSSGTLYTNQAIYTCSTGYSLVGSNART
CQSDGNWSGSAPVCSSVDCGPLTSPSNGAVDVSSGTLYTNQAIYTCSTGYSLVGSSARTCQSDGQ
WSGSAPVCNAVDCGPLTSPSNGAVDVSSGTLYTNQAIYTCSTGYSLVGSSARTCQSDGQWSGSAP
VCNAVDCGPLTSPSNGAVDVSSGTLYTNQAIYTCSTGYSLVGSSARTCQSDGQWSGSAPVCNAVD
CGPLTSPSNGAVDVSSGTLYTNQAIYTCSTGYSLVGSSARTCQSDGNWSGSAPVCSSVDCGPLTSPS
NGAVDVSSGTLYTNQATYTCSTGYSLVGSSARTCQSDGQWSGSAPVCNAVDCGPLTSPSNGAVDV
SSGTLYTNQAIYTCSTGYSLVGSNARTCQSDGNWSGSAPVCSSVDCGPLTSPSNGAVDVSSGTLYTN
QAIYTCSTGYSLVGSSARTCQSDGQWSGSAPVCNAVDCGPLTSPSNGAVDVSSGTLYTNQATYTCS
TGYSLVGSSARTCQSDGQWSGSAPVCNAVDCGPLTSPSNGAVDVSSGTLYTNQAIYTCSTGYSLVG
SSARTCQSDGNWSGSAPVCSSVDCGPLTSPSNGAVDVSSGTLYTNQAIYTCSTGYSLVGSSARTCQS
DGQWSGSAPVCNAVDCGPLTSPSNGAVDVSSGTLYTNQAIYTCSTGYSLVGSSARTCQSDGNWSG
SAPVCSSVDCGPLTSPSNGAVDVSSGTLYTNQAIYTCSTGYSLVGSSARTCQSDGQWSGSAPVCNA
VDCGPLTSPSNGAVDVSSGTVYTNQATYTCSTGYSLVGSSARTCQSDGQWSGSAPVCNAVDCGPL
TSPSNGAVDVSSGTLYTNQAIYTCSTDYSLVGSSARTCQSDGQWSGSAPVCNAVDCGPLTSPSNGA
VDVSSGTLYTNQAIYTCSTGYSLVGSNARTCQSDGNWSGSAPVCSSVDCGPLTSPSNGAVDTSTGTL
YSNQALYSCLLGYILVGGSARTCQSSGLWSGSAPSCDSVDCGLLSSPVNGYVQNPVATAYMERASYF
CSTGYNIHGPTDEDMPSDGFGVGQRLLVTL

>Mm_P_selectin_07
MQLLMIQIQVLVTIATSLHFAGCVTFSNSVIYGCKDGFMIGAKCANRTGTENPCGRNTSNVLCYCLM
MQPLNGGINDTDADKVHFTCDYGYKLFGENEQTCLNSGHWSNSVPLCIPKDCELSVPYNAYVDDIQ
NETVYYECIKGYYLPGPNYQTCLHTGKWTNTVSDCVKEDGVACPQIIDREGNVWNKSSPGELSIACPI
GFTGNVTRQCQNGGKWKLPKYGCIRQEVIHISTQAGKLETNSTEEEIQEVIQNITIVTAKPTKEFFGAEL
LELSSALNDIATVLETVPERLNDNITNNFLESASNLIKSSTHEGWKHTLKSLLKRRKLYMKTYYSQIIQLR
SK
>Mm_P_selectin_08
MVEIFTYFRDKFAYVSIDCGDPTPEHGSSNATSTINGTTVSVECEEGYDMVGDDIISCQENASWTAVP
VCKIKDCGYPSVPYTSVSLPNNKTTFGETILISCWTGYTLTGSPTVTCLSNGNWEMMPSCIIVDCEDPT
PIKGSVDKNETKFGTVVEVSCDPGYDITGNSTIICQDDGTWSDYPVCDASDCGLLSVPNGKVNDSQG
TTTGSVAVIECDEGYDIKGTQFATCLDTGTRARWNYSVECEIQVCDNPKPDNGQISKTDGTDIYVFET
YAEIECNTGYKLYGDNVITCEAGGVWSDNTTCMIFDCGSLSAPTNGTMDSQGTKYNDEIKFSCNEGF
LLVGEENSICGETGEWSNDTPECVVKSDVGGSCINEDYCLLAGSQCINSVCTCTTGIYDDRTKKCDTM
PLMPFGEDVGDILVSKRAHCGPKVSFEPGLPMFDKMHTDLYVCASGLVSFDSPFAYPTPPANKLSLVS
LGQQRTIVAPFFGPSDKRESGLLRFRSYDILNSHKKMEKDSDLISYIETIVEKFENLTSYDASFVLIATWYK
MKAIGSGYDKTKVP
>Mm_P_selectin_09
MYAIVFTTKGLAIQVPVPDAKFYKEKLLKYFQKRRPRTDCGPPPSIENGRISGHISSNTFSVQCNDGFIT
TGDGIIKCTSSGKWTYLPYCEQVSCGIPEIPCNGMLLNINGTQSNAMATVKCDEGYSLAGNSVITCQS
NGSWTLIPRCIQIDCGIPNITHGSIIESSSTIYCSKTEVTCNDGYTLIGSDTVQCGIEGQWSTIPTCNFVTC
GSPEIPANGAIASIQGTTFNKTALIKCHAGYVIIGTDVVTCSSNGNWTESPTCTQGIT
>Mm_P_selectin_10
METVHYINYESLRNSVMNRYDALEFTAANKERLDHKNEAERTEEDDKTLKGDQTLNCDVRVVGASQ
FVCECVNLTETVLPVIGKEVETGHCPNFSTNGTVVKTTGSSYGDTIELTCDDRYKLSGTKVITCLSTGN
WNGSPQCIPKVCNNSQLSDNIKFESETESISIETTVTIHCKEEFVLTGNNTLTCLSNEQWSENPICKVID
CGIPESVPNAVMTNISETTYNSIVFVECKEGYIMSGNNTVKCNLSGNWDSMPTCKKVSCGHPTIPGN
GSISRISGTTFQATASLECNEEFFIIGDAIINCLSNGSWSDIPSCQQYGTRSKYAAASNHN
>Mm_P_selectin_11
MAVSEGLHTSEESKESENALIGDKSDCGTKQKYREKKHLIHVKAKCKLALVIGTLLLVLVAITAIVYIITDIS
ERTDNPRHCNTNIKIPNGGFINASEIRQGDTANIQCEVGHKLNGSKKIVCLSSGHWNETPICLPKQCS
KLRLPDHANIELRHLVHRIEEITVTCEAGFVLEGNKTVICHENDTWSKVPYCKPVNCGYPASIKNGIILN
LTDTTYNKTFHVLCNHGYYLRGREVVKCESDGTWGELPKCEQVSCGTPIIPKNGHIKKLDERLVTFKCE
NDFVLVGNVSSTCQSNGEWSTYPECKRACSLDIHIPNGRVLNKAGLNKSHIANIECADKHKLNGSNTI
VCQPSGQWNETPICVRKQCQLFPAPPNAEFSRLQPLISNVGEEVTIFCSKGFQIKGNRTSICQDNETW
SQIPVCKPVDCGHPLNTNFANGVISRLTDTTYNSTFHVRCNNGYNLNGQSIVRCESDGNWSTRPTC
KPVSCGSPNMPENASRQTINGFVFGSSVSFQCKKGFSLTGAKSSTCQSDGKWSVPYPKCEQIKEQPV
YPKKGNNFVRTTASSSTRLSNNISPVKIILFVSVLINWL
>Mm_P_selectin_12
MICNPSGCKKIAAPSHGSIVNKNSTLINDTAEITCDDGYHLNGRNISVCQANGTWSNTDSTCVTGCE
KLPSLTNGIITLSNFTNINSTASFSCYYGYEMRGNDMSTCNDGGWDHLPPTCIAIKCPNPLPPANGLV
SLNNGSTRVNSSVTFSCYPGYKLVGTVTTECILNATWQGITPSCIVQNCLFPSVPDNGYIRNMTSYAV
NSTAVMSCNAGYNLTGNDTIQCMENATWSTLSAVCSLIECPFLQRFVNGRLNISSTNRYVNSTAEIV
CDLGYILDGSLSVECQENGTWDISSAICIPKNCSSPPVPENGYIRIITSYTVNSTAVMSCNAGYNLTGSD

TIQCMENATWSPLLAVCSPLECPLLQALVHGRLNISSTNRLVNDTAEVVCDFRYVLDGSSIVECQENG
TWDISPAICIPKNCTYPLAPKNGYVRSTTGLSMNSTAVIACHVGYNLTGNGTIKCTENATWSTLSAHC
TLIECSAQLSLAHGLLNISLGNRYVNNSASALCDLGYVVGDSTMSATCTQNGTWDISSILCIAK
>Mm_P_selectin_13
MFCLHLDCGKPPPPKKGNVATPNGTKYLSQAVYNCNYGYVLRGESRRVCNNSMDWSGTTPTCLAK
DCGKLSAPSNGNVMTPNGTKYLSQAEYKCIFGYVLRGEIRRICNASGNWSGTSPTCEAKECGKLLAPS
NGNVMTPNGTKYLSQAEYKCNYGYVFRGEVRRICNASGNWSGTTPTCEAKEEIKKVRIYLPEVKSTAK
TARAKHRKSSRIQCNYGYVFRGEVRRICNASGNWSGTTPTCEAKDCGKLSAPSNGNVMNPNGTKYL
SQAEYKCEYGYILQGEIMRICNASGNWSGTTPTCEAKDCGQLSAPINGYVMTRNGTKYLSQAEYSCN
YGYILRGEIRRICSASGNWSGTTPTCGAKECGQLSAPINGNVMTPNGTKYLSQAEYSCNYGYVLLGEV
RNICNASGNWNGTTPTCGAKDCGKLSAPSNGNVMTPNGTKYLSQAEYKCNYGYVLRGEVRRICNA
SGNWSGTTPTCEAKDCGKLSAPSNGNVMNPNGTKYLSQAEYKCEYGYVLHGEIMRICNASGNWSG
TTPTCEAKECGQLSAPINGNVMTPNGTKYLSQAEYKCNYGYVLLGEVRNICNASGNWNGTTPTCGA
KDCGKLSAPSNGNVMTPNGTKYLSQAEYKCNYGYVLRGEVRRICNASGNWSGTTPTCEAKDCGKLS
APSNGNVMNPYGTKYLSQAEYKCEYGYVLHGEIRRICNASGSWSGTTPTCEAKADRMTCLQCRDVY
HTNLCNTVTKCALGQVCYVEKFTTHNGQVRYNTGCADLKVSCFNMSIILSNWSLW
>Mm_P_selectin_14
MYGDHVDRFSVGQWNGTAQELFSKTGNQGKNWIHGNIFIKSTTNFKVFLAGKVKNANYRFKGDIAV
DDVSVMSCPDCHNPAQDHAIVNAANFEYGSVVEVTCNFGYILTGDSHMVCLAGGAWNASRPSCK
SYDCGNGTILNGHFSAPEGTTFGQTATHVCNIGYTLVGDTTVTCTVSGWNGTAATCEIVDCGNRTLL
NGHFSAPEGTTFGQTATHVCDVGYTLVGDTTLTCTVSGWKGTAATCEIVDADNNALIAVYVLVPVTV
VILAVLCTIIFVRRRFQNCKNVSESKHESRKNPVMTASNKTYTGVALESQNYTNHAYEDGSLQLNDEY
ATISDEQKVNSNNINYNIVSAAETSKEHDAHNYDHMKETAAHFVDESYSHVSRNKSGTTFEPSYNTT
LHLDAGNVPLRTHTQSDLNSYDLLNISNCNETAKQRNETDNRKESDYDHAHAGGIDPPALDEDAYS
HLNDTKMKQKCLASFNSGNDHTFEDSNVNENFRKTEKEVEKSEAGYEQERGLASQYELQDTINDDR
HYFVLEAEEVMDEHNGSENHDYFVLEKTES
>Mm_P_selectin_15
MYGDHVDRFSVGQWNGTAQELFSKTGNQGKNWIHGNIFIKSTTNFKVFLAGKVKNANYRFKGDIAV
DDVSVMSCPDCHNPAQDHAIVNAANFEYGSVVEVTCNFGYILTGDSHMVCLAGGAWNASRPSCK
SYDCGNGTILNGHFSAPEGTTFGQTATHVCNIGYTLVGDTTVTCTVSGWNGTAATCEIVDCGNPTPI
YATANSTNFEYGSVVEITCITGYTLAGVSQMVCLDNGNWSTSKPTCIPFDADNNALIAVYVLVPVTVV
ILAVLCTIIFVRRRFQNCKNVSESKHESRKNPVMTASNKTYTGVALESQNYTNHAYEDGSLQLNDEYA
TISDEQKVNSNNINYNIVSAAETSKEHDAHNYDHMKETAAHFVDESYSHVSRNKSGTTFEPSYNTTL
HLDAGNVPLRTHTQSDLNSYDLLNISNCNETAKQRNETDNRKESDYDHAHAGGIDPPALDEDAYSH
LNDTKMKQKCLASFNSGNDHTFEDSNVNENFRKTEKEVEKSEAGYEQERGLASQYELQDTINDDRH
YFVLEAEEVMDEHNGSENHDYFVLEKTES
>Mm_P_selectin_16
MTIMIHGVILFLSFFLFGINAQTNCTLDDDCPVNATCDTTVSECVCPTDYVLYINTCLYDCGNPPDFYV
DGIALTYSLHFYTYLEGDFVYYFCPSSNYSLFGNDIGVCEVGGWTGMNPVCLLDCGTLPEITNGIITLD
DSTNTTVGATATVDCDVVYYPTSTILTCLDTGQWDFAECISGCASDDDCPVNATCNTTASECVCPTG
YVMYIDSCKYDCGTPPDFYADGIALTYLALLDIYLEGHFVSYYCPSSNYSLFGSDTGVCEVGGWTGMN
PVCLLDCGTLPAITNGNITLDDPTNTTVGTTATVDCDVGYNPTTTIMTCLDTGQWESVNCISGPVCP
DGWISNKTSCYFFSTDELNWDQAFEQCIALQSYLVEIEYSAEEDFLTSVVLETPNEHYYIGLREIEGFWK
WMTSGNDYIYAEASNASVCAAIYTDFWPAVNCSTLYRYICEMRYCGVLSITNANLNDSTTSYGTLVEI

TCLEGYTINGNTTVECLADGSWSDSPSCDPVDCNALSVTNANLNDSTTSYGTVVEITCLEGYTINGST
TVECLADGSWSDYPTCDQDPSCDVGEYYNSTQQTCVLCPAGYYCPYNTSVYEYYSCPAGHYCEDGT
EIPMPCPSGTYRADLRGTTNLDCLLCLCGMYCESSGLASPTGNCSAGYFCIERAASSTPSDGVTGDM
CSPGSICPAGSCNETFADCGALSISNANVNDSATTYGTVVEITCKEGYNISGNTTVECQADGVWSGS
AICDPVDCGDGGFLDTTDTNTTYGSVLTLTCTADGYEISGDTTILCQADGFWNGTATCLPVDCGELSI
PNANVNDSATTYGTVVEITCKEGYNISGNTTVECQADGVWNGSAICDPVDCGSQFNISDGTISLTND
SDTTYGASAEVNCDYGYETNASTIYCQIFGVWENSVCTKKDCGPVPEIMYGNATLSVENETTFGAQA
TVVCETGYTTEIVNNLYEVFHIYCSGSGEWDNRTCSITDCGQLPNDTFYGELTLDNEGITTYGATATLK
CVTGYETSMPNITCQTLGAWSEFENCVVKDCNAVPNIYKGTVILAEDGNTTYGALANVTCEEGYNTS
TEIIQCLDTGTWENTTCEKIDCGMPPNDTMYGELSSRQ
>Mm_P_selectin_17
MDVPDIAKGTVALLEDSNTTYGALANVTCDQGYNASYPKTILCPDTGKWEATTCEIVDCIDVQDIAK
GTVTLLEDSNTTYGALANATCDQGYNASPKTILCFDTGKWETTTCEIVDCMDVPDITKGTVALLEDSN
TTYGALANVICDDGYNASSKTIQCLGTGKWEITICEIVDCKIVSDIPNGTITLLEDGNTTYGALANVSCEI
GYNASVDIIKCTKAGMWEISTCEIIACKAAPAIPNGIISLRKEENTSYGAYANVFCDEGYNASTEIIYCRET
GNWDATECKIIDCGELGNPTNGAVNITKGTTYGATASYSCDESFNLVGDETRVCRQSGKWSNMEPD
CVQQGTVLSVSTVIY
>Mm_P_selectin_18
MHRNIILSIICFAGVVAGNYNQKYFEILQGTKVVNVVSYFNDISILECGKNCIEYAQCVQAGYNTDSQT
CQISSDNTTVTDPDSVIIKFSGVCGSPQTPRNGNVEISSDGKIASFSCDNDYTLAGTEILHCMHGSWN
GTVPTCTGADCGSPLLPANGDHFSYTGTAVNDVATLVCLPGFAPTVNSVTCHTDGQWESTSCTEVC
GSLQTPENGNVEISCDGKIASFSCDNDYTLVGTGNLHCMHGSWNGTVPNCTGADCGSPLLPANGD
HFSYTGTALNDVATLVCFPGFAPTVNSVTCQADGQWESTSCTEDCGTALETDYINVRVSYPLGSQAG
ATATVACAPGYKSDSGLMSWAFTCDAIGSQWKITQAPDCIWELMFRGSAENGYNVTNAFLFGYGS
DGVTDEEGCKDVYNTSCSLNYRNSKVDQWGLLNIQEVTYELYDTTTNVLSVRFGGSGSTINNWFSTS
RVIEHPWSITVLTNTWWDLNGPTDIMYGRSFRMTAGYDIDAVPCQDHFYTVVFFPPLYSCSYDAFDN
YPKFTYSALTGGSIYSYETFYTAATMGIWVKYY
>Mm_P_selectin_19
MVTNFKVRTLFSCLANGSWTDVPTCTHKECHLFVIPENAESTPNKSQFLYSENVTIKCNIGYELQGIETI
SCQTDGTWKEVPTCSPVECELFDVPNKAKLEPDKQQYTYSETVTLECGSGYEIQGNSAVRCQENAS
WTEIPRCKPIDCSTLQLPEHAEKDLNITDLNFDDAVFITCENGFILVGDSNISCYADGTWPTMKCVPVN
CSTDDFAKPDNSMVSNVSDSAFEIECVEGYIIEGNSSVTCISGNWSEFPRCNKVDCGAYRFIENGNVS
SETGTKYGDTVEILCIEHFRLIGKSRGICLSNGSWTDKPECTIIECGTLRIPENAEEPVNINDTIEGTVITLV
CRDGYDLNGNGTVICKANESWSDIPMCEPKDCGQPDETTNGNFIGLDKTTFNSSFVVECMDGYNLI
GEHLVYCQENGSWSGVPMCEPKDCGQIEPPENGKHC
>Mm_P_selectin_20
MATYFCNPGYTMSGQQYPYCQNDGTWNETAPTCIKCPDLTDPASGTVTINTDGVTSVAQYACASE
YDISGETASICLTDGTWDNAPPTCSCSPASVPADGSVLSDNGESVYYQCNLNYSMNGTSVRECSAD
GTGWSGSDPICVPCENLVSVPGGSFSPTTDGVNTKVEYSCDVGYTISGKKEITCNSDGTWDSSQSTCT
QCDTLTASSGGNFTTYSDDSTVTKAIFTCDVGYTLDGYSELTCEETGQWDNEIPQCVECKARSDPTSG
NLTLTTDGQTTTGIYSCADGYTIVGQQTISCRIDGNWNYPSSECECETPSAPVNGNVSADGKTVIFYC
DTGFTMSGESSGNCNNDGTGWSIPTPQCVQCESLDSFTSGNVTYVSDGSTTVVEYSCPEGSTLQGT
PLVSCNASGNWETEIPECVSCPTLPSVSVSTGSVSLATNGTATTAEYSCPQGYEVKGLQTLTCNSDGT
WSDDPSDCVCETPTIPEGGNYTISEDGLSVNFSCGEGYTMEGEQTISCGTDGSGWALVYPNCTKCEE

LVTPSNGNITLVTSGTETTALYSCGVGFTLEGSAGPTCQSDGTWTTTEQTCVSCPDLPSITVTNGSVSL
ESNGTTTVAVYSCPTGYEVSGPETLTCNSSGVWDSTPSTCVCETPDIPVGGNYALSDDGLSVTFTCGE
GYTMNGVQEISCATDGSGWTLIFPNCTQCDSLTPPSGGNVSLVTNGTQTVAVYSCGVGSSLDGEPT
PGCQEDGTWKSTEPTCVTCPSLPDVSITTGNVTLETDGTTTTAIYNCPEGYEVSGSQNISCNTDGTWS
SSPSDCVCITPAFTEGGNFTLSDDGLTVTFTCEFGYTMVGEQIITCGTDGSGWDISQPNCTKCEDLTTP
SGGEVALITSGTQTTAVYTCGVGYTLDGQPTPECGTDGTWSAEGPVCVSCPEAANITSGILNVTTDG
QTTTAVYTCDSGYEMDGNAVLSCLTNGTFDNEPPICFCEAPPSPLFGNVLSNKTTAVYSCEEGYILKGT
SARTCQDDGLGWTDTAPSCITCATLVSPADGSYSLTTNGTVTIATFGCDVEYSMNGTQILACLDDGS
WSEQAPNCVKCPTLTQPNSGSLVISSDNVTTSAKYTCSSSYYIVGEDNLLCSSEGEWSASEPFCACNN
VTAPDNGNVDISIENGTAIYTCELGYSIDGVVTRTCANDGSGWSHTQPSCVGCPNITFTADGNSEIVS
DGTVTSVNFKCDKGATIDGPVSLTCQSDGTWTSLQPTCVSCPELDAPSSGNITVDTDGQISTVTYTCA
DSYEVVGEATQQCQTDGTWSGSNTTCVCVAPTPPANGTITLENNNLLAVYTCDIYFSMMRDSERTC
QSDGSGWTGSSPTCVQCAPLISPFNGSLFTDTNSTHTISTFVCSLGTSMQGEATSVCMNDATWSSTE
PICVTCPSLLDPSSGNVTLSTDGMTTTAEYACASGYEVSGQSVSTCLADGSWSYTTPECLCEDPSSFA
NGTVTSDGRDANFQCNVGYSLLGTSGIACNNDGTGWSEQFPECIKCNDLNTPSGGNVVFTSTGVTT
VAGFLCDVGATLNGSTSLTCHVDGSWNGTAPSCVSCEVLASPANGEYVLSTDGTTTTIQLQCNDGY
EAAGTTNIECGTDGQWIYSKQSTCDKKEEVANSLLGALIGVSIASLLIITVLSILLARFVYLYLQRKSGGD
KYTTNVTLFDSAGKFKYSDDEFPFNPVKLTRSAAPVNHERPVFDISGPFSTSPAPQGFENHVTGHEAV
PESPLPIETHRSGNSIISFRRSTTTLTDKSLSSVTAITIESAKETPAITAPLAKNINGLPPIKNGTTPLQEPELK
PRRLSPIPKHMQRRGKLSPSSPQINKVFKESKSDAEGRQSPFEGRSPIVMSHPLNVTDFESQSLDSVSM
VGE
>Mm_P_selectin_21
MATYFCNPGYTMSGQQYPYCQNDGTWNETAPTCIKCPDLTDPASGTVTINTDGVTSVAQYACASE
YDISGETASICLTDGTWDNAPPTCSCSPASVPADGSVLSDNGESVYYQCNLNYSMNGTSVRECSAD
GTGWSGSDPICVPCENLVSVPGGSFSPTTDGVNTKVEYSCDVGYTISGKKEITCNSDGTWDSSQSTCT
QCDTLTASSGGNFTTYSDDSTVTKAIFTCDVGYTLDGYSELTCEETGQWDNEIPQCVECKARSDPTSG
NLTLTTDGQTTTGIYSCADGYTIVGQQTISCRIDGNWNYPSSECECETPSAPVNGNVSADGKTVIFYC
DTGFTMSGESSGNCNNDGTGWSIPTPQCVQCESLDSFTSGNVTYVSDGSTTVVEYSCPEGSTLQGT
PLVSCNASGNWETEIPECVSCPTLPSVSVSTGSVSLATNGTATTAEYSCPQGYEVKGLQTLTCNSDGT
WSDDPSDCVCETPTIPEGGNYTISEDGLSVNFSCGEGYTMEGEQTISCGTDGSGWALVYPNCTKCEE
LVTPSNGNITLVTSGTETTALYSCGVGFTLEGSAGPTCQSDGTWTTTEQTCVSCPDLPSITVTNGSVSL
ESNGTTTVAVYSCPTGYEVSGPETLTCNSSGVWDSTPSTCVCEAPPSPLFGNVLSNKTTAVYSCEEGYI
LKGTSARTCQDDGLGWTDTAPSCITCATLVSPADGSYSLTTNGTVTIATFGCDVEYSMNGTQILACLD
DGSWSEQAPNCVKCPTLTQPNSGSLVISSDNVTTSAKYTCSSSYYIVGEDNLLCSSEGEWSASEPFCA
CNNVTAPDNGNVDISIENGTAIYTCELGYSIDGVVTRTCANDGSGWSHTQPSCVGCPNITFTADGNS
EIVSDGTVTSVNFKCDKGATIDGPVSLTCQSDGTWTSLQPTCVSCPELDAPSSGNITVDTDGQISTVT
YTCADSYEVVGEATQQCQTDGTWSGSNTTCVCVAPTPPANGTITLENNNLLAVYTCDIYFSMMRDS
ERTCQSDGSGWTGSSPTCVQCAPLISPFNGSLFTDTNSTHTISTFVCSLGTSMQGEATSVCMNDAT
WSSTEPICVTCPSLLDPSSGNVTLSTDGMTTTAEYACASGYEVSGQSVSTCLADGSWSYTTPECLCED
PSSFANGTVTSDGRDANFQCNVGYSLLGTSGIACNNDGTGWSEQFPECIKCNDLNTPSGGNVVFTS
TGVTTVAGFLCDVGATLNGSTSLTCHVDGSWNGTAPSCVSCEVLASPANGEYVLSTDGTTTTIQLQC
NDGYEAAGTTNIECGTDGQWIYSKQSTCDKKEEVANSLLGALIGVSIASLLIITVLSILLARFVYLYLQRK
SGGDKYTTNVTLFDSAGKFKYSDDEFPFNPVKLTRSAAPVNHERPVFDISGPFSTSPAPQGFENHVTG
HEAVPESPLPIETHRSGNSIISFRRSTTTLTDKSLSSVTAITIESAKETPAITAPLAKNINGLPPIKNGTTPLQ

EPELKPRRLSPIPKHMQRRGKLSPSSPQINKVFKESKSDAEGRQSPFEGRSPIVMSHPLNVTDFESQSL
DSVSMVGE
>Mm_P_selectin_22
MDFKTDFILILFILDLEQHLILQVNGACTWPAEFVSTRWHDSSRGILTFGTESMQGWTFTPTTGTVVSS
WDCVSVSTFNSDGRMILITSQPFSLFSTNYYAYLCITVTKVTVSSYRYYINHGIQGNVNNERVFIYPVST
LTDYGTICSDSGASGGEYQMLIKDGNESAAKIDCPWHFLGRYGYTHTVFGGSTTCDQSSSEIDMCTD
TQEIKFDSSACSTKVAYSVSDTLWCVDNIAEGDTNYVMVFNGNDAHSLIDNQSVFRFTCMAVSGDA
SSASVSPRYCQNGQTPDQYPSTASGQTTGALLAFSNSTETCPTCPSLTDPDQGSVSISTMGSVTSASY
TCEANYTLWGTSTRTCQSDTTWTGATPSCSCETPPTPSNGAVTVNSDGTVATYICTVGYTLNGLRSR
NCQTASATWEGNEPNCTECEAFTTITGQTFTLSTNGTDTSVSFVCSSGYTLSGSSSLSCSADGTWNS
QQPNCVSCATLTNPASGSVTLSSSGGTTTATYTCSGGYTLQGDATRTCQLDGTWNNVQPTCVCNQ
PNSPSNGAVVANGETATYTCNVGFTLNGDVTRTCQTDGTGWSSTNPQCSACNALSSVTGGSYTTS
TTGTVTSAKYSCQVGYSIDGVTTITCQSDGSWSSSAPSCILCPTLTNPSSGTVTLSTSGTKTSATYTCAS
AYILNGNSIRLCNSDGTWSLVEPTCTCQSPSGPLNGLVTDDGATATYTCDQGYSLSGSSTRTCNTDG
TGWSGSDSTCNICGTLSTPTGGTVIITTDGVNTRAAFTCYNGYTLNGVSPITCRSDGSWDFTAPTCSK
CPDLSDPDSGTLTMTTSGSVTTATFTCQSGYYLSGAFTLTCGSDGLWSSDSPTC
>Mm_P_selectin_23
MDFKTDFILILFILDLEQHLILQVNGACTWPAEFVSTRWHDSSRGILTFGTESMQGWTFTPTTGTVVSS
WDCVSVSTFNSDGRMILITSQPFSLFSTNYYAYLCITVTKVTVSSYRYYINHGIQGNVNNERVFIYPVST
LTDYGTICSDSGASGGEYQMLIKDGNESAAKIDCPWHFLGRYGYTHTVFGGSTTCDQSSSEIDMCTD
TQEIKFDSSACSTKVAYSVSDTLWCVDNIAEGDTNYVMVFNGNDAHSLIDNQSVFRFTCMAVSGDA
SSASVSPRYCQNGQTPDQYPSTASGQTTGALLAFSNSTETCPTCPSLTDPDQGSVSISTMGSVTSASY
TCEANYTLWGTSTRTCQSDTTWTGATPSCSCETPPTPSNGAVTVNSDGTVATYICTVGYTLNGLRSR
NCQTASATWEGNEPNCTECEAFTTITGQTFTLSTNGTDTSVSFVCSSGYTLSGSSSLSCSADGTWNS
QQPNCVSCATLTNPASGSVTLSSSGGTTTATYTCSGGYTLQGDATRTCQLDGTWNNVQPTCVCNQ
PNSPSNGAVVANGETATYTCNVGFTLNGDVTRTCQTDGTGWSSTNPQCFLCPTLTNPSSGTVTLST
SGTKTSATYTCASAYILNGNSIRLCNSDGTWSLVEPTCTCQSPSGPLNGLVTDDGATATYTCDQGYSL
SGSSTRTCNTDGTGWSGSDSTCNICGTLSTPTGGTVIITTDGVNTRAAFTCYNGYTLNGVSPITCRSD
GSWDFTAPTCSKCPDLSDPDSGTLTMTTSGSVTTATFTCQSGYYLSGAFTLTCGSDGLWSSDSPTC
>Mm_P_selectin_24
MGCISPPSLPNGTLLISDDGESAFYACVTGFSLNGDNERTCKLDGTGWNGTQPVCEQCQSIPSALNQ
EYNLSTDGTSTTVEFTCNTGSSISGEATLTCLPDGSWSDDFPLCVECPSLTSDSGGTLSYTSNGTVTAA
VFQCDEGSALVGSVQTFCETDGTWSASEPTCVQCPELASPLNGEVVMVVNGSTTWATFPCNDGYS
VAGTPQIYCTTAGTWDSAIPVCECDTFTQPLNGNIVIAANSSVATVTCDEGFALSGETTLYCSDSSGSS
DTSSIPSCVSCESLSTSPGSSLELSTDGVNTIATYSCDSGHVMNGERVLTCRSDGSWNHEPPMCSVCD
PLDSPLGGTVQLSSDGENTTAVYTCVTGFSLVGQLELTCKSDGSWDFVQPTCECDLPPSVDNGAVTV
SSDKLTATYSCDVGYTLSGPDVRHCRTNGSWWDGTDPVCSECAALDAGVGMNVTLFGDGKATSAI
FRCENGYSLSHTNNVTCLDDGSWNTAAPTCVQCASLNTTTALSYSTASDGLTTTATFTCATGYGMS
GPQSVTCVRNGSWSMTQPTCFKSSDQTGQQGSGGDGGGSGATIAFGVLFGIATIVAIILAVVAWRL
WRKSKSIDGPKSTPFVPGQVQFNPLFSEIDRDKVTPSTAPVGHERHVTDTGTRRFFSRTTWPS
>Mm_P_selectin_25
MASFTCDQDYSLYGSSILACTSSGLWNESSPECVCSTFIQPDNGDLVISENGTKATYSCAAGFTLSGDL
EQTCGGQSSSGGALEASTCTTCDPLSTSPGATFELSTDGSETVAIYQCQAGYSMIGTSNLTCRTDGTW
DVSPPGCVSCPALADPANGQVNQSTTGQVSVAKFSCVDGYYLDGNEILTCLESGTWDNVSPTCICD

PPEDLMNGAYILSNDGMSVTYTCDVNYTLNGTTIRTCSNNGNGWDGVTPTCYKCDDLSEHENGTIS
TETDGVSTSTTFTCAEGHSLSGSSVLQCLPDGTWDAGQPSCKKCEDIETPNNGSLTFTSNGTSTLAEY
SCDVGFSVSGSVEMYCAGRRHLDWRYSILWNKISCPMIETLEYGDVTVTSNGSVTIATYSCVQDYSLL
GDAVRTCTSSGAWESTTPTCVCSTFVEPSDGDIVISENGTQATYSCGTGFTLSGDVHQTCGVVTSVG
DTVLTPPTCIMCESISTSTGVTFELSTDGSQTIATFQCPTGYTMVGTSNLTCRTDGSWDISPPTCETCAT
LATVSDGSVSFVTDGEQTQAVFTCDPGSTLSGPSTLSCRTDGTWDIAQPDCVTCPSLNDPVNGQVN
VSTSGQVSGCAVYLRRRILYIRK
>Mm_P_selectin_26
MDHRRTSVFTCDAGYSLNIEEDIATCSSDGTWDQTSLDCVQCESLTAIENGLINITSGGSSSFAKYTCV
AGYTLLSDGQRNCLTNGSWDGSEPLCVCNSPTAPANGHVSVSGDFMTATYTCDTGYTLNGVSQRY
CGNDGAGWNDQKPTCELCETLSSVTGGSVELWTDGESTFANFTCTTGFSMDGEAQIQCRSDGTW
NFNVPSCTTCDSLSAVSGGTYSYSTDGETTSVTYECDVGYSMSGSPGASCSSVGSWSTAEPECVACP
LLVSPDSGFVSMTTDGSVSTAVFSCTADYHVVGNVISVCGEDGSWNSTEPVCVCDTPTSVSHGSITIS
TDGSVAAYSCDAGYTLEGNVERVCASDGSGWNGTTPECVTCDSLISPINGSLDITTDGVYTSATVSCD
VGFTSAGDTSLTCQSDGTFDSTVPSCETCEQFPVISSGSYSTATNGTTSYVTAQCVTGYYLDGEAVVT
CLDDGTWSHTPACLCEDPETPTNGATTSSGSLAMFACDTGFSLLGASVSVCRPDGGGWLIASPSCV
QCNTLNNISQGEGQISTDGSQTFITYSCPVGSTLDGDVENFCYSDGTWSSAVPSCVTCEALSNPASG
TVQLDSDNSTTLAVYACASGYELVGDDIRTCLSNGTWTLEKPTCVCIDPDPPTHGNVDVTADKTLAT
YTCDVGYTLNGESSRTCQTDGSAWTETGPSCSLCDTITSPSGGAVSLSTDGEVTIASFTCSAGYTMDG
GSIASCRDDGSWNTSVPTCVLCDSVSVPQNGAYTFVTNGETSTAVFTCDTGFTLQGTSDLTCAVSGR
YSDSEPTCLQCDTLVTPDSGVLTTTTNGTVTVATFACDTGYYLNGAAHLTCGTDGTWDGSEPECRC
DPPPTIANGFYNISTDESVVTYSCLRRIYYDRRKKSNLSK
>Mm_P_selectin_27
MSRKAAISDTVYQASSFIKRLAAEKCTITYTADKNIGLYPAALQIEDYPSILSTTALSSIPLQFIARIYAGNN
CDDVPEFIQPTPEDNACFALDVGDTYNVTIKARGQRNFHRITTISAIGMVRSSEIKSKDSSMYYVELNV
TWTPTISDSGKDHSLCFYATDNSSITSTMRCIKLPVSGLVEGYVSCGALSVPVNDSSFWKIEVDCGELTI
ANGIVLAPNGTDYPHSVTYMCNIGHDLVGTENRTCTANGTWTESEPYCKPRDCGNFPELVNGYVST
NSTIYNTTVNFFCITGYEVSGNASMFCDHTPAWSSPPPVCVIIDCGLLGPPVNGNIDMTNTTVGNTV
TYSCGIGFILHGDSKRECLHSGFWNGSNPTCKVADCGELPPIDGGVYLFNGTTYGEILTVECNVGYDL
HGSSLRQCQSSGNWSGSTATCTIKDCGPLQNPVDGSVLALATSYGTSVTYSCKTGYSLTGGDTRVC
QADGFWSGTPPTCQIKDCGNLTAPLFGEVTFNSTFYKDQASFICYTGYELIGDDVVTCQSDNTWSGS
DHVCVLKDCGFLDSPENGIATATAATYGTIETFTCNEGFELIGDDILVCTANGTWNGTVPICLIKDCGD
LLPPSNGGYFC
>Mm_P_selectin_28
MRFLLFLLFLPILFKYIDGQCTWPSEFASSTWTDNARGSLEFTQTTMSGWSFTKDTQVINEWQCIDVD
QFESDGYLVMRSVNTFIQFVTSYYYAYLCLKLTPVTDVSYRYYVMEPQQPELDWDRYLVTTDGTRSTV
ATVCFDAYGASGAEYNVMIKSGQEGSATISCPWVFLGSFDYTHTSTSGTTSCSAGGNTWDMCTDTS
SMIFDLSTCPVDVPYSGTSTVQCVDTVPVGSDNFITVYNGESFDGVNTFQFACFAVNDDLTTVSVAP
GYCRHGQTSSSYPVDTSQSDPARSDIGALISITSTYETCYTCPVLDSPDGGSVSIETNGATTTAYYTCDA
GYALYGDPERICQGNNTWTGQPPTCKCEAPSDPDHGSASVSADGSTVTYTCDIGYVLTGNQYRWC
QTDGNAWNGTDPICETCTVLGDLTNGNVTLMQESYTTAAIFACDVGYSLLGANNVTCQTDGTWSD
VMPTCVACQSLTSPSLGNITLSTDGMNTYAQFACTDGYKVLGDDLLSCLTNSSWDSVEPTCICEDPE
APAFGQVTVDDTGMLASYTCDVGYTMDGERTRDCQLDSTSWTGDVPSCVTCTDVPSSGANITLST
NGTQTTASFSCLPGYTMIGEVTSECSTAGIWTPTAATCVSCDALVSPASGNVSVTSNGTVSVAEYTCN

SGYTLSGAATRTCQEDGSWDGQAAQCDCEELTDPVNGNLTMIGQTAYYACAVNYTLSGEFQLVCQ
EDGTGWSNTPPTCAECQSLQDINDGSITFTSVSGLNTTAQHACDIGFTLVGFTSRTCNADGTWDGS
APYCQLCDVIQSPNDGAVTLTTNGSQTIATFTCDVGYTVNDVTIVTSECDQSGVWESTNVECISCEQ
LTNISSGATLVSSNGTFTTASYSCVSGYDLVGVSTRTCLSTGTWSDTEPRCKCETPDSVPNGSVAVSFD
GMKAFYTCDSGYSMNGVSNITCQEDGSGWDYASPSCVHCDDLTDPTDGLVTVTTDGSNSIATYTC
DVGYSLDGTGTSVSTCNSDGAWADQAPSCVKCETLTTPDSSTMTLSFNETSTQALFSCVSGYYLDG
QSLLSCGQNGTWDYESPVCVCEIPTSPANGSISVSGDGFTITYSCDIGHSLNGDATRTCQNAGQSWS
GSAPSCVSCDTVPSLLDGNIEYSSNGTVTELSYSCNVGYTLYSVTRETSATCDDDGTWNTAIVSCVEC
DTLSSPSSGSLLLSTDGLTTSAYFDCATDYYMVGENSSVCETSGVWTTSAPSCLCVLPTVPTNGAMTL
SDDNLTLTLACDVGYIISGSALRTCSSDGTGWSGVQPTCDSCDTLSTPTNGQIELNTDGTVTTAIFTCD
SGSSMSGNPTLTCNTDGSWD
>Mm_P_selectin_29
MTVYYSCNSGYTLSGQDSRLCSIDGTGWAGFQPSCVFCPTVSAPTGGSVTVATDGTTSRAYFTCNLE
YTMDGSPTTTCTVNGTWDTVAPTCVQCSGLSDPDSGSVTLTTDGEQTIASYDCVTGYQISGVTPLTC
QTDGQWSYSEPTCVCTPPLTPTDGLLEISNDGMTANFTCTAGYTLKGVSTRTCQSNGAGWNEAQPT
CETCDALSNPTGGTVSLTTSGTVTVADITCQTGYTINGASTLACRTDGTWDISVPTCGVCSALSSPSSG
TVDLYSENSVTKANFSCESRYSLSGASILTCRTDGSWDFSRTSLCQVHRFTNCECRNY
>Mm_P_selectin_30
MTTKVIFIAVYMCLFQRITSVGGTRTTVGRNVTADLTQYDWRNKQTIDCGQPHDPTNGRVYLNGNV
TTYNATATYTYNDGYTRIGEPISRCDSSGSWSKPPVCKIKDCGNLTDPSNGKVDLPNGTTFLSVGKFR
CNVGHTLHGDETAVCTIKDKWSDHYVTCTVNDCHRPNPPGNGSVDTTNGTTYESVAAFACNTGFH
LEGASKSVCQANGSWSAPSRNCVVKDVRDCGRLPNPANGAIKYVRNMSKYVWSSATYSCQPGFR
MIGPEVIYCDAQGNWSSYTTYCQIKDCGNLADPLYGKTDMSNGTTYLSVVKYSCNIGYTFNGDNAT
TCTATGKWSDHYVNCTINDCLSPVNPGNGSVDTTKGTTFGSIVIFSCNTGFDLEGESSAICLANSSWN
VSSPNCKIKDCGALPDPEHGDIMYSRNLTSFGSSATYTCQPGFKSVGSEVILCDASGHWSSNKTHCEI
KNCGNLTDPPNGTVDLSNSTTYQSVGKYSCNIGYTLQGDNSTICTETGKWKTHYVECKINDCLSPGH
PGNGSVDTTCGTTFGSIATFSCNIGYDLKGERHAICQANSSWNITLPICYIKDCGKPNQTANGKYSLD
ARQLTVFNHTATYRCNSGYETADKTLITCNSSGKWSDMPPKCKDIDECARYTARCHRRAQCTNTNG
SYVCECDDGFRDISMNGEIGIECQDINECANNNTNPCNRKGSQCINYSGGFLCLCDDGWTDNNCS
TDINECANDSTNPCNRKGSQCINYPGGFLCLCDDGWTDNNCSTENVIIDLETVVTRFRGDDQLIDAK
PIHNRFPYFGEEYNSYRPNMNGFLTLGYQPLY
>Mm_P_selectin_31
MTTKVIFIAVYMCLFQRITSVGGTRTTVGRNADLTQYDWRNKQTIDCGQPHDPTNGRVYLNGNVTT
YNATATYTYNDGYTRIGEPISRCDSSGSWSKPPVCKIKDCGNLTDPSNGKVDLPNGTTFLSVGKFRCN
VGHTLHGDETAVCTIKDKWSDHYVTCTVNDCHRPNPPGNGSVDTTNGTTYESVAAFACNTGFHLE
GASKSVCQANGSWSAPSRNCVVKDVRDCGRLPNPANGAIKYVRNMSKYVWSSATYSCQPGFRMI
GPEVIYCDAQGNWSSYTTYCQIKDCGNLADPLYGKTDMSNGTTYLSVVKYSCNIGYTFNGDNATTC
TATGKWSDHYVNCTINDCLSPVNPGNGSVDTTKGTTFGSIVIFSCNTGFDLEGESSAICLANSSWNVS
SPNCKIKDCGALPDPEHGDIMYSRNLTSFGSSATYTCQPGFKSVGSEVILCDASGHWSSNKTHCEIKN
CGNLTDPPNGTVDLSNSTTYQSVGKYSCNIGYTLQGDNSTICTETGKWKTHYVECKINDCLSPGHPG
NGSVDTTCGTTFGSIATFSCNIGYDLKGERHAICQANSSWNITLPICYIKDCGKPNQTANGKYSLDAR
QLTVFNHTATYRCNSGYETADKTLITCNSSGKWSDMPPKCKDIDECARYTARCHRRAQCTNTNGSY
VCECDDGFRDISMNGEIGIECQDINECANNNTNPCNRKGSQCINYSGGFLCLCDDGWTDNNCSTDI
NECANDSTNPCNRKGSQCINYPGGFLCLCDDGWTDNNCSTENVIIDLETVVTRFRGDDQLIDAKPIH

NRFPYFGEEYNSYRPNMNGFLTLGYQPLY
>Mm_P_selectin_32
MTTKVIFIAVYMCLFQRITSVGGTRTTVGRNVTADLTQYDWRNKQTIDCGQPHDPTNGRVYLNGNV
TTYNATATYTYNDGYTRIGEPISRCDSSGSWSKPPVCKIKDCGNLTDPSNGKVDLPNGTTFLSVGKFR
CNVGHTLHGDETAVCTIKDKWSDHYVTCTVNDCHRPNPPGNGSVDTTNGTTYESVAAFACNTGFH
LEGASKSVCQANGSWSAPSRNCVVKDVRDCGRLPNPANGAIKYVRNMSKYVWSSATYSCQPGFR
MIGPEVIYCDAQGNWSSYTTYCQIKDCGNLADPLYGKTDMSNGTTYLSVVKYSCNIGYTFNGDNAT
TCTATGKWSDHYVNCTINDCLSPVNPGNGSVDTTKGTTFGSIVIFSCNTGFDLEGESSAICLANSSWN
VSSPNCKIKDCGALPDPEHGDIMYSRNLTSFGSSATYTCQPGFKSVGSEVILCDASGHWSSNKTHCEI
KNCGNLTDPPNGTVDLSNSTTYQSVGKYSCNIGYTLQGDNSTICTETGKWKTHYVECKINDCLSPGH
PGNGSVDTTCGTTFGSIATFSCNIGYDLKGERHAICQANSSWNITLPICYIKDCGKPNQTANGKYSLD
ARQLTVFNHTATYRCNSGYETADKTLITCNSSGKWSDMPPKCKDIDECARYTARCHRRAQCTNTNG
SYVCECDDGFRDISMNGEIGIECQDINECANNNTNPCNRKGSQCINYSGGFLCLCDDGWTDNNCS
TDINECANDSTNPCNRKGSQCINYPGGFLCLCDDGWTDNNCSTENVIIDLETVVTRFRGDDQLIDAK
PIHNRFPYFGEEYNSYRLWTK
>Mm_P_selectin_33
MTTKVIFIAVYMCLFQRITSVGGTRTTVGRNDCGQPHDPTNGRVYLNGNVTTYNATATYTYNDGYT
RIGEPISRCDSSGSWSKPPVCKIKDCGNLTDPSNGKVDLPNGTTFLSVGKFRCNVGHTLHGDETAVCT
IKDKWSDHYVTCTVNDCHRPNPPGNGSVDTTNGTTYESVAAFACNTGFHLEGASKSVCQANGSWS
APSRNCVVKDVRDCGRLPNPANGAIKYVRNMSKYVWSSATYSCQPGFRMIGPEVIYCDAQGNWSS
YTTYCQIKDCGNLADPLYGKTDMSNGTTYLSVVKYSCNIGYTFNGDNATTCTATGKWSDHYVNCTI
NDCLSPVNPGNGSVDTTKGTTFGSIVIFSCNTGFDLEGESSAICLANSSWNVSSPNCKIKDCGALPDP
EHGDIMYSRNLTSFGSSATYTCQPGFKSVGSEVILCDASGHWSSNKTHCEIKNCGNLTDPPNGTVDL
SNSTTYQSVGKYSCNIGYTLQGDNSTICTETGKWKTHYVECKINDCLSPGHPGNGSVDTTCGTTFGSI
ATFSCNIGYDLKGERHAICQANSSWNITLPICYIKDCGKPNQTANGKYSLDARQLTVFNHTATYRCNS
GYETADKTLITCNSSGKWSDMPPKCKDIDECARYTARCHRRAQCTNTNGSYVCECDDGFRDISMNG
EIGIECQDINECANNNTNPCNRKGSQCINYSGGFLCLCDDGWTDNNCSTDINECANDSTNPCNRK
GSQCINYPGGFLCLCDDGWTDNNCSTENVIIDLETVVTRFRGDDQLIDAKPIHNRFPYFGEEYNSYRP
NMNGFLTLGYQPLY
>Mm_P_selectin_34
MTTKVIFIAVYMCLFQRITSVGGTRTTVGRNVTADLTQYDWRNKQTIDCGQPHDPTNGRVYLNGNV
TTYNATATYTYNDGYTRIGEPISRCDSSGSWSKPPVCKIKDCGNLTDPSNGKVDLPNGTTFLSVGKFR
CNVGHTLHGDETAVCTIKDKWSDHYVTCTVNDCHRPNPPGNGSVDTTNGTTYESVAAFACNTGFH
LEGASKSVCQANGSWSAPSRNCVVKDVRDCGRLPNPANGAIKYVRNMSKYVWSSATYSCQPGFR
MIGPEVIYCDAQGNWSSYTTYCQIKDCGNLADPLYGKTDMSNGTTYLSVVKYSCNIGYTFNGDNAT
TCTATGKWSDHYVNCTINDCLSPVNPGNGSVDTTKGTTFGSIVIFSCNTGFDLEGESSAICLANSSWN
VSSPNCKIKDCGALPDPEHGDIMYSRNLTSFGSSATYTCQPGFKSVGSEVILCDASGHWSSNKTHCEI
KNCGNLTDPPNGTVDLSNSTTYQSVGKYSCNIGYTLQGDNSTICTETGKWKTHYVECKINDCLSPGH
PGNGSVDTTCGTTFGSIATFSCNIGYDLKGERHAICQANSSWNITLPICYIKDCGKPNQTANGKYSLD
ARQLTVFNHTATYRCNSGYETADKTLITCNSSGKWSDMPPKCKDIDECARYTARCHRRAQCTNTNG
SYVCECDDGFRDISMNGEIGIECQDINECANDSTNPCNRKGSQCINYPGGFLCLCDDGWTDNNCST
ENVIIDLETVVTRFRGDDQLIDAKPIHNRFPYFGEEYNSYRPNMNGFLTLGYQPLY
>Mm_P_selectin_35
MTTKVIFIAVYMCLFQRITSVGGTRTTVGRNVTADLTQYDWRNKQTIDCGQPHDPTNGRVYLNGNV

TTYNATATYTYNDGYTRIGEPISRCDSSGSWSKPPVCKIKDCGNLTDPSNGKVDLPNGTTFLSVGKFR
CNVGHTLHGDETAVCTIKDKWSDHYVTCTVNDCHRPNPPGNGSVDTTNGTTYESVAAFACNTGFH
LEGASKSVCQANGSWSAPSRNCVVKDVRDCGRLPNPANGAIKYVRNMSKYVWSSATYSCQPGFR
MIGPEVIYCDAQGNWSSYTTYCQIKDCGNLADPLYGKTDMSNGTTYLSVVKYSCNIGYTFNGDNAT
TCTATGKWSDHYVNCTINDCLSPVNPGNGSVDTTKGTTFGSIVIFSCNTGFDLEGESSAICLANSSWN
VSSPNCKIKDCGALPDPEHGDIMYSRNLTSFGSSATYTCQPGFKSVGSEVILCDASGHWSSNKTHCEI
KNCGNLTDPPNGTVDLSNSTTYQSVGKYSCNIGYTLQGDNSTICTETGKWKTHYVECKINDCLSPGH
PGNGSVDTTCGTTFGSIATFSCNIGYDLKGERHAICQANSSWNITLPICYIKDCGKPNQTANGKYSLD
ARQLTVFNHTATYRCNSGYETADKTLITCNSSGKWSDMPPKCKDIDECARYTARCHRRAQCTNTNG
SYVCECDDGFRDISMNGEIGIECQDINECANNNTNPCNRKGSQCINYSGGFLCLCDDGWTDNNCS
TENVIIDLETVVTRFRGDDQLIDAKPIHNRFPYFGEEYNSYRPNMNGFLTLGYQPLY
>Mm_P_selectin_36
MTTKVIFIAVYMCLFQRITSVGGTRTTVGRNVTADLTQYDWRNKQTIDCGQPHDPTNGRVYLNGNV
TTYNATATYTYNDGYTRIGEPISRCDSSGSWSKPPVCKIKDCGNLTDPSNGKVDLPNGTTFLSVGKFR
CNVGHTLHGDETAVCTIKDKWSDHYVTCTVNDCHRPNPPGNGSVDTTNGTTYESVAAFACNTGFH
LEGASKSVCQANGSWSAPSRNCVVKDVRDCGRLPNPANGAIKYVRNMNCGNLADPLYGKTDMSN
GTTYLSVVKYSCNIGYTFNGDNATTCTATGKWSDHYVNCTINDCLSPVNPGNGSVDTTKGTTFGSIVI
FSCNTGFDLEGESSAICLANSSWNVSSPNCKIKDCGALPDPEHGDIMYSRNLTSFGSSATYTCQPGFK
SVGSEVILCDASGHWSSNKTHCEIKNCGNLTDPPNGTVDLSNSTTYQSVGKYSCNIGYTLQGDNSTI
CTETGKWKTHYVECKINDCLSPGHPGNGSVDTTCGTTFGSIATFSCNIGYDLKGERHAICQANSSWNI
TLPICYIKDCGKPNQTANGKYSLDARQLTVFNHTATYRCNSGYETADKTLITCNSSGKWSDMPPKCK
DIDECARYTARCHRRAQCTNTNGSYVCECDDGFRDISMNGEIGIECQDINECANNNTNPCNRKGSQ
CINYSGGFLCLCDDGWTDNNCSTDINECANDSTNPCNRKGSQCINYPGGFLCLCDDGWTDNNCST
ENVIIDLETVVTRFRGDDQLIDAKPIHNRFPYFGEEYNSYRPNMNGFLTLGYQPLY
>Mm_P_selectin_37
MTTKVIFIAVYMCLFQRITSVGGTRTTVGRNVTADLTQYDWRNKQTIDCGQPHDPTNGRVYLNGNV
TTYNATATYTYNDGYTRIGEPISRCDSSGSWSKPPVCKIKDCGNLTDPSNGKVDLPNGTTFLSVGKFR
CNVGHTLHGDETAVCTIKDKWSDHYVTCTVNDCHRPNPPGNGSVDTTNGTTYESVAAFACNTGFH
LEGASKSVCQANGSWSAPSRNCVVKDVRDCGRLPNPANGAIKYVRNMSKYVWSSATYSCQPGFR
MIGPEVIYCDAQGNWSSYTTYCQIKDCGNLADPLYGKTDMSNGTTYLSVVKYSCNIGYTFNGDNAT
TCTATGKWSDHYVNCTINDCLSPVNPGNGSVDTTKGTTFGSIVIFSCNTGFDLEGESSAICLANSSWN
VSSPNCKIKDCGALPDPEHGDIMYSRNLTSFGSSATYTCQPGFKSVGSEVILCDASGHWSSNKTHCEI
KNCGNLTDPPNGTVDLSNSTTYQSVGKYSCNIGYTLQGDNSTICTETGKWKTHYVECKINDCLSPGH
PGNGSVDTTCGTTFGSIATFSCNIGYDLKGERHAICQANSSWNITLPICYIKDCGKPNQTANGKYSLD
ARQLTVFNHTATYRCNSGYETADKTLITCNSSGKWSDMPPKCKDIDECARYTARCHRRAQCTNTNG
SYVCECDDGFRDISMNGEIGIECQDINECANNNTNPCNRKGSQCINYSGGFLCLCDDGWTDNNCS
TDINECANDSTNPCNRKGSQCINYPGGFLCLCDDGWTDNNCSTECNNASDTNF
>Mm_P_selectin_38
MTTKVIFIAVYMCLFQRITSVGGTRTTVGRNVTADLTQYDWRNKQTIDCGQPHDPTNGRVYLNGNV
TTYNATATYTYNDGYTRIGEPISRCDSSGSWSKPPVCKIKDCGNLTDPSNGKVDLPNGTTFLSVGKFR
CNVGHTLHGDETAVCTIKDKWSDHYVTCTVNDCHRPNPPGNGSVDTTNGTTYESVAAFACNTGFH
LEGASKSVCQANGSWSAPSRNCVVKDVRDCGRLPNPANGAIKYVRNMSKYVWSSATYSCQPGFR
MIGPEVIYCDAQGNWSSYTTYCQIKDCGNLADPLYGKTDMSNGTTYLSVVKYSCNIGYTFNGDNAT
TCTATGKWSDHYVNCTINDCLSPVNPGNGSVDTTKGTTFGSIVIFSCNTGFDLEGESSAICLANSSWN

VSSPNCKIKDCGALPDPEHGDIMYSRNLTSFGSSATYTCQPGFKSVGSEVILCDASGHWSSNKTHCEI
KNCGNLTDPPNGTVDLSNSTTYQSVGKYSCNIGYTLQGDNSTICTETGKWKTHYVECKINDCLSPGH
PGNGSVDTTCGTTFGSIATFSCNIGYDLKGERHAICQANSSWNITLPICYIKDCGKPNQTANGKYSLD
ARQLTVFNHTATYRCNSGYETADKTLITCNSSGKWSDMPPKCKDIDECARYTARCHRRAQCTNTNG
SYVCECDDGFRDISMNGEIGIECQDINECANNNTNPCNRKGSQCINYSGGFLCLCDDGWTDNNCS
TECNNASDTNF
>Mm_P_selectin_39
MAGCQLPNTDSTLTQFMKGNGTIALYKQQNTVQYRCRDGYEFSNTSGTNSTKTLTCGDDGHFTTLE
QCLAKDCGPVGEISNTETITLSPTNDTTFGSKAVVSCVHGYVVTGTENKENQFNMSCSKDGTWQPIE
DCIRIDCHAVSNIGIDHAGNVRLHSDTKYNSTADIDCDEGYYNVKDTQTTGISTTTVTCSEHGTWVNI
PTCVRKDCGKLELLNISNAKLRLKFKDTKYGSEAAISCDTGYTDTNKPQTEDKSATMIKCYANGTWAN
LPECVRKDCGNVTTVATNGHVDQTNGTTYLSVSTFHCDIGYTLYGDNTTVCTEEGKWGEYNVTCIIN
DCSEPTSPTNGIVTPINATTYRSIANYSCNEGFILNGTKITTCEANKTWSGPVPTCVKKDCGTPSVPTN
GKVDTSDDTIYQSVATFSCDTGYTLNGVNSTVCQSSGNWSEHNLKCVINDCGNLTAPGNGAVNFS
NGTTYRSLAFYYCNTGFDLDGENTTFCQANRTWNAKIPKCVIRDCGDPPKAPPNGTLASQGTTYQS
VVSYSCNAGFDLEGNASAVCQANKTWTTGPTNCRIKDCGKPNDTAHGTVLLETGHVFKDNVTYKC
NEGYETSDATFITCNSSGEWNRPPPMCKDINECDRYTANCHRRADCNNTDGSYWCKCQSGFTDIS
GDKGTNCEDIDECSSASTNNCNKDGSQCINYPGGFLCLCDDGWTADNCSIDIDECETTGCGLRANC
SNYDGGYNCTCISGYPKGDPKIHCYENVIIDLETRVDPFRGNDELIDAKPIHYRFPYYGYEYNSYRPNM
NGFLTLGYQPVYEHYGPETPDDWKKYSRGKTVIAPFWTDIDSTNLTGGLYVHLFENYTNYGPNDSQH
KDLSRLGNIFTEYYNLTDFIPRVAVVATWINVTQSSYIVPSRLIRTHNATMQVILISDGIYSYVMFNYDH
EQWSFEIDEKIPSSAGFSQSDNTGYIIATSKNFTQLNNDTNVNEGTNQIDGRWIYNVSSNDINRNAVL
RNESECLEFSRNKTIKDWITKQRELSYPCPCSEQQMQLDYSYRKVDPMYENKQTKTVCYEAWFFNN
DGVKQTCCYSYGALKKENIGGGFATFEHDKYNTTQYFYKCCDAKTDRHLCHLFYEMNPPDDCSKFQ
PADEPIGRYRGSLIGYMRGRTDRI
>Mm_P_selectin_40
MMYDLSPCSLTHSFDHYVGITVEDCILACRRRKICAAVNYNRYNKLCALRKSSEIELLGSEPEWCYDIVI
SNETAVLDGPCKDNPCNETSICLNSTKFPFFKCRLEYCLPEQEVANAAFRNKVLSLVGDRNELVCDKG
FTPFGKQSLTCLKGGNWSTTDLQCLKTCPSIPLKSTAVVSSWSTYRFVVNTTASFTCKEGFYNLTDTVI
VCDKFGTWSEFECVPFCRQADIVPIKNGSPKPGDNYTIYTQGEYVCNVDFYLSSGSKFVSCDVKGTWS
EPEVTCFQFCKQINIPTVSNGHPLHGTLYNHTTTARYECDPGFYLSDGSPEVACNTDGTWSTPKIECY
PFCQQPVIANGFLQNPASEPIKKDVSVVYSCNYGYNAYPHVTNPTAVCQKTGSWTYSAACNKNCG
DAPNYTWEKIIGYSSGPQYTTGTLVYYKCVEVGLHFSAGGGDKKTCNSNGEWSGEVHCCFACFDYID
GECRWNC
>Mm_P_selectin_41
MCNEGFVLDGPNITICQPNKTWSGPVPTCLIKNCSGLPNPKNGAITYSRNLTSYGSNATYTCHPGYNI
TGPEIIQCESSGNWSSYETHREIKNCGNLINPPDGTADMSNGTTYQSVGKYRCNIGYTLYGNSSTVCT
ETGHWKKHSVECKINDCGYPVQPGNGSIDASNGTTFESLVTFSCNTGYDLKGERYTSCQANGSWNI
TSPTCNIKGI
>Mm_P_selectin_42
MHTDIAPEHIAFAELIAYIEQFENVSVDTNHIFKLSDLVKLYHDRIIQWLWPDVYGESKYVVMMGGLHI
EMAFLKVLGDWLFDSGWITAITTAGVTTAGRADSIRKEQQWSDVMDNADIHPQFAYWNKTMNMEI
LFLQFMKSQRQANFDMYVECLGKIVPWMFAMDHIHYARWLTVHTQDLLLLQERSPDVHEEFKRGH
FVTHKTKHRFSGLAHDQIHEQQNAIIKGDGGFVGLTENPDTLRRWMVAAPETTRILTEASNCAINQD

DTKHHEETQTVQKRFHSHVRQVVETIEDMGNPFEEDTSDLYTLDTKVVMSSEVIASVKTAESVGKAKY
KSMIVERLSTDSKSFYDTIKKNSLPLFRNGSSKKTVKVSKMKSMKSGIQLFSRMYISCQIHVHQTSPISN
PAPMRKQIIVCFCMQQMQFKQGYKRVMLHVTDTDVLVLSISTICHFDNCELWLAFGHSKHFRYIPAH
SIANRLGKDVSLGLPFFHALSGCDTVSAFAGIGKKTAFTLYMALPHLIPVFKRLSSAPRIVIDEDFSLLERY
IVLLYSKTSTCQSVNEARKYLFSQGNRQIENIPPTRCALIQHVRRAAYQAGHVWGQALIANPELPSPA
DWGWAKSNGTWVPFWSTNPEAAKSCRELVKCSCKNTCTDCGMLVNLNILNASDRRLLNNETTFQS
TANISCDDGYRDEAQKKTFGVSSKIITCSKSGEWLTLPKCIKKDCGMLVYLNITNASDRRLLNNKTTFKS
TANISCDEGYKDEAQKKTFGISSKIITCSKSGEWLTLPKCIKKGSKKDLVLLNALLDVYLLLVEEISFEAFHS
HVNMSGLLINYRLRNVGKSELRNVGNLNILNASDRRLLNNETTFQSTANISCDDGYRDEAQKKTFGV
SSKIITCSKSGEWLTLPKCIKKDCGMLVYLNITNASDRRLLNNKTTFKSTANISCDEGYKDEAQKKTFGIS
SKIITCSKSGEWLTLPKCIKKDCGMLENLSISNASSRNLLNNETTYLSTANISCDEGYRDEAQNQTSRISS
KIITCSKSGEWLTLPKCIKKDCGSLDDLNISNAESNLLSNDTKYKSNATISCDPGYTDQNNHQTVGISTT
WITCSGNGTWANVPKCVRKDCGDINNISIKNAFKRHLHNDTKYNSKADVDCDDGYYNINDIQTSGI
STTTISCSENGTWVNMPTCVRKDCGDVRNISINNASERRLHNGTKYNSIAEIDCDEGYYNINDIQTSGI
STTTISCSENGTWVNFPTCLRKDCGNVSSLTISNAKKKLLVSDTKYNSTANISCDIGYYEQNTTQTRGIS
TTEIRCGDNGLWTNLPTCIRKDCHEVSKISIDNTADRRLHSDTKYISTADIDCDEGYYNVKDNQTTGIS
TTTINCSEHGTWANMPTCVKKDCGKLDMLNVSNAKTRFLFGNTKYQSNAAINCDTGYTDQNKPQT
LGISATQIKCSANGTWENVPKCVRKDCGNIYNISINNAVNRHLHNDTKYKSTADIDCDEGYYNKMQ
GQTAGISTTTISCSEQGKWMNIPTCVRKECGDLSQLKIPNAENRDPINDTKYNSIANISCDVGYKEQN
ERQSEGITSSFIRCNENGTWANQPTCVRKDCGDVQNISIDNAANRRLHTDTKYNSTADIDCDEGYFN
ENQTQTAGISTTRINCSEHGIWVNKPNCIRKNCRSLKQLNISNAQDISEINGTEYNSIANISCDVGYKE
QNERQIEGITSTLMRCNENGTWINQPTCVRKDCGDVHNISIDNAVNRRLHNDTKYTSTADIDCDEG
YYNVKDNQKTGISTTKIYCSEYGTWTNIPTCIRKDCGSLSTLSNGNIALQQNDTSYGSNATITCRDGYT
VFGPDNIVCEKTGNWSSYITHCQVNDCGRLPPPANGNITFAQNMTTYGSNATYTCDAGYHINGSKT
IQCKASGNWSTYGISCQIKDCLTPKPVQDGFIETPNGTKFGAVAIISCNPGYTLHGDNFTKCMADGS
WDLYSVNCTLNECPKANDIDHGSVNTTLKSHVVYKCETGYTLYGQKSLFCQLNGTWSAETPKCKITD
CGNLTSPENGHVTLNNGLTTFNSSATFTCDTGYELTDKGISICNASGNWSSTPNCRIKDCGNVTDPK
NGYVDQTKGTTYLSESTFNCNIGYTLHGDNSTVCTEYSRWSAYNVTCVINDCSEPTSPTDGNVTATN
GTTYSSIANYMCNEGFVLDGPNRTICQSNKTWSGPIPTCLIKNCSGLPNPENGAITYSRNLTSFGSNAT
YTCHPGYNITGPEIIRCESSGNWSSYETHCEIKNCGNLINPPNGTVDISYGTTYQSVGKYGCNTGYTLY
GDNSTTCTETGLWKKHSVECIINDCGYPVQPGNGSVDATNGTTFESMVTFSCNIGYDLKGEKHASC
QANRSWNITSPTCNIKDCGKPNQTANGKYSVDARQLTVFNHTATYKCNSGYETADVMLITCNSSGK
WSSPPPKCNDIDECARYTARCHRRAQCTNTNGSYKCKCDDGFNDISKNGEVGIECQDINECANDNT
NPCNKKGSQCINYPGGFLCLCDDGWTDNNCSTDINECLDNTTCGVLASCENYDGGYNCTCDPEFP
KGDPKIHCYENVIIHLETVVTRFRGEDQLIDAKPIHNRFPYFGVEYNSYRLFKSRNV
>Mm_P_selectin_43
MLPKCVKKDCGKLVRLNISNASTRQLPNNKTTFGSTANISCDEGYRDEAQDQTSEISSKTIKCSKGGE
WIDLPKCVKKDCGKLVKLNISNASTRKLSNNKTTFGSTANISCDEGYRDEAQEQTSEISAKTIKCSKGGE
WIVLPKCVKKDCGKLVKLNISNASTRGLSNNKTTFGSTANISCDEGYRDEAQEQTSEISSKTIKCSKGGE
WIELPNCVKKDCGTFVNLNISNASTRQLSNNKTTFESTASISCDEGYRDEAQEQTSEISSKTIKCSKGGG
WIELPKCVKKDCGKLVKLNISNASTRQLSNNKTTFGSTANISCDEGYRDEAQEQTSETSSKTIKCSKGG
EWIELPKCVKKDNYLVCGNVHNISINNASNRRLHNATIAEIDCDEGYYNINDAQTSGISTTTIRCSENG
TWVNMPTCVRKDCGKLHQLNISNAKMRLLFTDTKYKSNAAISCDTGYTDKNKPQTKDISTSLIKCSA
NGTWAHVPTCVRKDCGNVHYISINNAANRRLHNDTKYTSTADIDCDEGYYDIKVTQTTGISTTTITCS

DHGTWVNVPTCERKECGGLSELNIANAKDRIPINGTKYNSIANISCDVGYKEQNKSQTTGITSTFIRCN
ENGTWANQPTCVRKDCGDVYNISIYNAVNRRLHYDTKYKSTAEIACDEGYYNINDPQTSEISTTTITCS
DHGTWINMPMCVRKDCGKLDMLNISNAKLRLTFGDTKYQSNATINCDSGYTDQNKPQTFAISSTWI
ECSADGTWANIPNCVRKDCGNVHNIIINNASKRRLHSDTKYNSTAEIDCDEGYHNINDILTSGISTATI
LCTENGTWMYIPTCVRKDCGQIQLNEHSNLKYSLNDTTYGSIATTVCDAGYDMHYLLGGTNGEWNI
TCDQKGMWSQLPQCTIKDCGQIQLNEHSNLKYALNDTTYGSIATIVCDAGYDMHYLLGGTNDEWN
ITCDQKGMWSQLPQCTIKDCGQIQLNEHSNLKYAPNDTTYGSIATIVCDAGYDMHYLLGGTNDEW
NITCDQNGAWSTLPQCTIKAILNTGKPEICFNCDDMSSPEYCDIVARCQKNQVCYTESYQSVYGKEF
RSGCMDEHVLRSRVALNAFIYYFFQYFGGKSWKELVKDVFKRQVGIGSNSHDLHGDA
>Mm_P_selectin_44
MSSCEYPPPSPKYGSVKITDVFKVIYQCNQGYYLSSGDHQRTCDGDYKKGTWSGQLPTCSPVFSRNC
THNEDCSSLLHSTCRSKNEPIVKRSQSVTKSSQCLCEKGHYYNVTSNSCYKDCGDPEKPTNGAVNTS
NGTQYLAVATYNCNTGYDLDGTSTTVCNADGNWTHGPPRCNIKDCGDPEKPTNGAVNTSNGTQY
LAVATYNCNTGYDLDGTSTTVCNADGNWTHGPPRCNIKDCGDPEKPSNGTVVTSYGTKYLAVATYE
CNTGYDLDGNSTTVCNADGNWSYGPPRCNIKVCRNLSSPDHGKVDHSNGTTYLSEAKYSCDTGYTL
YGVNVTTCTETGNWSEHIVNCTINVCQLPDLPVHVEFVSDVKNGTDLVYNTTLLYRCVAGYGFQSN
ATTHENKTIRCQDNSSFSHFETCKLKVCQLPDTLHEKHIEIVTDVKNKTEFTYNTTLQYTCEAGYGFER
NATTHEHKSITCQENASFSDFKECKRKDCGQLPKVDQNNRWQNVSLTNSSSDTYYQSTATAYCLEG
YRIQGKQNDIVSLTFMCQSNMTWGPVPQCEKKDCTEPGSTTHGSYSPVHGQTFFNDNVTYKCDPG
FETTEKTFITCNSSGSWSSPPPKCKDINECDLYTANCHRRADCTNTIGSYTCKCQEGFRDISVGGIGLN
CEDKNECTNASANTCNKNGSQCINYPGGFLCLCDEGWTGNTCDTDINECNTTGCGEKAYCTNYAG
GYNCTCNDGFPKGDPKIHCYVKVILDLETVVKPFRGDNQLIDAQPIKYRFPYFGDEYNSYRPNMNGFL
TLGYQPVYEDYGPETPDDWKKYSRGNTVIAPFWTDIDSTNLTGGLYVHLFEYYTNYRQNNSQQRDLS
YLGSKFADYLNLTDFIPRVALVASWINVTQSSYIEPSRLIKTHNATMQAILISDGIYSYLMFNYDHEQWS
FKIDNNIPSSAGFTRKDKTGYIIATSQNFSQLNTGTNLHPGLNGRWIYNVSSNDTNTNNVLRNESECL
AFRHNETIKNWITAKRDLSYPCPCSEQQMQLDYSYRVVDPMYKHSHTPPVCYEAWFFNNDGVKQT
CCYSYGALKKEATGGGFATFETDLYNTTEYFYKCCDENTDRHLCHLFYEMNPSDDCSRFNPADEPFD
RYRRSLFGNMRSGPDVNMYRHESKARKWSKQMEKKKYI
>Mm_P_selectin_45
MCHKWFLFVHVQDELHKVSQRTSVLIYSAENKRMKQSHLILICAICAIAFLRLASLPYIDAKYHKAQTYL
QEGDDACTYKYFCSETDVIHLAPQFESPCIPKNIQNIYYTGPGSNEITVSWIEPVAKDRNGNGSRVEQ
VQGGINGSTFAGTVSGLTHFIAYVTTDSDGLKDNYIFNFSVIVPTCTNLATVQNGRYICTNSTAVGSSC
RLLCDEGFRESGSGNATCRYESKTSKFPHWENVQHCEEISCGSLNVPDNATVTCTTNTYIYKTICKVDC
NKGFLAVGPMYTICQSDRHWSKSGKCTSQYLDGEYCSPTLCSGHGCVDVLNEELLCTCYHGWSGHI
CEIPPDYCAEGYLVFAIEKLKVLTGENEIIDSSI
>Mm_P_selectin_46
MTTANTLMMYCGDPKPLNGFINSTKTSYGTVVKVTCSWGFKLVGNSVIKCELSNSWSDSPICKLIDC
GDPTPEHGSSNATSTTNGSVVMIACDIGYNMSGNALITCNSSEMWTDYPTCEIVDCGLPSPQNAYV
TLSGNKTTYGEIAVISCLVGYRPDGSSPVSCLANGTWESWPSCEIIDCGDPTPDKGQSNDTKTTYGTVI
SITCDEGMNISGNSTIICQADGTWSDTPVCDSSDCGLLSVSHGIVNISDGTTTGSLAYVTCNQGYKLK
GSPIVACTSAGWNDSVSCIKIDCGDPTPENGKIVENDGTDYGSVAKIECNIGHELLGDNIITCQEDGM
WSDSPKCNIRDCGQPTPLPDNAYWDDSEGTTYNASIKFVCDDGFLLVGEETIVCQSTGVWSSDPPA
CSEKADIGGQCFGDEYCHTRDAVCVNSVCACKSNIHDERTNKCNTMPILPFGQDQGDLTIHSGDVC
GSKISFAPGIPLFDRLHKDLYVCSNGIISFGSKYINPTPPGDRDSVSGIEHNAIIAPFYSPIDKNTGKVFYRS

YDILNSNKKLEDDKASQKSLIVYLEKVVKIFGGIDKFETSFVLLATWDSVKTNNTDYDKTKTSTFQLVLIS
DGTTTYTLVHLRTRTDAVAT
>Mm_P_selectin_47
MEFTNLSAKPEVAEKIETLGETPLELTSSFAEGGNVSETTNMVKKERFSVFKGKLLLNRRICIIILLVIIIVLVL
ASVTAVVTVYVPCQEKAAAVVDKGDCDMNLIVEKGHVVNMKGNKHWDTAEIECEDGHKLVGNNFI
TCLSSNEWHEIPRCIRKRCETFIAPPNSQMKHKVNSTEVGTRLSISCKEGYAILGIDSVVCQCNETWSE
APKCEPYDCGIPTPPPNGTLVNLNGTKINDTYNVICIEGYSLNGLNGIDIVRCEENGQWSEAPQCMQI
SCGLPDISTNARIVHIDGTSYNASVSFTCADGFYLNGSQSANCDARGTWSHIPKCEKIIDHCELATCYH
GYCQAYKENFTCVCNSGYAGLQCEREIDECNSSPCINGVCVDKIDGYSCTCDLGYEGTVCDIDTDDC
LSGSCVYGKCVDSINSFSCLCYQGFNGSLCETEIDECQSKPCHNGTCVDSTGNYTCVCSPGYTGLNCE
NDIDECISSPCQHGSCIDGIGKYKCLCENGYTSPNCDTDIDECTSGPCKHGSCIDLIGGGYNCSCYSGF
VGNDCDIDINECKSSPCLHGKCIDGIDTYTCDCYNGYNGTNCDNDINECSSNPCRYGTCADLVNRFE
CFCYPGFNGTQCEIDIVECSSSPCLHGKCIDGIDKYTCDCYSGYNGTNCDNDIDECMSNPCTHGTCM
DGVGQYNCTCESGFTNFNCDQDINECSSNPCRNGTCADLVNGFECFCYPGFNGTQCEIDIDECSSS
PCLHGKCIDGIDTYTCNCYNGYNGTNCDNDIDECMSNPCTHGTCIDGVGKYNCTCETGYTDFNCEQ
DINECSSNPCRYGTCADLVNGFECFCYPGFNGTQCEIDIDECSFSPCLHGKCIDGIDTYTCNCYNGYN
GTNCDNDIDECMSNPCTHGTCTDGVGKYNCTCESGYTDFNCEQDINECSSNPCRYGICADLDLSVS
VILVSTAHNARLGSVDQWSRAE
>Mp_P_selectin_01
SAPPLIQNAIYKVNNFGHNAVVGYECENGFGLIGNAAIECLSNSSWTKPTFMCTGCPSAPLISNAILN
VDELIPTEQVLWYKCQHGFTLIGVEHIVCQNHSFWSEPQFVCSLCSAPPNFSNTVFKLNMTANDKFQ
YECTSGLHLIGDNTLQCFPNGTWSARRFTCSECSTPPQLPNTTLQVNDNDTLKYTCNDGLQLIGQDT
LQCQTNGSWIVPSLTCSVCPYPPNFPNAGFQFDMASDDKFLYICNDGLHLIGNNTLQCFPNGTWSK
RQFTCSECSSPPQLTNTTLQLNDNDTLQYTCNNELYLIGENILQCHSNSSWIVPTFRCSVCPFPTKIPD
TDFIFDENSNDRLIYSCNGGLHLIGNKTLHCFPNGTWSNRQFTCSACPVPLNLPNATFKMDLNTSSPF
KYDCKDQLHMIGSGVLECHTNGTWSVPPFLCSGCGPPPSIANASVIIGIGNNQYIPQYVCNTGNILTG
TPSLECQMGGWNIGNFTCS
>Sc_P_selectin_01
MGTSQRVCQSDTSWVPTAPICVQCLTLNNPSGGSVTMATNGHVTTASYSCVSRYSLVGSSTRSCQT
DGSWTGSSPSCVCDDPVIPAYGAVVADGSVARYTCNQGYTLSGDATRSCTTDGTGWSNVDPTCVL
CSNLTIPTGGTFTMATSGTQTYASLTCAQGYSLSGISVISCLSDGSWSQQPTCVLCESLTNPSSGSITYA
SNGSMTTATYSCSSGYYLVGNNQRTCRNDGSWTNTPPTCLCNPPVTPSHGAMVANGTTASYTCD
VGYSLGGQATRTCSTDGTGWDGTQPTCTVCATLASLTGGSITMTTDSLQTTASYTCHVGYTLTGVRS
IICRGDGTWDFSQPVCVSCPSLEAPDSGNVTMTTNNYRTIASFTCVSGYFLSGQLMITCQTDGQWSA
SPPICKCNFPTAPSNGAVSVTNPSSGQVASYSCNHGYTINGISSRQCLTDGSGWDGTDPSCTACASV
SPPALLEFSTSTNGTHTLAQFTCAVNYTLVGVASQTCHHNGSWSHTLPTCAQCMTLINPSGGSLTMI
TNGHVTTASYSCVSGYILVGSGTRSCQTDGRWTGTSPTCVCEDPVSPTHGAVVADGAMAMYTCNQ
GYTLSGAVTRTCYTNGTGWSDADPTCVMCSSLTIPTGGTFTMATTGTQTFASLTCTQGYSISGTATV
NCLSDGSWPQQPICTQCESLTNPSSGSITYASNGSMTTATYSCSSGYYLGWEQSADL*
>Sc_P_selectin_02
MARQQKRTGYEENAKILCPYSFLADYQFTLSSTSTSCDVTSQSSTLTMCADKKVMALTGPSCVNSIFF
TDVNQVWCIDAVTVTSTTYVALYNGLQDNTTVDETDTFLFSCLAVNSAYTAASLVPQYCRENHTSS
QVPADSNQGSTLTLTAISGTLVLLHNVNLTRILAPCPRLVNITEGYVTKVTDGPVTTATYTCNAGFTLT
GDKTRVCQSDGSWSGTGTSCTCPLPTAPVNGSVSVATDGMSLNYTCNSGTVLVGTATRTCQVTLN

TWTDTAPTCETCAAISNLTNGSYVISSTGLTSLVTYSCNSGTTLVGTRSATCKTDTTWSSATPVCVFCS
ELARPWSGDVNVTTDGATSTAVYTCAVGYFLQGSNVSNCGSTGKWDSAAPTCICEPPDVPVNGTLS
VSGDGMTSSYSCLLWFTLSGSAQRTCQSNGTGWSETDPICVQCPGISSPNNGSLDSVTTNGTVTIAT
FSCHVGTTMKGDVTLSCQVSGQWTSTAPVCTTCEHLPLLTSGTVTLVSDNTMTSAQFTCDVGSTLN
GSSTLTCNGNGTWSISGTTPTCVTCPAYNHAGGVTSLSSDGTTSTATLTCGEGFQLHGESTAVCQSD
GTWSVTDAQCVSTSATSSTDTAVVNLVPAVVVLALLAAGFAGAFAVAMRYIFKQRKQKQWHGQW
TNFASRNDIREFPAAGDMVAPSAAPMDHKSHVIVSGHFLPSRAPTAHKTRTSDMPTSRSAKSTSSSLS
FGSPRLEWAKKATVSSIYLGPVEPAPWSKQRPPRPSQPPNGSDLRLDLSKLSTRSENTKSEANVKRVKP
LTLNSVAANEASSRADLGNSGHNMDPSATPIIQNPLHPAKDSPPKGSRIKSRHSTSVNSPRRMTSRRR
KAQKERATTPVKHVLKVEDPDQCKTPRSNIISTVQ*
>Sc_P_selectin_03
MQRDIAKWGITFLVFWMTAVVTTGWNYVDHTTTSMSCSWNNYTLIKKSLTYQEAERFCHLRNATTL
VLKTSSSWTALKAELKDCTMIDPTFDQVPYTIQVTDELPTCFGVYGFISSLTIPYYPCTQRLYTMCSSLP
DCGSPPNVTNGDCRAESTVYLATATYKCHSGYMISGPNSTQCHSNGSWGAAPKCEKIDCGNPPVIS
NAYQNFTSTTYMANVTYTCHSGYRMSRLLDTLQCDSNGSWGVPPTCEKIDCPVVHLANGSMKVEN
EIAHITCDRGYYVNGSTVLACLSNNVWNDTVPTCTPRPSSVICNGKNFTMYYNDNGTFHEARTFCIQ
RHASVFTTTSAVDIKAELQNCNLRPIDEGFIYWATVTGIPRCMGISDLGGQNTTAKPYYCGDKTPILCV
SVPGCSISDPVNGHVTVYNVPQIAAFRCNPGYYVNGSIYLGCLGNNVWNDTISTCAPIECNKPPTVL
NADTNFTATTYLQNATYVCHPGYKISGPTDTLQCHSNSSWGSPPKCEQTVVNCTEPRHISHGSVNV
TGTLNQSIASYECDEGYTLSGDANRTCEANGSWSGQQPSCALVNCTQPSIISNGSVNIYGTLYQDTA
SYECDEGYTLSGDANRTCEANGSWSGQQPNCALVNCTEPSNISNGLVNITGTLYQSIASYYCAEGYT
LSGDANRTCEANGSWSGQLPSCSLVNCTEPMNISHGSVNVTGTLYEAVASYQCEDGYILSGDLNRT
CEANGTWSGQQPSCALVNCTEPSNIFNGLVNITGTVYQNIASYKCDDGYTVSGDANRTCKADGSW
SGQQPSCGRVNCTEPSSISNGSVNVYGTLYQDIASYECDEGYTLSGDANRTCEANGSWSGQQPSCS
RVNCTEPMNISNGSVNVTGTLYEAVATYQCDEGYTLSGDLNRTCEANGTWSGQQPSCALVNCTEP
SNISSGSVNATGTFYQDIASYKCDEGYTLSGFANRTCDATGFWSGRQPSCNRVDCKQPKNISHGSV
NVTRTLYQDTASFWCDEGYKLAGDPVITCQPSGYWSRSDAECNPKHPDIPILASVLNRNSSSNGWLK
ADQREFMLVIENKTFTEALQHCRKLEADLVRFQNPLQYLFLYRILNLTKETFWVGDSLISSTACIVLFED
AADGLPTLAREDCHKPMSSICSRAADIDAILGNPDFVKLIENITTDKKNTGQNRRRLKSAEDHRPSAV
ALGEIGIVVIIIVVSNILLCDLHTFARHFWRRRKSSRGDVQNKQQSKKKCTNKVVPSAQPECPLQATEL
YDVDGQDGCGVG*
>Sc_P_selectin_04
MKVGIAKWGIIFPVFWMTAVCCPVVSLANGAINVENEIAHIRCDRGYYVNGPTKLVCLSNNVWNDN
VPTCTPRPSSVICNGKNFTMYYNDIGTFQEARAFCIQRHASLFTTTSIDDIKAEFQNCNLRPIDEGFVY
WISVTSFRRCIGISDLGGQNTIAKPYHCGDKIPIMCVNVPGCEHLDNPSNGAVHFPSLYIANYTCDLG
YKRNGPEQRRCRPDGTWGLTAPSCVPIKCPAPSPIDNGKVTVIENLYQKVAMYDCNDGYMLAGFGN
ITCGANGSWSRTGPKCNPIRCPYPPSIVNGFVNQTFPWEVNSSINYHCNPDYKLSHPSTTCLKNGSW
SLPIPVCTLNLQTGCTVKSPLHGNSSYLNDSKIVVFKCFPGYRLNGPTHLGCLGNNVWNGTVPTCAPI
PFHYPQSLMNGSSSVRRWSDKI*
>Sc_P_selectin_05
MVPSLQQYSPVYQATTFRDSHLSHVCQMAHGPLNHLTVPVTQCPAFQMVTVIYEGVATVHYMCN
TGFALNGVATRICSTEGLGWDGQEPSCVQCSALPSVSNGSVQMTSNGTTSQITVHCDVGFTVSGHS
QLTCLPTGAWDMSPPVCVLCESLLSPSHGNVTLSSINNIVTIATFSCNLGSTLVGLSTFMCAENGTWN
GTVPQCVSCPTLTSPASGSVAMTTDSITTVAFFICSNGYSLLGFHNLTCMQDGSWDHSAPVCVCEAP

APPTDGSAYSNGTSVIYSCKLGFTLLGDSAGYCKNDSTGWSISVPSCVACEPTVNISGGSQSLQTNGT
TTGALYTCEVGSSLKGSSTVTCNTTGHWVTDPPQCVSCPSLNTVTSGSINITSNGLVTIATFHCSLGYH
VTGADVLTCTTEGSWDAMTSECVCDIPQLPSNGSLTVSGDGQTATFSCEVGFTMQGEATASCSSDG
SGWTVTEPSCVECSSLQAPSGGSVTLVTTGSITSAVFTCEAGSTLELQGASNPTCGSDGSWNVTQISC
VYCPDLNPIDSGAVNVTSNGETSVATYTCDAGYQMVGMSSRACLETGVWQHEVPSCYCIPPAAPIN
GSIAVSADHVTITYMCDLGFTLTGPITRICQSAGQGWTVTDPACTLCEELQMLGNGSTQTSSNGSVTL
ATFICDAGFSLDGTSTIVCLSDGSWSLPVPTCKKCPSLSSPSSGSVTMTTDGSVSMAEYACMPLYHLL
GDGLSYCWNGTWNNTETPVCVCRSPNAPVNGNVTLADDLMTAMFTCHLGYTLDGEPTSTCSSSG
AGWSHSTPSCIVCANLSTPSSGQMNITSDGLITLATFSCNDGYQLEGEATLRCLSDGLWTDQEPICNC
VSPNSPDNGTVIVSDNKMNATYTCDLHTTLIGQSVRTCKQDGSGWSGTDPSCGQCPQLSHPVNGS
SIPSTNGIQSVMTFTCDLGTTLLGDSTLYCQENRTWSSTIPTCVKCIEITSPESGSVTLTTDGIATIATFTC
NASYKMTGYVNSSCMVNGQWSSSVPKCSCEDPTAPAHGNVSNLGSIAVYSCELDYTLAGDVIRTCS
TNGTGWLGNDPTCVQCLPLETRYGATVNMTTNGTTTMVKYSCSIGMTMTGSALLTCLSNGTWNYA
TPTCVTCENLPVPANGNFTLTTDGVTTTAQLMCEAGYQSTGSTQLTCHSNGTWALSQNDLLCGKS
QCHDTTDSRSYLAELIGTSVSLGIVIIVLSVLLATFVYLYFRQKHNAQGYTVELLNRDDAMIQSNAKFHS
MNVEDFSPETDDGSDDFHIPVPSMPLSPAIAPASHQRHVLDNGSIFSTSPAPDNFQHHGISNGHTPR
NFSPMSTTPRSLTSFFNGSSGSNRQFLTPIKNGDIMVKKLNAARGSTSPQQMDLEDTQPSRPTTPMK
HQFKVTDEAKDPRQKSKAISDVSN*
>Sc_P_selectin_06
MVAQRLMVVGIWLLHMFQQGLLSSSPFTCNGVSFIVDLTVKLSYKSSEQFCSNSFSGKLIDPDPRIFEK
CLFKPGDGYFFYVSGKDQSMCKTLAVNGGSFNMITSDCNFEAVPICEVAVTSTSTAVPSVGQISQKSL
SCTSYMLTIGLTGVSHSIAQQFCNTLGATMFTLPSSTSNIMAELQGCNLDSGFDGRQFWIHVLSPSD
NYVLRDVRSDPKVALVPSDALALPMCTIQTGCGPPPEDSHATHSLSAGSSINSVATYTCKPGFKYAGT
KQRQTSMCLTNGQWTKIPQCVKIECEYPPIIQNGACSAVAPFPINKTVFYICEHGYTLSPQSTTCQED
GTWSLPIPACTLNAQPDCGPPPNNPNADVNYASTTYLARATYTCHTGYKLSGSTDSIQCLQTGSWE
PAPQCDKSGCGPLASPMFGNVNIYFVSIAKYTCFEGYKRNGTDQRLCQPDGTWSLEAPTCDIIVCSTP
QNISNGFFDTQSLITQYNVSIEYKCNVGYSLTGNNILTCIDNETWNGPAPSCNPVDCKALPTIPYGTV
NMLGTVYQNVAKYQCDIGNTLIGNGNRTCEADGSWSGQEPICTTVDCKVPQNISNGSVTVTGTLY
QDVARYQCSVGYTLTGDVKRTCDADGSWGGAEPQCALKKCGMPSNISNGFVEVDNQQYPTLVNY
TCNQGYRLTSNKPRVCKDGHWSDSEPNCTQTDCGPPLNIKNGTSTVNGSAIGDVVVYTCNPGSRFS
KGFTLYQKTCQLDATWGETIALCSSTSCAPPSTSANGYLYNITDGPNVFSCKADVIYKGQKSTWCHVS
GPLINPVPPCIPVFCSILHNIPNGTVHVTGTMYHDIARYQCEKGYMLTGNDNRTCRADGSWSGQVP
VCNRVDYIARYQCDEGHTLTGDANRTCGADGSWSGQRPSCSIVGCTILQNISNGSVNVTGTFYQDI
AIYHCAIGFTLTGDANRTCRADGSWSGQVPVCNRVDCKPLQTISNGSVNVTGTLYQDVATYQCDEG
HTLTGDANRTCEADGSWSGQRPDCSIVDCKIPQNVANGSVNVTGTLYQDVAIYHCAIGFTLIGDAN
RTCGADGSWSGQRPSCSIVGCTILQNISNGSVNVTGTFYQDIAIYHCAIGFTLTGDANRTCRADGSW
SGQVPVCNRVDCKPLQTISNGSVNVTGTLYQDVATYQCDEGHTLTADANRTCEADGSWSGQRPD
CSIVDCKIPQNVANGSVNVTGTLYQDVAIYHCAIGFTLIGDANRTCGADGSWSGQRPSCSIVGCTILQ
NISNGSVNVTGTFYQDIAIYHCAIGFTLTGDANRTCRADGSWSGQVPVCNRVDCKPLQTISNGSVN
VTGTLYQDVATYQCDEGHTLTADANRTCEADGSWSGQRPDCSIVDCKIPQNAANGSVNVTGTLYQ
DIAIYHCAIGFTLNGDANRTCRADGSWSGQVPGCSIVDCKIPQSITNGSVNIIGTLYQDVARFQCSEG
YNLTGEANRTCEADGFWSGQDPRCIPVDCGAPQNISKGSIHVTGTVYQGVAAYQCDEGFVLAGDA
QRKCEADGSWSSREPQCNPKHPNIPVLASVLNRNSSLNGWLKADQREFLLVSEKQSYTESLEHCRKL
EADLVRFQNPLQYLFLYKMLNLSKSAFWVGDSLGSLKSAEACIVLYEDEADGLPTLARQGCSEQSSSIC

SRAADIDKILGNPDFVKVIESMTIEKKNTSRSQRQLKSATDHRESAVLLGEVGIAMIVIVVSVIVLFDLQK
LALYCWRRWQSRQGQLISTNKRSSAVASQTLSPADHGTVGRLKCVGLRFREMV*
>Sc_P_selectin_07
MTRDMIKSRTAFLTLWFTAIGTASVVPPGATSFDVSCTGWNYTVYLQNLVQQDAKNFCSQKNATM
FTLKTSKDDVRTEFKNCDIDPKYLGPYYRIDVTDETGCFIVYFGYGAVVRPFQCESGTYFMCVTLPECN
NPPIVPNADTNFTATTYLEKATYVCHPGYRLSGATDTLRCHYNSSWGSPPKCELVDCNKPPTVLNAD
TNFTATTYLQNATYVCHPGYRLSGPTDTLQCHSNSSWGSPPKCEQIDCGNLTNPPHGNVQVFPMNI
ATYSCDIGYKLNGTNKRQCQSNGIWSLSSPFCATVECSSPYNISYGTFTVQNRSYGQRVVYKCNAGY
ELTGDDTSTCLQNGSWSIPAVHCSTVNCSEPTNISHGSVNVTGTLYEGVARYQCEEGYTLSGDANRT
CEANGTWSGQQPSCGLVNCTEPSSISNGSVNVTGTVYQSIASFRCDEGYTLSGDASRSCDANGSWS
GKQPSCSRVNCTEPSSISNGLVNITGTLYQAIATYQCDAGYTLSGDDNRTCKANGSWSGQEPSCSRV
NCTEPSNISSGSVNITGTLYQSIASYYCADGYTLSGDANRTCEANGSWSGQLPSCSRVNCTEPMNIS
HGSVNVTGTLYEAVATYQCEEGYTLSGDLNRTCEANGKWSGQQPSCALVNCTEPSSISNGSVNATG
TLYQDIASYDCDEGYTLFGNANRTCEATGSWSGRQPNCNRVDCKQPKNISHGSVNVTRTLYQDTAS
FGCDEGYKLAGNPDITCQADGYWSRADAECYAKRWLKADQREFMLVIENKTFTEALQHCRKLEADL
VRFQNPLQYLFLYRMLNLSKETFWVGDSSISSRDCIVLFEDEADGLPTLAREDCHKPVSSICSRAADID
VILGNQDFVKLIENITTDKKNTGHNRRLLKSADDHRPSAVALGEVAIAVIVIVVSIILLCDLHTFARYFWR
RRMSNRSDVQNNQQSKKKCTNKVVPSAQPESPLQTTDL*
>Sc_P_selectin_08
MPLISDCNDPPKVLNANWNYTTTTYLGNVTYICHSGYRITGPTDTIQCHSNGSWGFPPKCEQVDCG
SLISPFHGNVQVSPNNIATYSCNTGYKINGTYQRQCQSNGTWSLSAPKCAAIECSSPYNISYGTFTIQ
NWSYGQRVVYKCNAGYKLTGVDTSAHVCKTDHGAYP*
>Sc_P_selectin_09
MEIFHKLWRLTCVVTTMAAVTSVHQMAKVTLPGIDDKSDHVCSNNNTVVNCPENSQIALTSAFHGF
KATGALCTQENGDCLQNDSEEHESLSGKADGSYNCADTYPTSCNASNLQNVSSYCNMSYQCVQN
SDVIQMCDPRTINDREMVYLQSRPYTTSGILPSDTACSCSVETNTFTDINMFIIEMNLNMTSDYCKQN
ITIANRSDDLRSFQCEHLGYNFINKTTIPGTYLNISMINTLNVAGGKFWLGFQASDDNGKISISCPSKN
QTWFLDCQVPDSSMHTQVEISGTSYLSTVTYKCVSGYSPVSGNETRICQKNATWTGDPLQCLYECHE
PPIPDGGNRNFSETVTGAIAYFFCYDNRTLEGDTVRHCVDELGWNGTKSKCCRDCGQPDVPDDGD
VTISDCLFPSRTATYTCRSGYSLIGISQRTCTRNGVWDGATPECRKETFDYKTMLIVVGSLIAVGVVVGV
CYKLWRNSRFGEYILKNWLEYRHKHSHHDSMPPSKPPKQTDNTQKDLKNNDSDEATEIPTNVMLPN
NHSSLESETNTSAIGSDPEQVVHGESVLPKYDSKVTL*
>Sc_P_selectin_10
MDAELLHSIMVNSLLLLYAISAVHGSIIAVSRFSYGFWNRTCNKIFDECCVYKEGDCLQMEQNITDILEE
LSGKKQMTISTRDALAGESCQNQTKETISTYNQLDYFCINETRMLAVSENVSAEGDPGGPIYLHNRDY
PNPVADQDQANYTCSVEATTMTTLTLFGLHVDLKCDQTIDIRDGDSNPMLTLNCASAQSGLEKTFST
TYIRLTFTNDKSQSGGKFWLGFTAAGTGTRLKLRCPAIPKELFTDCGKLEPPINGNVSMSDTTHLSTAN
YSCLQYYNLTSGDATRTCQANGSWSGTEPVCLPAVDCGPLDAPSNGTVNVTGTTYSSVATYACSEY
YTKKSGNSSRTCLANGYWSGEKPVCSLAVECIQNDHCNKAQYECDEGNHKCIYICNNPSIENGNAN
VDIVRPVVSSIVTYTCDEGYSLSGAPTRTCQETTGWSGEQPKCVKKCDHPKAPENGNISLIGSGSTGTV
AIFTCNTGYSIHGPQRLDCREDSTWSDEQPECLLTEKSVQTQSDQTGLAVTIPIILILLAALILFILYHKGL
CNKCKKHEILVEEGRPTDEETKKDIASQRMSNLISKENNQAKDISTASDAVDKKCPIASAEGVDLDHVY
DNPEGETTKKDEKDQANANKESEQESNKKQADNASTYENVSDTKREDADKPGMSPSEGQNKAEVP
VPTENAATKRSSQVIESSGNPDAKGNDVPIESGGPDTKSAAHTVEGVDGSKGDTVQTGSTEKPLIEFD

TDDKKNSKEESTAKEETNAADGVQNTNEGDVKIDKDN*
>Sc_P_selectin_11
MSEILAGFQTKTGTICNIAGPADCLEKMFYDAEFLKAYSGKTDGTAMVLNTLTRSCDTNIFSDVSSYIQ
LNYQCINSTTVRGMCTTVSQTSQPGRVLYLWNEGYPGSGIFKAVTSCACSVTTSSATSDIVMYIVHM
DLYNDASDCYQNLSITNNIGTTLHTFNCETPDFVSITKLTFPYPYIKINLSNMATLTDGRFWIGVQATES
WANIFIVCPASENIDCRVPENGSNANLIYTYTDYGSLANYSCINGYIVDSGSETRRCEQNGTWSGSAL
VCIYECEILENPAHGMVSVKRNVTGVQAVYSCKGNYTLFGSSVRVCADKEGWNGTEPSCEKDCGTP
DSGSYSHVISTLELKQGSVAEYTCFNNYTRLNGNEQRYCIENGMWNGTAINCIYECGAPEPPADGNV
RFDKTVTGEQANYSCADNYTLFGNEARVCADGLGWNGAISTCLKDCGTPDSGSYSHVISTLGLKQGS
VAEYACFNNYTRRSGNKQRYCIENGMWNGTALNCIYECGAPEPPAHGNVRFDKTVTGEQANYSCA
DNYTLFGTEAKLCVDGLGWNGSSPACLKDCGTPDSGSYSNLISTSGLKQGSVAEYTCFNNYTRLSGN
QKRYCIENGMWNGTALSCIYTCASLSLENGNVALVGSGSAGTVAVFTCNEGFSLIGPNLLTCLHGTV
WSDYQPKCKITDTSSTSADITIALSVTIPIILILLTVVIVLILYYRGAFNKGNITTDERGTPCGIGKLNVHLAE
INLSSLNSSRAYQNVEDRNNEATYNARDESCHYYEGLTEECADQPYEELGNEGWKSQAVAAL*
>Sc_P_selectin_12
MESIYVGLILCLFVILEWEKTNGQTEPCQKRADFFPVGRTCRKSCINDDDCVSRKKSCKCDGICGLSCIK
HNQECAPLPSIPNGIAEIRPSNQFGSVATYRCNDGYRLHGNKARVCQGDETWSGSQPYCVISPPEGV
FRKFSHSEEQYCGPAPKVLNAIHDGAPDRKSFNRGAMLLYTCKPGFTLNRDDVVRAWCVGPDVWV
GPNLTCSNAGCSQLSDIENGFVSQSITNGIGSRAMYSCRQGYYLAGRAERVCGIHGEWEGVPPSCEK
VMCFGAPHVDNAYSDGDTQQEHFPSGHQLSFTCEYGYHGDGEGRAMCDADGQWIGLTLTCSAKG
CGHPGEIENGFRTGYHFTYPNKVNYDCNEGYQLIGDTFRKCEASGKWSNSVPSCKPVTCPELLAPVY
GHMIGEGTLYGSVVEFNCSQGYKVVGSRERKCQADRTWSGQPAKCEEIDCGWPKPFYNGYLIGHKT
TVDSIIFFSCKPRSMFDGISFQTQCLQNGSWSHPPPKCWGQCQVPPIPNATLNGKSEMMYVNHND
NITFSCNDGLVPRDRSVIRCNNGTWTSEAECIPAPCAKPPPHVHNGLRIFIGQHHGNQAKYKCFAGY
RLTGLASPYLTCTFGEWKGGQPHCEEYYCPSPDSPAHGTVYKKTSDGNYLFQNYIRTIKHGDRLVFGC
DNGYQLDGPSGATCVDGKWQPPLTSVCKPAVHPPFLKLWTPIPNMFK*
>Sc_P_selectin_13
MQALLWIKDCGTPVIPNGNVVQGTIQGTLYGDTVDYTCNDGYNSSNSQIVCQADGEWSSSTCTKK
DCGKYPMIPHATINQTSLSGNMYNDSFTIICDNGYLAETTKIYCQSDGTWSMTDCVFQGSCPHVYVP
YGNVTIVPWSNSSDSSEETFREETFQTDEDSSVDVSNENDTSSSVVSILPGYTLTIKCNKGYTLVGTNIR
CNESFQWDGQVPKCVPGLGYECDGICYGNNTVCTASGVCGCDVGLFNVEGKCASGCPALDKLDH
GSMSPKQSTYQVGQTVDFSCDKFYTLDGTSNTTCLENGEWSDKTPVCKVGPVPEKLKGASKITFTVK
GDLVPDTSAELQKLKDQLTGNDGYLSSVKVDDIRFTASDNMTLVEVLLEDTNLDGSIITDAGVEFLQH
LRKKDHTVDMHGEQMLVVGTPQYSFGYPQTASIDSICFWHKFVDTCIQLGKCDMIGGKQMCKCDE
TICHFNGLLILPYVLPVVALVAIFLFIQYGIIYQCSPKPPVQSMKLTKEIPDDLL*
>Sc_P_selectin_14
MIHGTTAGSTTVGGSVSIQCEAGYHLIGPITAQCLSTGQWTQLPVCLIDCGRPPFSRGQLSGWTYEGS
VIQFTCNPGFNLSGPTTAICQPDGNWTDLPTCLYECGQPPLTVNGNLSYTCTLENCLATITCSPGYKL
ATRNVLQCTLFFGWEPNTLLCERVDCGLPVISNSAVVGTTTYLGRVDVTCDVGYVPLPGLPTSAVCQ
AYGTWTVLPYCAFDCGNLENPLNGVVELECTFENCSAVYMCFYGNVSIPTGGSNVEWNLMCSSGA
KGWLSVLPICSPPPDIVYQQINISLALPFKFQLCSDDFDSIVLISTLSGQLSAVYTDVLGDDFFGLLIYNIT
TNCTISYTVLMADTTPTLSAHAASIAAGLPVYIRGQTYTLTLLTINGQPAAVNGSLSDLACDVFQQTN
GPCFLGTECDVDTTATPYCRSSASGFFPAVIPVMYNPVPYSFPSMYGPTRWLSEDIMLETISLASETSA
VPRVGVGFAPHEETPLPGSTLDETSLAGHPAEESEIHRPWIDQTNLNMSFGIARPLVDPSPNPLMLNR

GNADVDV*
>Sc_P_selectin_15
MPPIIHRKYRYLVYSDRKMKTYENIQNWLCRPNGKPAYDCGHPQNISNGTIVNMTGTTFTSLVLYKC
NVGYKMNGSAHIKCTANGSWEQSPMCDIYDCKTPPEVINSTKEYNRTTYLSVANYSCDIGFDLLGNN
IVSCSANGSWERGPYCEIKDCSSPTTVRNSTLVFNTTTYLSVANYSCHSGFDLRGSNSVTCSANGSWT
QAPGCEIKDCGPVHSPTYGSVRGSNTTSYTSVKTFSCNTGYNLIGSDTIICNASGQWNVSEPSCQIKD
CKTPPAVNNSTKEYNGTTYLSVANYSCDTGFDLQGNHTVSCSANGSWEQTPYCKIKDCSSPTTVRN
STLVFNTTTYLSVANYSCHSGFDLRGSNSVTCSANGSWTQAPGCEIKDCGPVRAPTYGSVRGSNTTS
YTSVKRFSCNTGYDLIGSDTIICNASGQWNVSEPSCQVKDCKTPPAVNNSTKEYNATTYLSVANYSC
NTGFDLQGNNTVLCSANGSWEQTPYCKIKDCSSPTYVRNSTLVFNTTTYLSVANYSCHSGFDLRGTN
SVKCSENGSWTQEPACKIKDCGQISAIKNGTTLGANVTTFTALVQYICNQGFKREGAHVTYCNESGQ
WNATAPNCTIHDCGPPPSTGYGYYKLGPLSLTVFNHSAHYTCQSGFETTNPTTIVCNASGVWDKPAP
ACTDVNECSDSNKNQCHIKANCTNTVGSYSCKCVDGFQDMSQGQGHTCEDEDECAGHNLCDRL
HTAICLNNYGGYICICSQGWEGKYCHQDINECLPSNEKKACAELAMCHNTQGSYTCTCPPKYPQGD
PRIKCFEKVVLDFQTHITPKADDNGVENLYSFPYRFPYFGKEYVEMRVSMNGFFTLDYDRIYRQDGPQ
TIEDWFKYANKVPLVAPLWTNINAAGLTNGGLYIKIFSNNSTQKTEKDDFQHITSVFHNYTDFSSFDPT
FAITVTWRNVSIYSHLSAPEMLAYQNATMQAIMVTDGIFSYL
>Sc_P_selectin_16
MNGSSHIMCTENGTWEQEPSCSVYDCGTVTAPTNGSMAGNITTTYGSMKRFECDTGFYLFGSDTVV
CNASGQWNVSAPTCHIKNCSTPPFVMNSTTKYNETTYLSVSTYSCDSGFDIQGHNTVTCTANGSWT
HGPWCKIKDCGEPKNISDGGTFNSTGTTFTSIVFYKCSVGYKMNGIANIKCIENGTWEQEPSCDIYDC
GTVIPPTNGSMNGHNTTTYGSMKTFNCDTGYDLIGSHTIVCNASGQWNVSTPTCQIKNCGPVPPIE
NGTTLGANVTTFTAIIKYKCNDGFHQIGELVAYCNQSGMWNATAPYCKIKDCKRPPNIQHGDYKLGP
SGLTVFNHSATYTCQSGFETTNATVILCNASGVWDKPSPICTDVNECSDASKNQCHIKANCSNNVG
SYSCKCVDGFQDTSHGQGHKCEDEDECAGHNLCDRQHTAICLNNYGSYICICSQGWEGKYCHQDT
NECIPNHGKKACAELAVCHNTIGSYTCTCPDDSPQGDPKIKCFEKVVLDFKTNITAKADDNGVENLYS
FPYRFPYFGKEYVEMRVSMNGFFSLDYDRIYRQDGPQTTDDWFKYANHVPLVAPLWTNINAAGLTN
GGLYIKIFSNNSTQKTEKDDFQHIIDVFHGYPGFSSFHPTFAITVTWRNVSIYSHLSAPEMLAYQNATM
QAIMVTDGIFSYLMLNYDQEQWTLQPTPYRDMVAGYVGIDGNPVFLANSSNFTNLNNDSNVDPHL
NGRYIFNLTKVTDVLINQSLCLKFHNNKPAMNWIKVEAAKSYACPCSEVIMQYDYTFHVVPNLDKGI
DPKFPVTCYEEWFYNSGTRQRCCYYFGALKTGSPSGGFVQFEDDTYNVMDSYYQCCGGDYHMCPL
FYEMNPSDDCSRWTPDDEYAPFRRKRSEFKPYFVDDMPSSYDIAFAAQTELSLCFLPYLVTYADEPLK
VSIYTDIDDVDYDWSEATSTFTWNIDRKYVTRNVTLTFVFGDSMSYMTMTYEPKIRVCWCQKVNQC
DFGQRGPFQGAGIILASAVPCRCGPEERGRYCEIQQQTDCKPPCFPGVPCIIEANRTRCGPCPFDFQG
NGVFCSKRIVLKTDQVTEKIIEILGTTMCVVWPTALGTTDVAKGSPN*
>Sc_P_selectin_17
MNYRPDLSIRYELSPRYVCQVRTIIPICLSGTNYHPDLSVRYELSSRSVCQVRIITPICLSGTNYHPDLSVR
YELSSRSVSQVRTITPICLPGTNYHPDLSVRYELSPRSVCQVVYAPFDTSTRSFTSCLIVNDDLISRVYVSF
DLFAMLVKSGVGEPCSSDDACFDPNSECSATSSLCTCKNGYGVGLSTSETRECEKDCGTPPSVEFSTN
QYSGTTVGFSVNYQCASGYSISGVTQLTCQENGSWEEPAPSCVPDIGTMCSAVTDCRDPHAQCTG
GTCGCREGYTEREGVCRLECSQVSLEHGTVGVTGNGVGDTATFSCNSGYTLIGPEVLTCTDSMVWD
NELPECKKATVLDCPSVKAPINGETEIQLLRVRKPECGVGMCPNVFQAVPPQQPHKMVPLVVTGLVA
TFQCNPGYRMTGVREITCTEGRWSGAFPECTKECVALSTPAGGNVVIDNDEANFSCTTGHELVGPTQ
LRCQNGQWNGTSPQCRIYCPPLVNPANGAVVTPNGIFVGQTATYTCNTGYGLVGSSVVTCLSNGR

WSQIPPVCQLMQETFPDKFTADTQIVFHVTPGGPQLTNELLKSHIKDELLRGRDSVGFVPLKEIICEETP
CDMEAGLRFVGILTIVILAADALYLLMLISCYYQCGHGHQKAIRPPPPRKYSMETEVVFPTVMPRYANII
VEPVFMEPIVEELVEIEEVVEEPEYIEQSNYTMDIIQDRGNQYI*
>Sc_P_selectin_18
MCTIVDCTSSLPTFSNGNVVLDTTSTTYGSTATVTCDTGYTASTASISCPANGAWTTATCTIVDCTSTL
PSFSKGTAALDTETSTYGSTATVTCDTGYEATVSSISCPASGIWPTPSCVVVASIDCSSTLPIFSNGVAVL
DTGTSTYGSTATVTCDTGYNTATSTISCPASSVWPTPTCTIVDCTSALPSFSNGIAAMDSKTSTYGSTA
TVSCNTGYTTTTSSISCQANGIWPTPTCTLVDCTSTLPTFSNGNAVLDTETSTYGSTATVSCITGYTTTS
TSISCTASGVWPTPSCTIVDCTSTLPTIANGNAALDSGTSTFGSTATVTCATGYTTTTLTISCPPSGNWP
TPSCTLLDCPTLSVPSNGAVSINGFVATYACDSGYTRVGTETQTCSAGSWSGVAPSCFKNCPAVSSP
NNGNIVSYSDFYIGQSVVFECDPGYLQVGSDVITCLAGGIWDSVPPVCVGDFIPSQFTSASSIVFNLM
EPIPRRPSPFSSAQISQAVINNLMAGSTIARVPYTGIDVTYDETTFEYMVTIQFNKTTLSEDFITVLGTNFL
QMLKDNNNAVAMSQDVALTVTGSPTFTYGVTRKAPITSSCFWYQERGTCVDKGLCELKDGQQLCK
CEETTCGDNNLAIILGVCLPVIFLLMVALLLVAYIFRRSRRAGKNASGTDETIFTSSDNQTMPIEPSSLFA
ENVPTFVHILRSPSIDMLHGESRSSLPLDMAFSEEFEDIEVPFQNQHMYRRSMH*
>Sc_P_selectin_19
MTTYLSEVKYMCDTGFNLTTASDTRICQINGSWSDPVPACEIVDCGDPEFISNGTLVFNYTTYQSEVQ
FVCDIGFNITTPSDTIRCQANGSWSEPTPACELVDCYDPGQPANGMMNFNSTTYQSWAQFVCDTG
FNLTTPSDTIHCQANGSWSEPTPVCEPIDCYDPSQTVNGTVNFNLTTYQSEAQFVCDTGFNLTTPSD
TIYCQANGSWSGPIPSCEIVDCHDPSQTANGTVNFTLTTYQSEAQLVCDTGFQPHNSIRYHTLSG*
>Bp_P_selectin_01
YIGCVDKITIDANITGTVTTNSGMTSTFCIQLCRGQDASFAGIHSSTTCQSMYADDVFGSCDELLSYGI
TEPGWYYIKPPNATQSNKVKCFQPESSLTSLELNPTITATSSHTDYTPSKSRIRWPFVVDDVWKPAVN
YTNQLLTFTFSADILLTGIVTQGNPLDNNSEWTTSYSLIYYDVLLGNDQDYSRNGSHVSVYKTCPEPTV
IPYSNQTYDTSVQGSFVYRSSVTYTCDVGYIFNDRTRSKSLVCGQDNQWTGDIDICEAVICDDLALPS
NSTANTNSTYYGIVVNVTCDIGFNIEANVKSQYLVCTEHNTWNSTIEDCKRINCGEPPTIALADRVGH
LYNFNNTVVYMCPDHMWFGTSQFNISIARCTEEATWENIPSQCTYYECPEPTSILHASYVRTGADTA
VYTCDSFYRFSNNGNSMTLTCNQNFTWEQIKDVCRRQDDQVCFRSTTSTIMSNIVRHSYTTDDILSE
FVPRSRSSCAGACLSDWQCQGYNYKTDLCRLFNKKPTTAELKNDPEWQYFEKNNEELQSC
>Bp_P_selectin_02
SICANLIDAQYAVDATKPSGMIVSTGNTFYSNKKTCSWSIVTNIGSTIRVKVSWTGGMAGEIVLGELTG
NVSPLPTFYSSDNVFQMRLLTDSTIIKSGFSISWTTDVPEVFNDLQMLPATDTFKDLKSPLYDTEYLPHE
KVFEWVITASSDDVVTLQFENIDIDDKGKLYLWDGDDVTLTPTLTLNTVPTQDIFISRSKSVRLMLMSG
SDSFTFSKGLYIKYWQDCPQLTVSSQTQVMYTEERVFGSITTLTCDNGYSFVQEEFNQQSSVPMECRY
GGDWDKPRVPNCMINYCPVPPSVANGYIIQSSGVKYGGNVTYKCNDGFSILGTEHMNCKSDGTWE
VNPICRADGCSAVGVLENGAHSVLEGNGVDFASVVQYECDPDCPQLTVSSQTQVTYTEERVFGSITT
LTCDDGYSFVQEEFNQQSSVPMECRYGGDWDKPRVPNCMINYCPIPPSVANGYIIQSSGVKYGGNV
TYKCNDGFTMLGTEHMNCKSDGTWEVDPTCRADGCSADGDLVHGDHSILEGNGEDFASVVQYKC
DPGYETYGNITKYCKTGGMWSHTPPTCESKYCPCILLLQDMWNMVSYTSNL
>Bp_P_selectin_03
MKDKISDLNIFVGCGEFPVPVGAVDMTVFRGLYSTINLTCTDDYITTGSLDVKCENDTWDIGNFTCW
WKDCGEPSALPGGQRSFSGTEVGFVTTHSLELINSQYVVQYQCDSGLRLIGNDLPDCQPDTNWTKP
TLLCTVCQDPQSLEHATYKVVNTNVLRYECNSSLGLVGNATIECQPDANWTYPRFICTVCQEPPRLPL
TSIQVESFQSSSIVQYHCIGSLNLIGDGVIYCWKNASWSTPAFICSELSMSNTAFSFSKACPYPPQVLN

ATFRLEQNNSHPFQYECDDGLNMI
>Pf_P_selectin_01
MRCVNLGRRIPNGRVNIVPFNKFGAVAKYTCNDGYELKGLAIRVCQGNEEWSGQEPRCVLTTRVPD
KTECGRPPDVANADFYQNRGNSTYPLGTMLEYTCRDGFQKGKNDADKAWCVGMGTWVGPRMKC
YMTCTSPPVITHANHDGPKQPRYSAGFQLTYNCDMGYIRESDPRIMCGDDGEWTRLELRCLPVNCG
FPGELDNGWRSGHIFHYPEGIQYHCHEGYELRGSPNRTCQPNAEWSGLPPTCHAVDCPILQAPANG
TMIGVGNAFGTVIQFKCKGNFHLSGSEERRCQANRTWSGTAATCSAPCRDIPPPINNGLRIYTGRQN
GRKAKYICLNGYRLAGMDVDNATLICQQGTWIGGAPRCEQVYCPNPGEIEHGKIYKLENDNSRFEFK
TYIHEIEHGQRLGFECNIGYTLRGVSGATCIDGTWNPPLQSHCEEAHHLPFPPWWHPISNVNYHVK
>Pf_P_selectin_02
MASASVVKTTDSDVAYLDIADWPETLRDCDGFSCRDRERCTVDKSGRTMCIPSECPDVTRPDYGTILE
YGRSVGDIVTYECDVGYERDIYAPSVCQENGQWSYLPRCQQTVCPPVPDISQARANTTDRVSGTVV
SYICNSGYIGKGPDITRCDNSGQWTDTDFECFYDCGTPDVITGASVVNNYTHATYTCTSGFMSVIGQ
SDDTVVCSESIRGWTQSNFLCVADCGAPTSISNTDNFYNETHTIYYCAQGYVSLSGPPRETIECSEKDR
AWTSASISCVDDPCQNGYNLIYEVDETGSVLTGSFADLMDKVMSGLTLRIMISESQSDKHHIIEVSSVT
TSTNDIIASSIHNDERLLSSTTSNLVVNRLYYTVSSSGLITESRWMIGMQTMLSQTSYQSHTSWFADM
DSFQSIYTYDPVTAIASGSITDLVEEASKGSEIQVMIDNGVLHNPGSIEYTTMQITIQNVWYLGNLISLSP
TSRLHWSLNLLDPTDGSISEITWYTESNDNHFSDRYAFQDWIYNDCWDLKYENDASGNSVTGNLYE
LIDLIKSGHRVRVDTEGRLEEALSIRVDNGVVWVQTLDSAVQDMNANFNANNPSRVFRSISTSGVIFA
SHCGNGECIAQTVVKKNMKWYVDKAVWENMLVHSQQNQAGGFTPQSAMQNLLNKVKTGRKVR
LHINDYSDLDIFVNADHVTIDGGTSTTDASSVNHLYVTPGVSIPMSPDWQFLYVTTASYNAVIVRMN
YFFHQQTFEDEREVETTWFVN
>Cv_P_selectin_01
MGNCIFWIAVSLIFALSINNINQVCATRFCDTTWISKTHPNMIVTPSVSKLSYGEEVFFSCKPGYLLHGP
NRSECMSNGRIPVNTMTFYCEDLGIHCEHRQLDQPHLTVSPIKDIYRYNEEVSLRCDEGYILRVPSSAR
CTEDFVFDIPSTSVCEAEVRCHKSDSKINVLHLVVTPSKDEYRYNETVSLECEEGYTLQGPSTATCQQEF
NILSPPSCIATYCDASWIYQQSHDIVYSSNSDPSKLKVGESINFSCRRGHRLRGARSVKCLPSGELEGD
GLLSYCEQIRCSRNTELGDIISTPDKNFYDFNETIHFQCAGDKTVYGPTSARCSEQGWEYEGSSMPVC
ANRYCDVTWLHRQGMYLTPNVDGKAPVGTRLQANCLQNKRLRCGKSAVCMPNGRLNGIEHMCF
CEGE
>Cv_P_selectin_02
MGPACQYVNLALNSLNSDYVFDGNMSTDRPISAATVRLNGHFVIEAIRIHTRIYYKRPSSTVIIKDNKTS
VLFDGRFPSTMPTIYLKRPVLTNQLNINFSSQINIKEIEVFGGKNVALWKPTYQTSTYNQNGINMTSDK
AVDGYAEGNLSANSCTHTSGGEAPRWTVNLQKAYSIIAIKIKNRDTNRNRINGFLLYGTMDNGTYILIY
NDVNPAQERSEIFLDSSFMPDVPISVVSIIGNTNGEKILTLCEVFIYACIPKADGSDKCDSFCPKNCMSG
SVCSKQLLGCPVCENGWTHSYCDKKITCQRPKVYDTHLSVNPWTDSYSYNEVITFSCSKGYQLKGAT
STTCGNNGYLQGRLPNCTAITCQKPRLAGTHLSVNPMKNSYAYMEVITYSCYEGYQLSGSTSETCGN
NGHFQGRLPNCTAITCQKPRLSDTHLSVTPLKNSYAYKEVITFSCSEGYQLIGSSSKPCRNDRDFQRGL
PNCTVITCQRPELSRELHIASNPFKQTFYYNESVTFYCSVGFSLQGPSVKYCRHKGEFQNDLPSCSNVT
CQRPSLSNALQLSGTDPLQQTFSYNESVKFYCSDGFHLQGPLIKYCRQSGDFKYNIPICTAKSSQTSFT
VGFVSGGVAGVIAITSAAVVFIILRRRVRRKKENGKKIDEGRSQESDNKDHKYDGIDDQPSEMEGNYT
GISESDDDKNHQYMELNAV
>Cv_P_selectin_03
MSRRSGDMGRSIIVCFLVIRLCSSVTCNRPAIPYAAIIQPNQPTYSYNAAVTFSCSPGFKLIGESEKRCQ

QTGQFGNLPSCTVVTCNRPAMPYGTIIQPYQPTYSYNAAVTFSCSPGFKLVGEAVRRCQQNGQFGN
LPSCTGVTCNRPAMPYAAIIQPNQPTYSYNAAVTFSCSPGFKLVGEAVRRCQQNGQFGNLPSCTVVT
CSRPKIPNAIIHPDKLTYEYNETVRFSCPLGFSGAFVKSCQQDGHFEGDLPSCTGVTCNRPAIPYAAIIQ
PNQPTYRYNSAVTFSCSPGFKLVGEAVRRCQQNGQLGNLPSFVSCNKPTIPYAAIIQPNQQTYRYNS
AVTFSCSPGFRLVGEAVRRCQQNGQFGNLPFCTVVTCNRPAMPYGTIIQPNQPTYSYNAAVTFSCSP
GFKLVGEAVRRCQQNGQFGNLPSCTVVTCSRPKIPNAIIHPDKRTYEYNETVRFSCPLGFRLSGAFVKS
CQLDGHFEGDLPSCTVITCQRPQLSRELHIASNPFKQTFYYNESVKFQCSAGFSLQGPSVKYCRNNG
DFQKDLPTCSNVTCQRPSLSNALQLSGTDPLQQTFSYNESVLFYCSDGFHLQGPSIKYCRQSGDFKY
NIPICTAKSSEASFTVGFVSGGLVGVIAITSSAVVFIILRRRVRRKKENDNKIDEGRSQESDDKDHQYDGI
DDQPNEMEGHYTGISHSDDDKTYQYMELNAV
>Cv_P_selectin_04
MDNLGICRPVQVMISVSCNKPTIPYGTLIQPNQHTYRYNEAVTFSCFPGFRLVGEPLRRCQQSGHFM
NLPSCTVVTCSRPEIPNGFIQPDKRTYEYNDTVRFSCSLGFGLIGESVQSCQLDGHFERDLPSCTVATC
SRPKIPNAMIHPDKQTYEYNETVRFSCRLGFRLIGTFVKSCQQDGHFEGDLPSCTVITCQRPQLSRELHI
ASNPFKQTFYYNESVKFQCSAGFSLQGPSVKYCRNNGDFQKDLPTCSNVTCQRPSLSNALQLSGTD
PLQQTFSYNESVLFYCSDGFHLQGPLIKYCRQSGDFKYNIPICTAKSSEASFTVGFVSGGLVGVIAITSSA
VVFIILRRRVRRKKENENKIDEGRSQESDDKDHQYDGIDDQPNEMEGHYTGISHSDDDKTHQYMELN
AV
>Cv_P_selectin_05
MYRYNEVVNFICSDGFTLTGLSQKRCQQTGDFNRNVPNCTEITCTRPLVSNQTRLEPEQKIYRYNESV
TFSCIAGFSLTGQNVQHCKQNGDFKRNVPNCTAIQCLPPTVLQPLHITTASNKTLYNYGDFIEFTCPL
GYQALKSSKKYCKNNADFQLGLPICKVITCQRPQLSRELHIASNPFKQTFYYNESVKFQCSAGFSLQGP
SVKYCRNNGDFQKDLPTCSNVTCQRPSLSNALQLFGTDPLQQTFSYNESVLFYCSGGFHLQGPSIKY
CRQSGDFKYNIPICTAKSSEASFTVGFVSGGLVGVIAITSSAVVFIILRRRVRRKKENENKIDEGRSQESD
DKDHQYDGIDDQPNEMEGHYTGISHSDDDKTHQYMELNAV
>Cv_P_selectin_06
MKQDSRYLSFLVLSFSLAQTIQGLSCPEREGTPGRECQKSCHPEHSPCKSDRKTCECDGRCGYSCISQ
NHQCEKLEWTIPNGRVDINPQNMFGGIATYNCDWGYEIQGQKDRVCQGDGLWSGEAPKCVLSRG
NDKEGCGPPPVIDFATHNEQELDPGVRYPSGTMLQYSCIEGYMKGADNVERAWCIVGGMWVGPKI
VCRAAGCPKPPDILNGGIIIPASTGVSSVVQYYCNPGFQLTGRANRTCQSDSTWEGDEPRCEKGGSE
ACGPPPHVERAFHDSEGIVRFPSGSQVQYRCQEGFMLTERHANTRALCIYQRWEGLRMSCKPVECG
FPNTVENGGITGIDFTYPHTVHFVCNQGYRLVGNDSTTCQASGEWSAESPTCVAIDCPTLRSPVGGRI
RGSGNAFGTVLRFECDNGLQLEGSIERRCLENGEWSGNDVRCTAEIDCGRPKNFWNGYVLGEETTLG
STLFFSCIVRTTFDGPYFETKCLSTGRWSNPPPTCWGQCSIPNIENAEINVYPVGTWINHGKTFEYSCK
NGLVPIRSRIVTCFNGTFNPSPPKCVPAPCPSPPPHVDNGIRLFIGQEHGRRARYICNNGFRLSGMHQ
SSPYLVCDKGRWMGGNPVCQEFFCPNPGTITNGKIFKKEPNNIRFNFPQYITTIKHGQRLEFQCDPGY
EISGASGATCINGQWVPPLGDAGSHCKPQQYLPIPRLWTPRPNRNFYEE
>Cv_P_selectin_07
MKQDSRYLSFLVLSFSLAQTIQGLSCPEREGTPGRECQKSCHPEHSPCKSDRKTCECDGRCGYSCISQ
NHQCEKLEWTIPNGRVDINPQNMFGGIATYNCDWGYEIQGQKDRVCQGDGLWSGEAPKCVLSRG
NDKEGCGPPPVIDFATHNEQELDPGVRYPSGTMLQYSCIEGYMKGADNVERAWCIVGGMWVGPKI
VCRAAGCPKPPDILNGGIIIPASTGVSSVVQYYCNPGFQLTGRANRTCQSDSTWEGDEPRCEKGGSE
ACGPPPHVERAFHDSEGIVRFPSGSQVQYRCQEGFMLTERHANTRALCIYQRWEGLRMSCKPVECG
FPNTVENGGITGIDFTYPHTVHFVCNQGYRLVGNDSTTCQASGEWSAESPTCVAIDCPTLRSPVGGRI

RGSGNAFGTVLRFECDNGLQLEGSIERRCLENGEWSGNDVRCTEIDCGRPKNFWNGYVLGEETTLGS
TLFFSCIVRTTFDGPYFETKCLSTGRWSNPPPTCWGQCSIPNIENAEINVYPVGTWINHGKTFEYSCKN
GLVPIRSRIVTCFNGTFNPSPPKCVPAPCPSPPPHVDNGIRLFIGQEHGRRARYICNNGFRLSGMHQSS
PYLVCDKGRWMGGNPVCQEFFCPNPGTITNGKIFKKEPNNIRFNFPQYITTIKHGQRLEFQCDPGYEI
SGASGATCINGQWVPPLGDAGSHCKPQQYLPIPRLWTPRPNRNFYEE
>Cv_P_selectin_08
MKQDSRYLSFLVLSFSLAQTIQGLSCPEREGTPGRECQKSCHPEHSPCKSDRKTCECDGRCGYSCISQ
NHQCEKLEWTIPNGRVDINPQNMFGGIATYNCDWGYEIQGQKDRVCQGDGLWSGEAPKCVLSRG
NDKEGCGPPPVIDFATHNEQELDPGVRYPSGTMLQYSCIEGYMKGADNVERAWCIVGGMWVGPKI
VCRAAGCPKPPDILNGGIIIPASTGVSSVVQYYCNPGFQLTGRANRTCQSDSTWEGDEPRCEKGGSE
ACGPPPHVERAFHDSEGIVRFPSGSQVQYRCQEGFMLTERHANTRALCIYQRWEGLRMSCKPVECG
FPNTVENGGITGIDFTYPHTVHFVCNQGYRLVGNDSTTCQASGEWSAESPTCVEIDCGRPKNFWNG
YVLGEETTLGSTLFFSCIVRTTFDGPYFETKCLSTGRWSNPPPTCWGQCSIPNIENAEINVYPVGTWINH
GKTFEYSCKNGLVPIRSRIVTCFNGTFNPSPPKCVPAPCPSPPPHVDNGIRLFIGQEHGRRARYICNNG
FRLSGMHQSSPYLVCDKGRWMGGNPVCQEFFCPNPGTITNGKIFKKEPNNIRFNFPQYITTIKHGQRL
EFQCDPGYEISGASGATCINGQWVPPLGDAGSHCKPQQYLPIPRLWTPRPNRNFYEE
>Cv_P_selectin_09
MEKVGLMLTSILSIFLLAMTTNGLSPPDFLSSWTPISAYTNPEVDITHGLGEMPMKVDVQVRVIHSVH
GELIFTATGSGQTSDDPTKPFGGVIYLYNSNSVKIMAPGPHPSCSPTCPGGVAYFGGAGFTGPWSSSE
VITNGEVRVRAWKTCSFIKPTSISGAYWMSNSRTNNFHKIPIGSSPDLVLVKVFFDDGWIMDAQGTVS
LGDGASVGGVQYVFDVQNVYIRTPSNGKMFNGINWGTTGDMEHTNGTVRVYTWMFSELDYHPGL
LMSVYTGSTNPKQETMTYDPSNETLIMMVQAKSQYGLTFPLSGTAMVNGDGMTDHYGSLFFAYDN
NHAIYFWKPTSSHGCDIMIGGKWSDNILDGNTECSASPDIYVRYMKAVIDELPCGEKGCSGTPAGPFC
DCSGTGFEGQYCSSAVACPLLVFGVDVVMANFENREYTYMESVAFTCMDGYEYSSGSDNRSCQAD
ATWSGGPFICTPVPCPDAELPTLGFINGTYNKWFGENVTYVCNPGHDILSGDSVRTCQANQTWSGS
PLECALSCGDPNPPPENAHQTNFGNTYGVVATFACDLGHEYSAGDTAMLCQDNGSWNGTALKCS
KVPCGQPNDGANADTEVMGTVYGSRVYYHCHPGYHYYYPPYAKYYMTFYRTCQHTGVWQSPINC
TKKICGYPGSPFNAALDAANSNVGNYEYMSIQRFNCHQGFEVITGSLQIECTQYGNWSNSAPVCNIR
KCTKFGDVANGTVHYTGLEYMNYAVTVCDTGYAVASGSAMRLCNESGYWTGSAPVCQRIRVIYFVD
PIDFNISLPSIFDLEELTNEIKINPKNTSSYQRSLKSQHDPRTSSIAIGVSGLLIMLGICGYICSLDLISHIRVN
GKVKITQGLQKQDPNIVAKNGSSDKYAKKGGRKNYPESVDSVDHIYA
>Cv_P_selectin_10
MMLGLLLCLFHGIIAHYLGYAAGMTPYEPTFISNWITAKAQDDNKVEFFVPFPSDLHRTEPPVKVIVEV
KVTDSGDSYIFNAFGSAPRDDDVNEKYGGVIYFYNATGVLILLPEKYENDPDNIKTSNGKAVYLGTSDF
WYQPTSHVGDTIKEEYVDAQVRVKMWLPEHMPPDYDSGFIHKLKSKDDSNYAAGMSNNRTPLQTY
LEVPISISSLPAMVVVQAKPVTPFSDFPGLVSEGNGMPACMGCGRQAGGLAFAYNQTHVRLWVKKD
YPIISASDGWGDRHHLDPNRLVSSEVTVRVLAWSFDNAPASCRWMEKSSFTITELTIDAPRMLFLQPR
NIDASLVVVSISPLVGINSDYVFYGAGSVVTSGDYQTDEKYSGVVFGYNEHGVFVWRAKPSTHSKAFL
ITNPWGDGQQSQRTNDVRIDIQVFGLMARGACNATELALINARVSVPAVEPCQKENITCTFGYELKN
DTYSNVFCHLNAVWAARVPQCTQIFCPDEPDPSNGTVIHQNTTVNGEISYSCHPGYNVSAGNLNRK
CLSNGTWSGDPPICSEILCPQIIHLENGEIVQFADGLSSFNSVNATVSFSCLPGFAISGDVTLTCLITGSW
SAAPPVCTEILCPNLTDPSNGVVERMNNKVNGTVTYACNTNYYVTSGNLRRVCSINGHWSGDPPTC
SEILCPQIPSLDNGIVTILPNPTALVHVVNSTASFSCLPGFALSADVILTCLITGNWSAAPPVCSKIILCPN
VTDPSNGTVAQINNEVNGTLLYTCNSYYHVTSGDLQRVCLNNGSWSGDPPVCSELLCPPIEDLENG

VVKLFSESNSVNVTASFSCLPGFELSVNVTIICLISGNWSAAPPICSQILCPNVTDPLNGVVVHVNNNV
NGTLTYTCNKNYHVTSGNVSRVCSINGSWSGDPPVCSEIACPLLTIPANADVSFSPDVSSTNSGGISIG
TSASFTCKPGYRLNVSVSVIYCDKDGSWTGTPPGCKEILCPDPKIPPNSTLTSVNKSVNGTAQFVCNK
GYSHTNGSLQQTCLEPGVWNGTATVCTASGSCLCPCQYVQVPKYTDVNDEKLTKEIKEMQKELQVL
KNKTSAALRKKISIKDFRPSTNVSGAVWMVLLLFLVAAIVVPDAINAFQNIVHYFHSRKHTCNKRLKN
W
>Cg_P_selectin_01
MKPDCRIFTFLILSFTLFQDTQGNDCPERVPNPGRECQKACHPDDNPCKSGRKTCECDGLCGYSCIS
QNHRCENLARTIPNGRVNIKPQNMFGGIATYECDWGYEIRGQKDRVCRGDEKWEGEAPSCVLSKGS
GQPILSKEGCNSPPVIEYAIHNGRELGEGERYPSGTMLEYSCIEGFMRGSDYVERAWCIADGEWVGPK
MVCQAAGCPKPPEPLNGGVIIPASTGVNSVVRYYCNPGFELIGRSERICESDSTWTGNEPRCEKGGSE
TCGPPPYVVRAFHDSEGISKFPLGSQVTYRCQDGFTLTERFAHTRALCDHGERWEGPRMSCKPVECG
FPNTVENGRITGIDFTYPHTVQFLCNEGYRLVGNDSTTCQASGQWSAESPTCLAIDCPSLQAPVGGRI
RGSGNAFGTVLRFECDDGLQLEGSIERRCLQNGEWSGNDVKCTAEIDCGWPKNFWNGYVLGEKTTL
GSTLFFSCVVRTTFDGSYFETKCVSPGQWSNPPPTCWGQCSIPNIEFADINGYPVGTWINHGKTFEYT
CKNGLVPVHSSIVTCYNGTFNPGPPKCVPAPCPSPPPYVENGIRLFIGQEHGRRARYICNNGFRLSGM
TQSSPYLICDKGQWKGGNPACQEFYCPNPGTITNGKIYKKEPNNIRFNFPRYITTIKHGQRLEFKCDPG
YQISGASGATCINGQWIPPLGEAASHCKPQQYLPIPLRWTPIPNRNFYET
>Cg_P_selectin_02
MKPDCRIFTFLILSFTLFQDTQGNDCPERVPNPGRECQKACHPDDNPCKSGRKTCECDGLCGYSCIS
QNHRCENLARTIPNGRVNIKPQNMFGGIATYECDWGYEIRGQKDRVCRGDEKWEGEAPSCVLSKGS
GQPILSKEGCNSPPVIEYAIHNGRELGEGERYPSGTMLEYSCIEGFMRGSDYVERAWCIADGEWVGPK
MVCQAAGCPKPPEPLNGGVIIPASTGVNSVVRYYCNPGFELIGRSERICESDSTWTGNEPRCEKGGSE
TCGPPPYVVRAFHDSEGISKFPLGSQVTYRCQDGFTLTERFAHTRALCDHGERWEGPRMSCKPVECG
FPNTVENGRITGIDFTYPHTVQFLCNEGYRLVGNDSTTCQASGQWSAESPTCLAIDCPSLQAPVGGRI
RGSGNAFGTVLRFECDDGLQLEGSIERRCLQNGEWSGNDVKCTEIDCGWPKNFWNGYVLGEKTTLG
STLFFSCVVRTTFDGSYFETKCVSPGQWSNPPPTCWGQCSIPNIEFADINGYPVGTWINHGKTFEYTC
KNGLVPVHSSIVTCYNGTFNPGPPKCVPAPCPSPPPYVENGIRLFIGQEHGRRARYICNNGFRLSGMT
QSSPYLICDKGQWKGGNPACQEFYCPNPGTITNGKIYKKEPNNIRFNFPRYITTIKHGQRLEFKCDPGY
QISGASGATCINGQWIPPLGEAASHCKPQQYLPIPLRWTPIPNRNFYET
>Cg_P_selectin_03
MAHRIQWLKAVLVCALWKINACHYLQEKSEFCDVAYLSRRDPNLVYTPNVTQIQFGKTLQFSCLPGY
ALHGQEYGECMSDGRISIPGEFFCKDLGIRCGFDQLDKAHLTVTPKRTEYHYGEVVSLQCNEGFVLRG
PSTVSCTEDRLFDVPSNLACEAEIKCHKTNSILNVAHLVVTPDKEGYLYNETVSLQCRDGYTLQGSSQ
ATCLQNFEAPNDPSCLASYCDASWIYSRLSGVQYMSEEDPSRLEVGETIYFSCEKGYRLQGASSVKCL
PTGDLEGTGLLTQCERIRCSKESLVHGLYISPDKDSYDFNETVYFHCGEEQMVYGSHSARCAQNGWE
YDSPSLPMCGRSLCDTTWLHQEGMYTTPSIGGRVDVGTRIFINCYHGYYRRCTDTAFCMPNGRMEP
VGMMCHCQADRSSSPVPVK
>Cg_P_selectin_04
MVGIIRGLVFTIWTISMTTNGLSPPDFVSSWTSITAYSNPEVTITHGFGEMPMKLDVFVRVTHPVHGTV
TFSATGSGQTSDDPSKPFGGVIYLYNTNSVKIMAPGPSSCSPCSGGVAYFGDSGFSGPSSTSGVIVSGE
VMVRAWKTCTFVKPATISSAYPLSNAGNNYYQIPIGSAPDLVLVKAFFDDGWIMDAQGTVSLTEGSN
VGGVQYVFDVSNAYVRTPKDGKMFNGINWGSAGDLEYINGSVRVYTWSFQGLEYDPNLLTSSYSGS
TDPVTKTLTYDVHNSSIIMMVHASSRDPNHYGLTFPVSGTAMTDGGGVTGKYGSLFYAYDNNNQIF

FWKPSSGMGCHTMIGGAWSDHLLDGTTDCSNLPNIDIRFLQATIDELPCEEKGCSGTPAGPFCDCGG
TGMEGQYCSSRVACPPLIFGVDAIFATFIDMEYKFQDSVVFECMVGFEFSSGSDNRTCQADATWSGA
PYTCTPVPCPDVDPPILGVINGTYNKWFGEEVTFECNPGHDVLSGDFNRTCQANQTWTGTRLECALS
CGDPDPLPYNAHQTNFGNTYGVVATYACDQGHEYSSGDTAMLCQTTGQWNGTALNCSRVHCG
WPANGTNTNMTITGTQYQDKVFYHCFPGYEYYNIYNTLYYETFPRHCLHTAVWQPPIDCQKKVCGD
PGTPVRAALDHANSNVGNYEFMSIQRYNCYTGYEVISGSLQIECTEFGTWNDTVPTCDIRKCIHFGNV
TNGFVYYQGLEYLNHAVTVCDPGYALSSGDAMRMCDANGYWTGNVPVCQEVRVIYFIDPFDLNYT
LPSILELEELKNTVKIDPKNTSSYMRSLKSQNDPRPSAMAIGVSGLLIILGICAYICSLDLISHVRENGKVK
VTQGMPKNNTNVVAENGSSDKFAKKGKKRNQGSAMSVEDTPHVYA
>Cg_P_selectin_05
MSLPILSKEGCNSPPVIEYAIHNGRELGEGERYPSGTMLEYSCIEGFMRGSDYVERAWCIADGEWVGP
KMVCQAAGCPKPPEPLNGGVIIPASTGVNSVVRYYCNPGFELIGRSERICESDSTWTGNEPRCEKGGS
ETCGPPPYVVRAFHDSEGISKFPLGSQVTYRCQDGFTLTERFAHTRALCDHGERWEGPRMSCKPVEC
GFPNTVENGRITGIDFTYPHTVQFLCNEGYRLVGNDSTTCQASGQWSAESPTCLAIDCPSLQAPVGG
RIRGSGNAFGTVLRFECDDGLQLEGSIERRCLQNGEWSGNDVKCTAEIDCGWPKNFWNGYVLGEKT
TLGSTLFFSCVVRTTFDGSYFETKCVSPGQWSNPPPTCWGQCSIPNIEFADINGYPVGTWINHGKTFE
YTCKNGLVPVHSSIVTCYNGTFNPGPPKCVPAPCPSPPPYVENGIRLFIGQEHGRRARYICNNGFRLSG
MTQSSPYLICDKGQWKGGNPACQEFYCPNPGTITNGKIYKKEPNNIRFNFPRYITTIKHGQRLEFKCDP
GYQISGASGATCINGQWIPPLGEAASHCKPQQYLPIPLRWTPIPNRNFYET
>Cg_P_selectin_06
MTSYPDMVVVQAKPTATFAEFPGLISEGNGIPGCFTCGTEAGGLTFAYNASHVRVWVKKDHPITSAA
DGWGDRHYENNTRLVSYEVNIRVLAWNFDSDSNKCRWTWNSTIYANQQSTEAPRMIFPSPIKEDM
NSVLVLVSISPKGGANGGFLFYGVGSVVTSGDYHPDIQSNYSGLIFGYNQYGVFVWMVEPTSRSFFFD
IDFPWGSGEEQQQTNDVEINIHVFGLMSRGLCNTTGLSLTNGYLNIPYVRPCEREAIVCNYGFQASN
NSDTEARCNLDAEWNQTVLQCTETYCPDEFDLPNGAIIEKNLTVDGIIHYTCNLGFNISEGNLNRTCL
PDGTWSGYPPVCSEILCPDVTIPPNSEMVIRNYSVNGTLTYTCKAGFEHTFGNLSRKCLLNGSWSGDS
PICSQILCTNETNPVNGRIEEMNNSVNGSLLYSCDDGFEATSGNLSRECTLNGTWSGEPPVCAVITCP
ALTLPDNVGIIVHPPSSNGNQILSSIGTVTQQTYYTGSEAIYSCDPGYNHSGGDLTRNCLRNGTWSGD
PVMCNEIFCEPLTAMSIFVLSTRVSNSTGISVNTTATFSCNQGFRINGSSSLLCQIDGTWNGTQPNCL
VITCPALTLPDNVGIAVYPPSSNGSEPFSTNGTGTQQTYYTGSEAIYSCVPGFNHSGGDLTRTCLSNGT
WSGDPAICDEIFCEPLTTVSIFALSTNLSNSMGISVHTTATFSCNPGFQINGSNSLLCQIDGTWNGTQP
NCLEEVCQDPNTPQNSTLLSINRYLNGTAHFACNAGFQHTNGSLQRECLSSGEWSGVAPVCTFIREC
KCPCGMVHIPKYTDINDEKLAQEMEQMKKELKVLKNQTSAALRKKISVKDFRPSANASGAVWVVVL
MVMAGCIIVPDLIRALRHILSCCRPRS
>Cg_P_selectin_07
MTAYEPSFISDWRTAKSQDDHKVEFYVPFTDIKNATETHLPVKVIVEVKVEDGSDYYIFNAFGSSPRDD
DATEKYGGVIYFYNASGVLIFLPEKFENDPADIKNDDGKTVYLGIVDRWYHPLSNREQSIKNEFTDALV
RVKMWRRNDMPPDFDSGFQYNLNTLTTMSTATTDQPMETITYLEIPFTNMTSAPDMVVVQAKPPG
RFDELRGLVSEGNGMPACLRCGSDAGGLAFAYNTTHVRVWVKLNHPITSAADGWGDRHYQDKQR
LVSHEAAVRVLAWTFDNAPNRCRWTWNERTTINDASQDTPRMIFPGTKNPTEFLVLVTISPLNGPN
HGFQFYGAGSVVTSGDYNPDKPSRYSGLIFGYNQYGVFVWRVRPTTRSYTFDIDEPWGGGKEQHQT
NDVEIGIYVFGLMSRNSCNTTDLAVMNGYLNPSLLQPCENEAVVCNFGYHIENETHTVARCNILALW
NSNVPQCIETLCPDEIDPPNGIVLERNLSVNGVIQYACNERFNISAGNLNRTCFPNGTWSGIPPTCSEI
RCPSLLDPINGAVDISGTLIDGVATFSCNPGFTLTDGSLTRTCAPNGSWTGTSAECSEIFCPWIAVQDN

VIVGYHRDAAHNISVNTTAFFACQPGFSLSSYETITCLITGDWSADSPVCNEIFCPPVNASANSEMVIS
NHSVNSTLTYSCIDGFEHTFGNLHRTCLLDGTWSGDPPNCSEIFCPGLNHPLNGKAVAEQTSINSQAI
YSCDTGFNHTGGDLTRTCQLNGTWSGEPAICSEIFCTDIQTFENGDVTYSVVISSSSGVPINSTASFSC
DAGFRLSLDLNITCLVTGSWSEAPPVCSEILCPNVTDPGNARVESIDNKVNGSLLYSCNTDFEVTSGNL
SRVCTLNGTWSGGPPICSDITCLALPFPDNGRIDVSTQDPTLNTLYYPMGSQSSYAIGTQASYACNPG
FNPSRSDLTRTCLNNGTWDGNSVTCDEIFCSPLTISASTIMNLSTGVSKTNSMDISVNTSATFSCKPGF
RLNDSSSLLCQIDGTWNGTLPNCSEIYCPDLETPPNSTLTSIDKSVNGTAQFVCNKGYQQTNGSLQQ
TCLATGKWSGIELVCKAIGDCLCPCDFVTVPKYTNVSDVRLIQEIEKMQKELVVLKNQTSAALRKKICIK
DFRPSTNASGAVWVVFLIVLAGLIIIPDMLKAMRYILLNFFQNGHSSHRRFHKS
>Pm_P_selectin_01
MELRGLYLLSLMLVFTYANEDCPRRIDKNNGRTCKKKCQNGTCVSPRKDCLCDGACGKSCILRNMKC
TTLQKRIPHGSVQVVPYNMFGAVARYSCDDGYEISGVARRVCQGDGKWSNEEPECIVSSEVAVNTE
CGPPPVVHNARHDREPWRMSYEQGVMLQYECDSGFTSNRGSIFRAWCVRSGWVGPNMTCSHAG
CPLNNTTIENGGIIPPSQITTGAKLQYFCKHGFFLAGREERECLADGTWGGREPSCEKVTCGPPPIIEHAI
IMDIETGGTFSSGQQLTYACDQGYDMEGDPRAMCNTDGDWTWLGIRLMCRPINCGFPGNLVNGW
QSGHRFFFGETVNYHCNEGYELIGQRTRTCEENKKWSGFLPKCIAVECPILRAPLDGTMVGSGNAFGT
VLRFECIEGFKMSGSIERRCQADRTWSGEPVLCQEVNCRLPEPFYNGYVLGETTTVGSELFFSCNVRTN
FEGDSLSTRCLETGEWSDRIPTCWGQCEVPTILNASLEKYKEGEWVRSGRDLEYQCKNGLVPDVTNR
VRCYNGTWTSQPRCVPAACPQPPPHVDYGLRVFDGLRHNSRAKYICRNGFRLRGMPADNSFLTCKF
GLWTGGKPVCEEFYCPNPGTIANGKIYKNGLRAIFDFRHYIKTIRHGDRILFKCKEGFVLEGPSGATCVN
GQWRPPISDPDHKCKPATHAPFPKLWIPLEEMPET
